# Essential Oil Composition and Spatial Repellency against *Aedes aegypti* Using a High-Throughput Screening System

**DOI:** 10.1021/acsomega.6c04497

**Published:** 2026-07-25

**Authors:** João Paulo B. Sousa, Natália M.G. Magalhães, Naira S. Campos, Lais S. Morais, Thiago R.B. Mello, Wanessa G. Santiago, Ciro Martins Gomes, Nicole L. Achee, John P. Grieco, Lorena C. Albernaz, Laila S. Espindola

**Affiliations:** † Laboratório de Farmacognosia, 664468 Universidade de Brasília, Campus Universitário Darcy Ribeiro, Brasília 70910-900, Brazil; ‡ Programa de Pós-Graduação em Ciências Médicas, Universidade de Brasília, Campus Universitário Darcy Ribeiro, Brasília 70910-900, Brazil; § Department of Biological Sciences, Eck Institute for Global Health, 6111 University of Notre Dame, Notre Dame, Indiana 46556, United States

## Abstract

Essential oils present environmentally sustainable alternatives to conventional vector-control products. *Aedes aegypti* strongly impacts public health by transmitting the viruses responsible for dengue, Zika, chikungunya, and urban yellow fever. This study investigated the spatial repellency and chemical composition of 36 commercially available essential oils and 11 volatile compounds against the female *Ae. aegypti* mosquito using a high-throughput screening system (HITSS). Of the 36 essential oils evaluated, 30 commercial oils (83%) presented significant (*P* < 0.05) spatial repellency, of which 9 were classified as strong repellents (wSAI >50), 11 as medium repellents (20 < wSAI <50), and 10 as weak repellents (wSAI <20). Citronellol and menthol behaved as strong repellents, while the other 9 volatile compounds showed important repellent or attractant effects. In addition, the study identified 37 nonoxygenated compounds and 55 oxygenated compounds, totaling 92 components distributed in the 36 oils investigated. Of the 30 oils that modified *Ae. aegypti* behavior, 20 were strong/medium repellents, which are candidates for the development of new spatial repellent devices capable of creating vector-free areas. Additionally, our evaluation of 11 volatile compounds using the HITSS assay provides insights into better understanding structure-repellency relationships.

## Graphical abstract

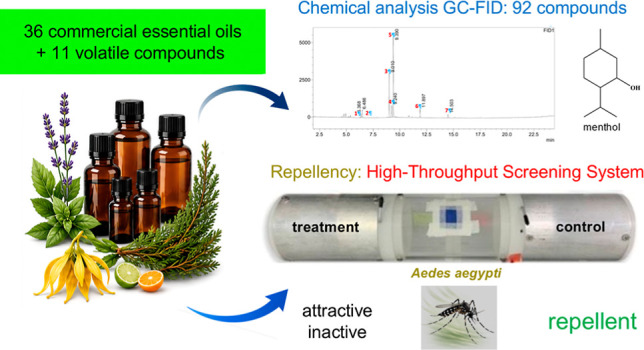

## 1 Introduction

Dengue, Zika, chikungunya, and urban yellow fever are important diseases transmitted by female *Aedes aegypti* mosquitoes in tropical and subtropical regions.^1^ These arboviruses have a strong negative impact on public health. As of April 2024, data published by the WHO^2^ indicated >7.6 million dengue cases, of which 3.4 million were confirmed cases, including >16 000 severe cases and >3000 deaths. A substantial increase in dengue cases has been observed globally over the last 5 years,^2^ necessitating hospital/outpatient care with the possibility of developing hemorrhagic manifestations or even culminating in death.^3^

People infected with the Zika virus typically present nonspecific symptoms such as an itchy rash, arthritis, and conjunctivitis. In more complex cases, they may lead to Guillain Barré syndrome. During pregnancy, the virus may affect fetal development, causing neurological conditions such as microcephaly.^4^ Individuals infected with chikungunya present symptoms including fever, swelling, myalgia, headache, nausea, fatigue, and skin rashes.^5^ Chikungunya may also cause persistent joint pain in addition to being associated with Guillain Barré syndrome.^4,5^ Yellow fever is a disease with a high-impact on public health and an associated risk of international spread.^6^

Temephos and malathion (organophosphates), deltamethrin (pyrethroid), and imidacloprid (neonicotinoid) are synthetic insecticides that are often used against *Ae. aegypti* and whose main mechanism of action is to induce insect death. These agents represent important strategies for mosquito control and thus prevent arbovirus transmission. That said, excessive use of synthetic insecticides results in numerous problems.^7^ Indeed, mosquito resistance has become increasingly serious with prolonged chemical pesticide application^8^ in addition to environmental pollution and consequent ecological imbalance.^9^

From another perspective, some compounds affect insect behavior with other mechanisms of action, such as contact irritation and spatial repellency. These effects should also be quantified and evaluated to develop vector management products.^10,11^ Topical repellents applied directly to the skin result in limited repellency.^11^ Differently, spatial repellency induces an oriented movement of mosquitoes in the opposite direction to a chemical stimulus, without the need for physical contact between the insect and treated surface.^12,13^ Spatial repellents are designed to create vector-free spaces by releasing active ingredients into the environment. Knowledge about spatial repellents enables an understanding of the product mode of action in altering vector behavior to prevent arbovirus transmission.^12^ Furthermore, increased cases of the aforementioned diseases, together with increased vector resistance to conventional synthetic insecticides, necessitate new strategies to reduce mosquito populations and decrease human-vector contact.^13^

Spatial repellents may be produced from commercially available active ingredients. This strategy could contribute to reducing production costs and accelerating routine use.^14^ Synthetic compounds used in *Ae. aegypti* control, such as a number of pyrethroids, have demonstrated significant spatial repellent activity.^15^ Clinical field studies evaluated the efficiency of a device that passively emanates transfluthrin, an insecticide of the pyrethroid class. This active ingredient is already marketed worldwide as an adulticide. These studies sought to prove the epidemiological effects of this active ingredient as a spatial repellent on the incidence of malaria and dengue in the regions tested.^16–19^

An alternative to synthetic insecticides is the development of commercially available essential oils as spatial repellents. Plant essential oils constitute environmentally friendly alternatives^20^ due to their biodegradability, insect selectivity, and low vertebrate toxicity.^21^ Usually, the major components of essential oils are monoterpenes and sesquiterpenes, including alcohol, aldehyde, phenol, ester, ether, ketone, and oxide derivatives.^22^ These volatile compounds play an important role in insect-plant interactions and are part of defense strategies against herbivorous insects.^20^ The literature indicates that *catnip* oil and geraniol reduced horn fly feeding in a laboratory bioassay and exhibited spatial repellency,^23^ while essential oils acted as spatial repellents for pestiferous social wasps.^24^ Another study reported spatial releasing properties and mosquito repellency assessed using cellulose-based beads containing essential oils,^25^ with *Croton tetradenius* essential oil demonstrating spatial repellency against *Ae. aegypti*.^26^ In this context, the present study reports the spatial repellency and chemical composition of 36 commercially available essential oils and 11 volatile compounds against *Ae. aegypti*.

## 2 Materials and Methods

### 2.1 Chemicals and Essential Oil Samples

The volatile compounds borneol, carvacrol, 1, 8-cineole, citral, citronellal, citronellol, eugenol, geraniol, menthol, nerol, sabinene, linalool, together with transfluthrin and the homologous series of C_8_–C_20_ *n*-alkanes were purchased from Sigma-Aldrich (Brazil, China, and Switzerland). All other chemicals used in this study were of gas chromatography grade and are commercially available. Authentic essential oil samples (common name) were acquired from a specialized oils and natural cosmetics company (BioEssência, Jaú City, São Paulo State, Brazil). Regarding this company, agricultural production and essential oil extraction occur at the BioEssência Farm, located in Rio Preto da Eva City, Amazonas State, Brazil. All production undergoes rigorous analysis to support the seal of the Biodynamic Institute for Rural Development - BID, the largest organic certifier in Latin America, guaranteeing that the products are totally natural, non-GMO, and not tested on animals. In addition, all products have the necessary licenses and registration with ANVISA - the Brazilian Health Regulatory Agency. In this company, the essential oils were obtained using industrial-scale steam-distillation. The leaves and aerial parts of each plant were crushed by a mill and submitted to the distillation process. The resulting essential oils were stored directly in amber bottles and made available for commercial scale. The oils used were *Amyris balsamifera* bark oil (balsam torchwood), *Anthemis nobilis* (Roman chamomile), *Boswellia carteri* (Frankincense), *Cananga odorata* (ylang ylang), *Cedrus atlantica* (atlas cedar), *Citrus aurantium* (sour orange), *Citrus aurantium amara* (petitgrain), *Citrus aurantium bergamia* (bergamot), *Citrus aurantium dulcis* (sweet orange), *Citrus limon* (lemon), *Citrus paradisi* (grapefruit), *Citrus reticulata* (mandarin orange), *Copaifera reticulata* (copaiba), *Cupressus sempervirens* (cypress), *Cymbopogon flexuosus* (lemongrass), *Cymbopogon martini* (palm rose), *Cymbopogon nardus* (citronella), *Eucalyptus citriodora* (lemon-scented gum), *Eucalyptus globulus* (blue gum), *Eucalyptus staigeriana* (lemon-scented ironbark), *Eugenia caryophyllus* (clove), *Foeniculum vulgare* (fennel), *Juniperus communis* (juniper), *Lavandula angustifolia* (English lavender), *Lavandula hybrida* (lavandin), *Litsea cubeba* (mountain pepper), *Melaleuca alternifolia* (tea tree), *Mentha piperita* (peppermint), *Ocimum basilicum* (basil), *Origanum majorana* (marjoram), *Pelargonium graveolens* (geranium), *Pogostemon cablin* (patchouli), *Rosmarinus officinalis* (rosemary), *Salvia sclarea* (sage), *Vetiveria zizanioides* (vetiver), and *Zingiber officinale* (ginger).

### 2.2 Chromatographic Analyses

All essential oils, volatile compounds, and *n*-alkane series samples were dissolved separately in hexane, giving the respective final concentrations: 100–140 μg/mL, 60–80 μg/mL, and 60 μg/mL, respectively. Posteriorly, 1 μL of each sample was analyzed by gas chromatography (GC-FID) in a Shimadzu GC-2010 Pro equipped with split/splitless injector inlet and automatic sampler AOC 20Si Plus. Injector temperature was maintained at 250 °C, operating with a 50:1 split ratio, 1.0 mL/min nitrogen carrier gas flow, and detector temperature fixed at 280 °C. Chromatographic separations were achieved using a fused-silica capillary column (SH-5, 30 m × 0.25 mm × 0.25 μm) and an oven programmed with a linear gradient from 50 to 260 °C and a 25 min run time. The relative percentage of each peak was obtained using the chromatographic peak area ratio. The identification of essential oil constituents was made by comparison of the calculated Kovats retention index of each volatile with those described in the literature^27^ and a comparative study using the University of Brasilia (UnB) analytical center standards library.

### 2.3 Mosquito Rearing, Spatial Repellency Assay, and Data Analyses

*Ae. aegypti* (Rockefeller) colonies were established in the UnB ArboControl Insectarium. Mosquitoes were maintained at 27 °C, 80% relative humidity, with a 12:12 h light-dark photoperiod. Adult insects were fed with a 10% sugar solution and given an equine blood meal (supplied by the UnB Veterinary Hospital) 3 times per week to stimulate egg production. After hatching, larvae were reared in trays with 4 L of tap water and fed on a diet of ground fish chow. Pupae were sorted into males and females. Adults for the assays were fed with 10% sugar solution only. For the spatial repellency assay, groups of 20 ± 2 females, 4–7 days old, were separated the day before the test and maintained without food.

An aliquot of 1.5 mL of solution of each essential oil (10% in acetone v/v) or volatile compound (1% in acetone v/v) was impregnated in a 275 cm^2^ polyester netting sheet. Excess solvent was allowed to evaporate for 15 min prior to the testing. The same procedure was performed for acetone in the control net. A new treatment net was replaced at the fifth replicate to ensure essential oil or compound concentration in all replicates.

The spatial repellency assay device, also known as the high-throughput screening system^12^ (HITSS, Figure 1), is composed of 3 chambers: a central transparent plastic chamber without netting, connected to 2 metal chambers with netting configured as either a treatment or control. Each metal chamber is connected to the central chamber by an open/close butterfly valve. An essential oil or compound-treated netting strip was placed in treatment chamber covering all of the inner area (11 × 25 cm) and an untreated net (acetone only) similarly placed in the opposite control chamber. The acetone control netting strip was treated first using a separated metal tray and pipet inside a fume hood to avoid any contamination with the essential oils. A group of (20 ± 2) female mosquitoes was transferred to the central chamber through the mosquito entrance. Black felt was used to darken the central chamber and the mosquitoes were allowed to acclimatize for 30 s prior to assay initiation. The 2 butterfly valves were opened allowing the mosquitoes to fly freely throughout the 3 compartments for 10 min. Finally, the valves were simultaneously closed and the mosquitoes counted in each compartment, including those knocked down (KD). The 3 chambers were separated and ventilated for 3 min between replicates. A total of 9 replicates were performed for each sample, with all tests conducted between 8 am and 6 pm on the same day. At the end of each test, the metal chambers were cleaned with acetone, while the other components were washed with Extran detergent solution (Merck KGaA, Darmstadt, Germany) and rinsed with tap water. All system parts were air-dried overnight.

**Figure 1:**
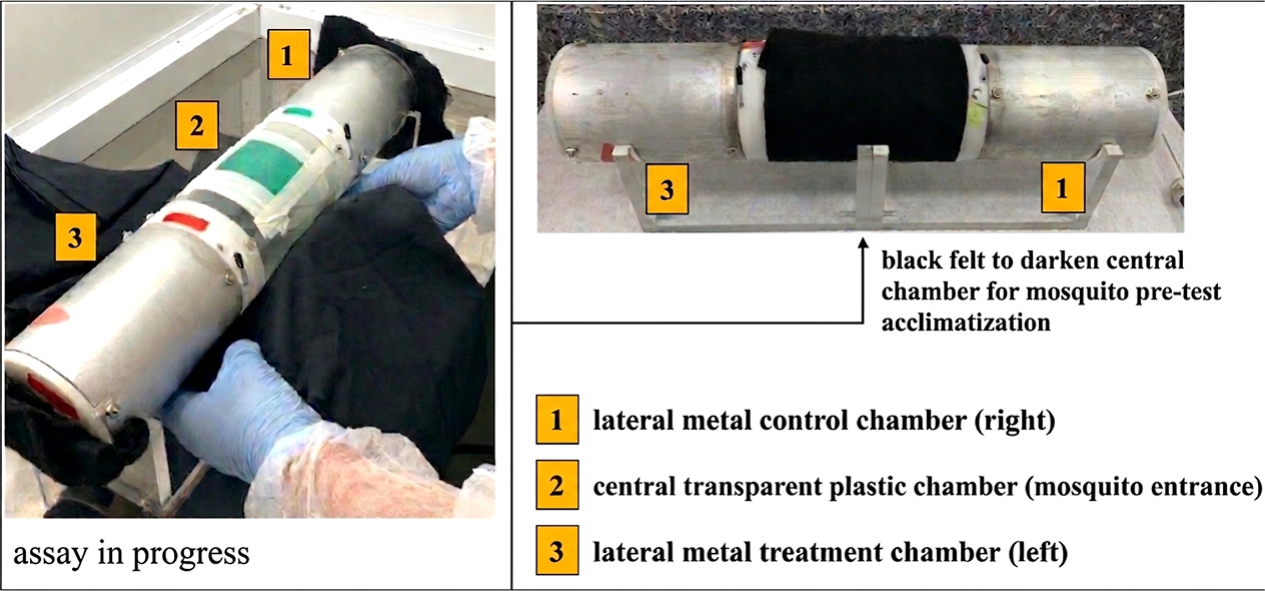
Characteristics of the high-throughput screening system (HITSS)—configuration during the spatial repellency assay.

The spatial repellency activity index (SAI) measuring mosquito response in the assay was calculated using eq 1:

1 $$SAI=\frac{\left(Nc-Nt\right)}{\left(Nc+Nt\right)}$$

Nc is the number of mosquitoes in the control chamber and Nt corresponds to the number of mosquitoes in the treatment chamber. The SAI value ranges from −1 to 1. Negative values indicate attractant activity, positive values represent repellent action, while zero corresponds to no response. The weighted spatial activity index (wSAI) was also calculated to rank the sample activity. The wSAI was calculated by multiplying the SAI value by the percentage of mosquitoes that responded in the assay, choosing either the control or the treatment chamber. The percentage responding was obtained by eq 2:

2 $$\%RESP=\frac{\left(Nc+Nt\right)}{N}\times 100$$

*N* is the total number of mosquitoes inserted into the HITSS apparatus. On the basis of studies conducted with 10% linalool,^15^ our research group established a classification system according to the wSAI value, which was adopted in the present study: strong repellency (wSAI >50), medium repellency (20 < wSAI <50), and weak repellency (wSAI <20). Similarly, using 1% transfluthrin as a reference,^15^ we also decided to classify volatile compounds tested at 1% as strong repellency (wSAI >30), medium repellency (10 < wSAI <30), and weak repellency (wSAI <10).

## 3 Results

### 3.1 Chemical Composition

The GC-FID methodology was reproducible, enabling the obtention of 47 chromatographic profiles from the essential oils (GC-FID chromatographic profiles of the studied essential oils; Figures S1–S36) and from the volatile constituents (GC-FID chromatographic profiles of the volatile compounds; Figures S37–S47). Using the analytical parameters, it was possible to identify 37 nonoxygenated compounds (Figure 2) and 55 oxygenated compounds (Figure 3), totaling 92 components present in the 36 commercial oil samples. Table 1 lists the name, retention time, Kovats retention index, and relative percentage for each compound in each studied species.

**Figure 2:**
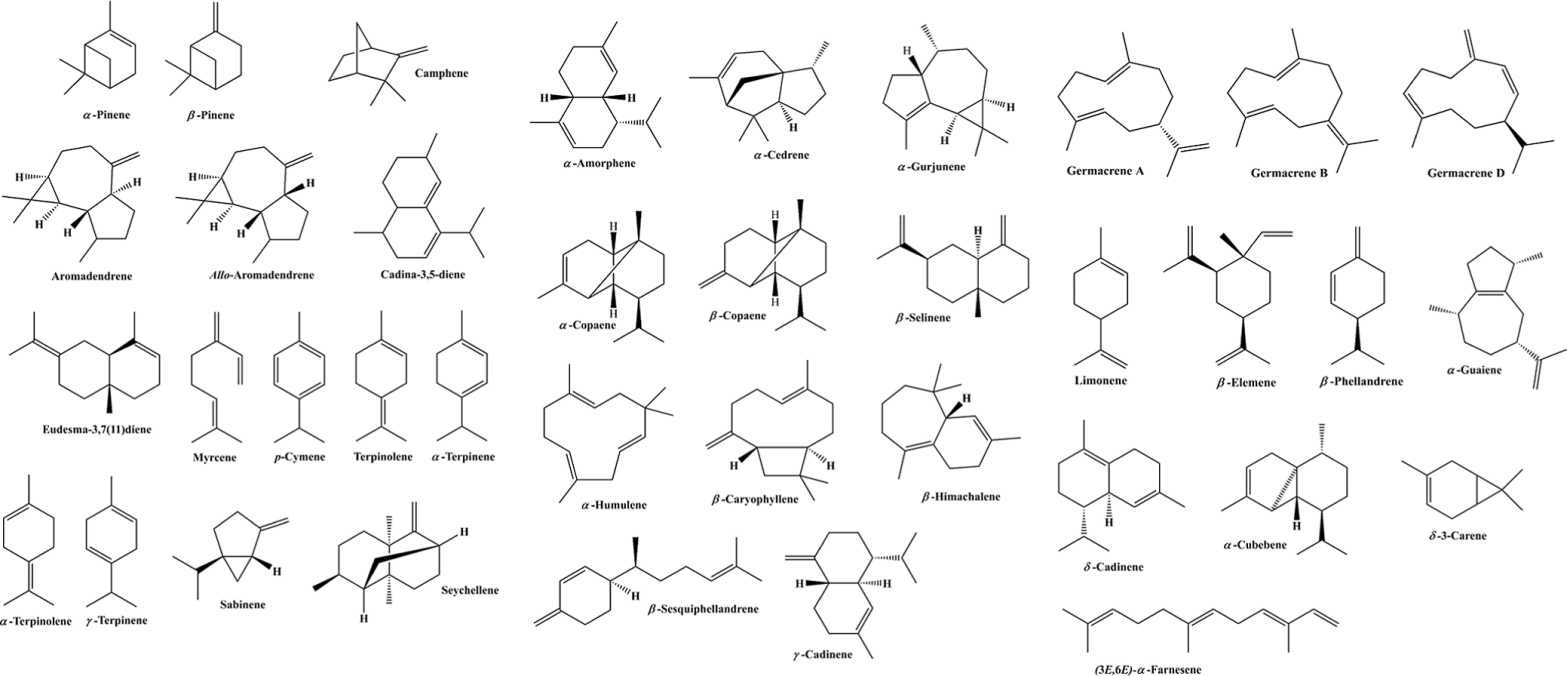
Chemical structures of 37 nonoxygenated compounds identified in the essential oils.

**Figure 3:**
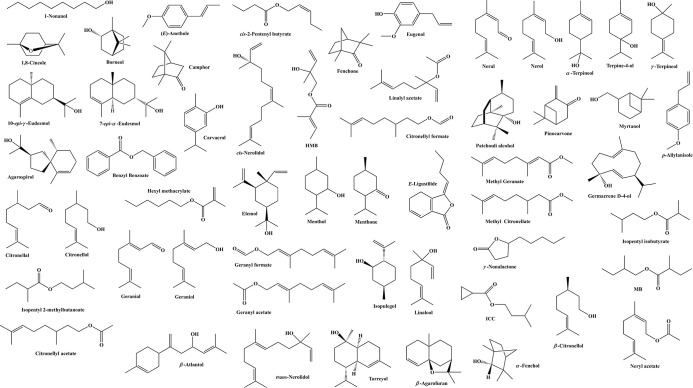
Chemical structures of 55 oxygenated compounds identified in the essential oils. ICC: isopentyl cyclopropanecarboxylate. MB: 2-methylbutyl 2-methylbutanoate. HMB: 2-hydroxy-2-methyl-but-3-enyl 2-methyl-2­(*Z*)-butenoate.

**Table 1:** Chemical Characteristics of the Commercially Available Essential Oils Evaluated^a^

N°	Compound	RT	ikexp	iklit	(%)	N°	compound	RT	ikexp	iklit	(%)
*Amyris balsamifera* bark oil (balsam torchwood)							*Anthemis nobilis* (Roman chamomile)				
**1**	β-himachalene	15.2	1471	1470	4.09	**1**	isopentyl isobutyrate	6.1	1018	1017	1.47
**2**	β-agarofuran	15.6	1490	1491	5.74	**2**	ICC	6.8	1053	1051	20.76
**3**	*cis*-nerolidol	15.8	1502	1502	5.96	**3**	*cis*-2-pentenyl butyrate	7.1	1068	1068	5.04
**4**	eudesma-3, 7(11)diene	16.4	1532	1534	6.06	**4**	isopentyl 2-methylbutanoate	7.8	1102	1108	24.51
**5**	*trans*-nerolidol	16.9	1561	1564	13.43	**5**	MB	8.0	1112	1112	4.52
**6**	agarospirol	18.2	1639	1639	8.31	**6**	camphor	8.8	1151	1151	3.93
**7**	7-*epi*-α-eudesmol	18.4	1649	1651	9.23	**7**	hexyl methacrylate	8.9	1156	1156	11.59
**8**	torreyol	18.7	1671	1670	29.89	**8**	pinocarvone	9.2	1171	1170	2.24
						**9**	HMB	11.0	1258	1257	15.86
						**10**	β-selinene	15.7	1497	1499	1.85
*Boswellia carteri* (Frankincense)						*Cananga odorata* (ylang ylang)					
**1**	α-pinene	4.6	936	935	53.65	**1**	*p*-cymene	6.2	1024	1024	3.03
**2**	sabinene	5.3	976	975	2.91	**2**	linalool	7.8	1101	1103	6.34
**3**	myrcene	5.6	992	992	4.66	**3**	α-fenchol	8.2	1120	1120	2.86
**4**	*p*-cymene	6.3	1028	1028	3.69	**4**	1-nonanol	9.2	1170	1171	5.48
**5**	limonene	6.4	1032	1032	24.31	**5**	β-citronellol	10.5	1233	1233	1.80
**6**	β-caryophyllene	14.5	1432	1432	10.78	**6**	linalyl acetate	11.1	1259	1259	2.89
						**7**	neryl acetate	13.2	1365	1368	2.29
						**8**	geranyl acetate	13.6	1387	1387	3.09
						**9**	β-caryophyllene	14.4	1426	1426	10.60
						**10**	β-copaene	14.5	1432	1432	6.83
						**11**	aromadendrene	14.7	1445	1446	30.50
						**12**	germacrene D	15.7	1494	1494	8.23
						**13**	γ-cadinene	16.0	1513	1513	7.71
						**14**	benzyl benzoate	20.3	1783	1784	6.63
*Cedrus atlantica* (atlas cedar)						*Citrus aurantium* (sour orange)					
**1**	α-cubebene	15.1	1436	1463	23.10	**1**	sabinene	5.3	976	975	0.21
**2**	germacrene D	15.7	1494	1494	15.95	**2**	β-pinene	5.4	981	981	0.42
**3**	γ-cadinene	16.0	1514	1514	57.38	**3**	myrcene	5.6	992	992	1.73
**4**	e-ligustilide	20.4	1788	1790	3.57	**4**	limonene	6.4	1032	1032	97.30
*Citrus aurantium amara* (petitgrain)						*Citrus aurantium bergamia* (bergamot)					
**1**	linalool	7.8	1102	1103	27.99	**1**	limonene	6.4	1032	1032	32.93
**2**	α-terpineol	9.8	1197	1197	6.28	**2**	linalool	7.8	1103	1103	18.98
**3**	linalyl acetate	11.1	1258	1259	61.30	**3**	linalyl acetate	11.1	1258	1259	40.74
**4**	geranyl acetate	13.6	1387	1387	4.43						
*Citrus aurantium dulcis* (sweet orange)							*Citrus limon* (lemon)				
**1**	limonene	6.4	1032	1032	99.00	**1**	β-pinene	5.4	981	981	13.18
						**2**	*p*-cymene	6.3	1028	1028	3.79
						**3**	limonene	6.4	1032	1032	77.19
						**4**	γ-terpinene	7.0	1062	1060	5.83
*Citrus paradisi* (grapefruit)						*Citrus reticulata* (mandarin orange)					
**1**	limonene	6.4	1032	1032	95.00	**1**	limonene	6.4	1031	1032	62.94
						**2**	γ-terpinene	7.0	1062	1060	4.78
						**3**	γ-nonalactone	12.4	1325	1328	2.63
						**4**	germacrene B	17.0	1569	1569	12.80
*Cupressus sempervirens* (cypress)						*Cupressus sempervirens* (cypress)					
**1**	α-copaene	13.6	1385	1377	6.86	**1**	α-pinene	4.6	936	935	67.79
**2**	α-cedrene	13.9	1400	1400	1.62	**2**	δ-3-carene	6.0	1014	1012	32.20
**3**	β-caryophyllene	14.5	1431	1432	63.07						
**4**	aromadendrene	14.7	1443	1447	4.98						
**5**	α-humulene	15.1	1466	1462	8.29						
**6**	germacrene D	15.7	1493	1488	11.00						
**7**	δ-cadinene	16.4	1534	1530	4.17						
*Cymbopogon martini* (palm rose)						*Cymbopogon martini* (palm rose)					
**1**	camphene	4.8	946	948	1.55	**1**	linalyl acetate	11.1	1258	1259	89.68
**2**	myrcene	5.6	991	992	1.43	**2**	geranyl acetate	13.6	1387	1387	10.32
**3**	limonene	6.4	1032	1032	1.10						
**4**	linalool	7.8	1103	1103	2.26						
**5**	neral	10.8	1247	1240	30.80						
**6**	geraniol	11.1	1259	1257	4.67						
**7**	geranial	11.4	1276	1276	48.61						
**8**	β-elemene	13.6	1387	1384	4.98						
**9**	β-caryophyllene	14.5	1432	1432	4.59						
*Cymbopogon nardus* (citronella)						*Eucalyptus citriodora* (lemon-scented gum)					
**1**	limonene	6.4	1032	1032	4.33	**1**	isopulegol	8.8	1152	1153	6.00
**2**	citronellal	8.9	1156	1156	45.98	**2**	citronellal	8.9	1156	1156	83.54
**3**	nerol	10.5	1231	1230	14.60	**3**	citronellol	10.5	1232	1232	10.46
**4**	geraniol	11.0	1258	1257	24.81						
**5**	citronellyl acetate	13.0	1356	1355	1.40						
**6**	eugenol	13.2	1365	1362	0.78						
**7**	geranyl acetate	13.6	1387	1385	1.54						
**8**	β-elemene	13.9	1400	1397	1.23						
**9**	δ-cadinene	16.4	1534	1530	1.36						
**10**	elemol	16.9	1561	1558	2.50						
**11**	germacrene D-4-ol	17.4	1590	1583	1.47						
*Eucalyptus globulus* (blue gum)						*Eucalyptus staigeriana* (lemon-scented ironbark)					
**1**	limonene	6.4	1032	1032	6.99	**1**	limonene	6.4	1032	1032	31.26
**2**	1, 8-cineole	6.5	1036	1035	93.01	**2**	α-terpinolene	7.6	1093	1090	10.24
						**3**	nerol	10.5	1233	1234	2.26
						**4**	neral	10.8	1247	1248	15.24
						**5**	methyl citronellate	11.0	1259	1257	7.51
						**6**	geranial	11.4	1276	1276	21.52
						**7**	methyl geranate	12.5	1328	1330	6.05
						**8**	geranyl acetate	13.6	1387	1388	5.91
*Eugenia caryophyllus* (clove)							*Foeniculum vulgare* (fennel)				
**1**	eugenol	13.2	1365	1363	84.71	**1**	limonene	6.4	1032	1032	2.72
**2**	β-caryophyllene	14.5	1432	1432	9.19	**2**	fenchone	7.6	1095	1095	11.66
**3**	α-humulene	15.1	1467	1462	2.24	**3**	(*E*)-anethole	11.8	1292	1292	85.62
*Juniperus communis* (juniper)						*Lavandula angustifolia* (English lavender)					
**1**	α-pinene	4.6	936	935	44.42	**1**	linalool	7.8	1103	1103	37.67
**2**	sabinene	5.3	976	975	13.37	**2**	linalyl acetate	11.1	1258	1259	62.33
**3**	β-pinene	5.4	981	981	9.95						
**4**	myrcene	5.6	993	992	12.10						
**5**	p-cymene	6.3	1028	1028	4.17						
**6**	limonene	6.4	1032	1032	4.08						
**7**	terpine-4-ol	9.5	1185	1185	4.73						
**8**	β-copaene	14.5	1432	1432	4.72						
*Lavandula hybrida* (lavandin)						*Litsea cubeba* (mountain pepper)					
**1**	1, 8-cineole	6.5	1037	1038	5.84	**1**	sabinene	5.3	976	977	8.74
**2**	linalool	7.8	1102	1103	36.34	**2**	β-pinene	5.4	981	981	5.07
**3**	camphor	8.8	1153	1151	8.18	**3**	myrcene	5.6	991	992	2.60
**4**	borneol	9.3	1174	1172	4.43	**4**	1, 8-cineole	6.5	1032	1032	18.14
**5**	terpinen-4-ol	9.5	1184	1184	4.44	**5**	neral	10.8	1246	1240	26.58
**6**	myrtanol	11.0	1258	1257	34.48	**6**	geranial	11.4	1276	1276	37.29
**7**	(*E*)-anethole	11.8	1293	1292	2.88	**7**	β -caryophyllene	14.5	1431	1432	1.57
*Melaleuca alternifolia* (tea tree)						*Mentha piperita* (peppermint)					
**1**	α-terpinene	6.1	1020	1022	9.88	**1**	limonene	6.4	1032	1032	2.20
**2**	p-cymene	6.3	1028	1028	5.30	**2**	β-phellandrene	6.5	1038	1038	4.38
**3**	1, 8-cineole	6.5	1038	1038	2.56	**3**	menthone	9.0	1160	1160	26.52
**4**	γ-terpinene	7.0	1062	1060	23.54	**4**	α-terpineol	9.2	1171	1171	9.46
**5**	α-terpinolene	7.6	1093	1090	3.74	**5**	menthol	9.4	1178	1178	49.45
**6**	terpinen-4-ol	9.5	1184	1184	51.68	**6**	carvacrol	12.0	1299	1299	5.56
**7**	α-terpineol	9.8	1198	1198	3.30	**7**	α-gurjunene	14.5	1432	1434	2.43
*Ocimum basilicum* (basil)						*Origanum majorana* (marjoram)					
**1**	linalool	7.8	1103	1103	21.15	**1**	sabinene	5.3	976	975	6.85
**2**	γ-terpineol	9.4	1179	1178	0.52	**2**	p-cymene	6.3	1028	1028	12.40
**3**	p-allylanisole	9.9	1204	1203	75.01	**3**	γ-terpinene	7.0	1062	1060	4.80
**4**	geranial	11.4	1276	1276	0.57	**4**	terpinolene	7.2	1072	1072	7.62
**5**	cadina-3, 5-diene	14.7	1444	1444	0.66	**5**	linalool	7.8	1104	1103	28.78
**6**	germacrene B	16.7	1550	1550	1.51	**6**	terpine-4-ol	9.5	1184	1185	34.91
						**7**	α-terpineol	9.8	1198	1198	4.65
*Pelargonium graveolens* (geranium)						*Pogostemon cablin* (patchouli)					
**1**	α-pinene	4, 6	936	936	6.76	**1**	β-elemene	13.8	1393	1390	2.85
**2**	limonene	6.4	1032	1032	7.85	**2**	β-caryophyllene	14.4	1426	1426	0.49
**3**	linalool	7.8	1103	1103	11.54	**3**	α-guaiene	14.5	1432	1433	3.65
**4**	α-fenchol	8.2	1120	1120	4.43	**4**	seychellene	14.8	1448	1446	16.45
**5**	α-terpineol	9.8	1198	1198	3.67	**5**	α-humulene	15.0	1458	1458	7.62
**6**	β-citronellol	10.5	1232	1233	35.75	**6**	γ-cadinene	16.0	1511	1513	3.41
**7**	linalyl acetate	11.1	1259	1259	11.87	**7**	α-amorfene	16.1	1518	1519	20.79
**8**	citronellyl formate	11.5	1279	1280	5.35	**8**	β-atlantol	17.6	1600	1608	0.88
**9**	geranyl formate	12.0	1306	1305	1.97	**9**	patchouli alcohol	19.0	1683	1683	36.63
**10**	citronellyl acetate	13.3	1368	1368	3.76						
**11**	α-copaene	13.6	1387	1387	7.05						
*Rosmarinus officinalis* (rosemary)						*Salvia sclarea* (sage)					
**1**	α-pinene	4.6	936	935	26.28	**1**	linalool	7.8	1103	1103	23.88
**2**	camphene	4.9	952	952	9.25	**2**	α-terpineol	9.8	1198	1198	3.24
**3**	β-pinene	5.4	981	981	6.20	**3**	linalyl acetate	11.1	1259	1259	66.15
**4**	myrcene	5.6	993	992	2.15	**4**	neryl acetate	13.3	1368	1368	1.31
**5**	p-cymene	6.3	1027	1028	3.01	**5**	geranyl acetate	13.6	1387	1387	2.81
**6**	1–8-cineole	6.5	1037	1038	21.11	**6**	β-copaene	14.5	1432	1432	1.42
**7**	camphor	8.8	1153	1147	26.22	**7**	germacrene D	15.7	1494	1494	1.19
**8**	borneol	9.3	1174	1172	3.11						
**9**	α-terpineol	9.8	1198	1198	2.67						
*Vetiveria zizanioides* (vetiver)						*Zingiber officinale* (ginger)					
**1**	10-epi-γ-eudesmol	17.9	1622	1623	85.00	**1**	camphene	4.9	952	952	6.62
						**2**	limonene	6.4	1033	1032	5.88
						**3**	β-phellandrene	6.5	1038	1038	2.86
						**4**	allo-aromadendrene	15.6	1490	1489	10.90
						**5**	germacrene A	15.8	1502	1503	39.62
						**6**	γ-cadinene	16.0	1512	1512	9.57
						**7**	(3e,6e)-α-farnesene	16.1	1516	1515	8.50
						**8**	β-sesquiphellandrene	16.4	1533	1539	16.05

a **N°** = peak number. **RT**: retention time (min.), **ikexp**: experimental retention index (Kovats). **iklit**: literature retention index (Kovats). **ICC**: isopentyl cyclopropanecarboxylate. **MB**: 2-methylbutyl 2-methylbutanoate. **HMB**: 2-hydroxy-2-methyl-but-3-enyl 2-methyl-2­(*Z*)-butenoate.

Observing Table 1, the analyses of the 7 *Citrus* derivatives samples determined from 1 to 4 constituents, showing prominent limonene contents (33–99%) followed by linalyl acetate (40–61%) and linalool (19–28%). In the *C. atlantica* oil, 3 volatiles were relevant, including α-cubebene (23%), germacrene D (16%), and γ-cadinene (57%). In *E. globulus* and *E. citriodora,* 1, 8-cineole (93%) and citronellal (83%) were the major essential oils, respectively. The *E. staigeriana* sample diplayed 8 volatiles, the most expressive being limonene (31%), geranial (21%), neral (15%) and α-terpinolene (10%). Two volatiles were observed in *C. martini* oil, linalyl acetate (89%) and geranyl acetate (10%). The most present compounds, between 9 and 11 were geranial (48%) and neral (30%) in *C. flexuosus* oil and citronellal (46%), geraniol (24%), and nerol (14%) in *C. nardus* oil. While only one component, 10-*epi*-γ-eudesmol (85%), was identified in vetiver oil, 14 were detected in ylang ylang oil, in which the major compounds were β-caryophyllene (10%) and aromadendrene (30%). The *Lavandula* oils were distinct from each other: linalool (37%) and linalyl acetate (62%) were found in *L. angustifolia* and 7 compounds were identified in *L. hybrida,* with linalool (36%) and myrtanol (34%) the most present.

Seven components were identified in *Copaifera reticulata, L. cubeba, M. alternifolia, M. piperita, O. majorana,* and *S. sclarea*, with α-terpineol common to *M. alternifolia, M. piperita, O. majorana,* and *S. sclarea* oils; 1, 8-cineole in *L. cubeba* and *M. alternifolia;* linalool in *O. majorana* and *S. sclarea*, and β-caryophyllene in *Copaifera reticulata* and *L. cubeba*. *E*-anethole (85%), eugenol (84%), and α-pinene (67%) were the significant volatiles in the *F. vulgare, E. caryophyllus,* and *C. sempervirens* oils, respectively. Between 6 and 11 constituents were identified in the *A. balsamifera, A. nobilis, B. carteri, J. communis, O. basilicum, P. graveolens, P. clabin, R. officinalis,* and *Z. officinale* oils. In these samples, the highlights were *trans*-nerolidol (13%), torreyol (29%), isopentyl cyclopropanecarboxylate (ICC, 20%), isopentyl 2-methylbutanoate (24%), hexyl methacrylate (11%), 2-hydroxy-2-methyl-but-3-enyl 2-methyl-2­(*Z*)-butenoate (HMB, 15%), α-pinene (53%), limonene (24%), β-caryophyllene (10%), sabinene (13%), myrcene (12%), *p*-allylanisole (75%), linalool (21%), β-citronellol (35%), linalyl acetate (12%), seychellene (16%), α-amorphene (20%), patchouli alcohol (36%), 1, 8-cineole (21%), camphor (26%), *allo*-aromadendrene (10%), germacrene A (39%), and β-sesquiphellandrene (16%).

### 3.2 Spatial Repellency Activity

The findings listed in Table 2 demonstrated that 30 commercial oils presented spatial repellency activity, with *p*-values from the spatial activity index (SAI) < 0.05, corresponding to 83% of the samples studied. Oil classification according to the wSAI criteria determined 9 strong repellents; 11 medium repellents, and 10 weak repellents (Table 2).

**Table 2:** Spatial Repellency Activity of the 36 Essential Oils against *Aedes aegypti* Females (Dose 10% v/v)

Species studied (common name) [repellency level]	^a^N°	Mean % responding (±SE^b^)	Mean SAI^c^ (±SE^b^)	SR^d^ SAI^c^	*P* > S SAI^c^	wSAI^e^ (±SE^b^)	SR^d^ wSAI^e^	*P* > S wSAI^e^
*Amyris balsamifera* bark oil (balsam torchwood)^g^	9 (183)	10.431 (2.420)	0.481 (0.236)	9.500	0.125	7.092 (3.022)	11.0	0.078
*Anthemis nobilis* (Roman chamomile) [medium]	9 (175)	37.759 (9.020)	0.700 (0.145)	14.000	0.016	30.946 (9.182)	14.0	0.016
*Boswellia carteri* (Frankincense) [weak]	9 (176)	20.267 (4.428)	0.926 (0.074)	22.500	0.004	19.209 (4.723)	22.5	0.004
*Cananga odorata* (ylang ylang) [weak]	9 (177)	10.763 (2.607)	0.667 (0.144)	14.000	0.016	8.541 (1.956)	14.0	0.016
*Cedrus atlantica* (atlas cedar) [weak]	9 (170)	28.467 (5.042)	0.418 (0.114)	10.500	0.031	15.517 (5.335)	10.5	0.031
*Citrus aurantium* (sour orange) [weak]	9 (178)	28.058 (3.117)	0.635 (0.115)	18.000	0.008	16.809 (3.751)	18.0	0.008
*Citrus aurantium amara* (petitgrain) [strong]	9 (179)	65.137 (5.810)	0.907 (0.032)	22.500	0.004	58.464 (5.340)	22.5	0.004
*Citrus aurantium bergamia* (bergamot) [strong]	9 (178)	50.324 (12.203)	1.000 (0.000)	22.500	0.004	50.324 (12.203)	22.5	0.004
*Citrus aurantium dulcis* (sweet orange) [weak]	9 (177)	16.275 (2.635)	0.689 (0.229)	17.000	0.039	12.824 (3.203)	21.5	0.008
*Citrus limon* (lemon)^g^	9 (183)	23.089 (1.967)	0.226 (0.083)	7.500	0.063	5.542 (2.266)	7.50	0.063
*Citrus paradisi* (grapefruit)^g^	9 (175)	9.720 (2.571)	0.667 (0.236)	13.000	0.070	8.710 (2.990)	17.0	0.016
*Citrus reticulata* (mandarin orange)^g^	9 (178)	10.175 (2.097)	–0.037 (0.180)	–1.000	1.000	0.000 (2.373)	0.0	1.000
*Copaifera reticulata* (copaiba) [weak]	9 (172)	36.007 (2.696)	0.349 (0.101)	16.500	0.023	12.817 (3.685)	16.0	0.023
*Cupressus sempervirens* (cypress) [medium]	9 (179)	33.252 (4.723)	0.686 (0.188)	19.500	0.020	20.389 (7.034)	16.5	0.051
*Cymbopogon flexuosus* (lemongrass) [medium]	9 (175)	49.058 (7.097)	0.806 (0.083)	22.500	0.004	42.145 (8.746)	22.5	0.004
*Cymbopogon martini* (palm rose) [medium]	9 (181)	27.072 (3.742)	0.926 (0.074)	22.500	0.004	25.902 (4.321)	22.5	0.004
*Cymbopogon nardus* (citronella) [medium]	9 (182)	32.434 (5.703)	0.735 (0.112)	18.000	0.008	28.090 (5.930)	18.0	0.008
*Eucalyptus citriodora* (lemon-scented gum) [medium]	9 (174)	32.239 (3.688)	0.851 (0.064)	22.500	0.004	27.554 (3.861)	22.5	0.004
*Eucalyptus globulus* (blue gum)^g^	9 (175)	17.922 (3.811)	0.270 (0.243)	6.000	0.375	4.097 (3.576)	6.50	0.313
*Eucalyptus staigeriana* (lemon-scented ironbark) [strong]	9 (184)	64.390 (7.724)	0.813 (0.076)	22.500	0.004	55.527 (9.175)	22.5	0.004
*Eugenia caryophyllus* (clove) [medium]	9 (162)	49.383 (3.755)	0.930 (0.051)	22.500	0.004	45.679 (4.118)	22.5	0.004
*Foeniculum vulgare* (fennel) [weak]	9 (179)	20.160 (2.281)	0.711 (0.103)	22.500	0.004	13.541 (1.986)	22.5	0.004
*Juniperus communis* (juniper) [medium]	9 (179)	21.180 (4.855)	0.944 (0.056)	22.500	0.004	20.069 (5.013)	22.5	0.004
*Lavandula angustifolia* (English lavender) [medium]	9 (177)	51.148 (6.898)	0.912 (0.059)	22.500	0.004	46.470 (7.128)	22.5	0.004
*Lavandula hybrida* (lavandin) [medium]	9 (179)	46.201 (9.549)	0.717 (0.162)	21.000	0.012	37.066 (9.951)	21.0	0.012
*Litsea cubeba* (mountain pepper) [strong]	9 (177)	72.222 (3.667)	0.827 (0.061)	22.500	0.004	59.766 (5.612)	22.5	0.004
*Melaleuca alternifolia* (tea tree) [strong]	9 (178)	71.534 (2.269)	0.809 (0.058)	22.500	0.004	58.095 (4.830)	22.5	0.004
*Mentha piperita* (peppermint) [strong]	9 (180)	60.752 (6.489)	0.868 (0.053)	22.500	0.004	52.916 (6.955)	22.5	0.004
*Ocimum basilicum* (basil) [strong]	9 (181)	69.558 (3.727)	0.827 (0.058)	22.500	0.004	57.187 (4.351)	22.5	0.004
*Origanum majorana* (marjoram) [strong]	9 (175)	73.254 (4.156)	0.861 (0.032)	22.500	0.004	63.076 (4.306)	22.5	0.004
*Pelargonium graveolens* (geranium) [weak]	9 (180)	20.934 (4.002)	0.919 (0.054)	22.500	0.004	18.765 (3.847)	22.5	0.004
*Pogostemon cablin* (patchouli) [weak]	9 (180)	17.646 (5.257)	0.430 (0.133)	10.500	0.031	9.921 (3.422)	10.5	0.031
*Rosmarinus officinalis* (rosemary) [strong]	9 (183)	66.507 (7.112)	0.866 (0.089)	22.500	0.004	60.530 (8.582)	22.5	0.004
*Salvia sclarea* (sage) [medium]	9 (178)	46.955 (3.795)	0.831 (0.047)	22.500	0.004	38.967 (3.665)	22.5	0.004
*Vetiveria zizanioides* (vetiver) [weak]	9 (186)	17.888 (3.270)	0.926 (0.074)	22.500	0.004	16.777 (3.568)	22.5	0.004
*Zingiber officinale* (ginger)^g^	9 (168)	11.384 (2.551)	0.511 (0.167)	7.500	0.063	6.634 (2.388)	7.50	0.063
linalool^f^	9 (174)	41.631 (7.164)	0.874 (0.059)	22.500	0.004	36.881 (7.128)	22.5	0.04

a N°: number of replicates (number of mosquitoes).

b SE = standard error.

c SAI: spatial activity index.

d SR: signed-rank statistic derived using PROC UNIVARIATE (SAS 2018).

e wSAI: SAI value corrected by the percentage of responding.

f This was used to validate the HITSS experiment at 10% (v/v). *P* > S: statistical significance of results using the nonparametric signed rank analysis. *P* < 0.05 means statistically significant. Chromatographic profiles available in the Supporting Information. Repellency: strong (wSAI >50); medium (20 < wSAI <50); weak (wSAI <20).

g Not significant, *P* > 0.05.

The most repellent oils were *O. majorana* (wSAI 63.076), *R. officinalis* (wSAI 60.530), *L. cubeba* (wSAI 59.766), *C. aurantium amara* (wSAI 58.464), *M. alternifolia* (wSAI 58.095), *O. basilicum* (wSAI 57.187), *E. staigeriana* (wSAI 55.527), *M. piperita* (wSAI 52.916), and *C. aurantium bergamia* (wSAI 50.324). The oils with medium repellency were *A. nobilis, C. sempervirens, C. flexuosus, C. martini, C. nardus, E. citriodora, E. caryophyllus, J. communis, L. angustifolia, L. hybrida*, and *S. sclarea*, with wSAI values ranging from 20.069 to 46.470. The oils with weak repellency, wSAI values between 8.541 and 19.209, were *B. carteri, C. odorata, C. atlantica, C. aurantium, C. aurantium dulcis, Copaifera reticulata, F. vulgare, P. graveolens, P. cablin*, and *V. zizanioides*.

Of the compounds tested (Table 3), citronellol (wSAI 35.490) and menthol (wSAI 34.444) had wSAI values similar to 1% transfluthrin (reported wSAI: 30–40^15^) and were therefore considered strong repellents. Borneol (wSAI 18.333), carvacrol (wSAI 15.088), geraniol (wSAI 13.395), and nerol (wSAI 18.333) exhibited medium repellency. Eugenol (wSAI 2.310) demonstrated weak repellency. Citral (wSAI 27.115), with medium repellency, is a mixture of 32% neral and 63% geranial (Figure S40). Sabinene is a mixture of 85% sabinene and 13% β-pinene (Figure S47). The latter is reported in the literature for its attractive effects on mosquitoes.^28^

**Table 3:** Spatial Repellency Activity of the Volatile Compounds against *Aedes aegypti* Females (Dose 1% v/v or m/v)

Compound [repellency levels]	^a^N°	Mean % responding (±SE^b^)	Mean SAI^c^ (±SE^b^)	SR^d^ SAI^c^	*P* > S SAI^c^	wSAI^e^ (±SE^b^)	SR^d^ wSAI^e^	*P* > S wSAI^e^
borneol [medium]	9 (180)	19.444 (2.561)	0.944 (0.056)	22.5	0.0039	18.333 (2.764)	22.5	0.0039
carvacrol [medium]	9 (179)	15.088 (2.637)	1.000 (0.001)	22.5	0.0039	15.088 (2.637)	22.5	0.0039
1, 8-cineole	9 (177)	21.387 (5.109)	–0.283 (0.209)	–5.5	0.3125	–5.526 (2.952)	–7.5	0.1563
citral [medium]	9 (173)	38.349 (6.167)	0.731 (0.098)	22.5	0.0039	27.115 (4.849)	22.5	0.0039
citronellal	9 (180)	25.556 (4.285)	0.176 (0.142)	5.0	0.4844	6.667 (3.908)	9.0	0.1875
citronellol [strong]	9 (172)	37.712 (6.439)	0.944 (0.056)	22.5	0.0039	35.490 (6.718)	22.5	0.0039
eugenol [weak]	9 (179)	15.701 (3.330)	0.770 (0.125)	18.0	0.0078	2.310 (3.556)	18.0	0.0078
geraniol [medium]	9 (178)	26.728 (5.105)	0.440 (0.1523)	12.0	0.0469	13.395 (5.124)	12.5	0.0469
menthol [strong]	9 (180)	42.222 (6.461)	0.679 (0.178)	20.0	0.0156	34.444 (9.554)	20.0	0.0156
nerol [medium]	9 (180)	23.889 (3.611)	0.746 (0.107)	18.0	0.0078	18.333 (3.436)	18.0	0.0078
sabinene	9 (180)	7.222 (3.017)	–0.352 (0.168)	–13.0	0.0938	–5.000 (2.635)	–12.0	0.1250
transfluthrin^f^	9 (175)	17.173 (2.267)	1.000 (0.001)	22.5	0.0039	17.173 (2.267)	22.5	0.0039

a N°: number of replicates (N° mosquitoes).

b SE: standard error.

c SAI: spatial activity index.

d SR: signed-rank statistic derived using PROC UNIVARIATE (SAS 2018).

e wSAI: SAI value corrected by the percentage of responding.

f Transfluthrin at 0.01% used to validate the HITSS experiment. *P* > S: Statistical significance of results using the nonparametric signed rank analysis. *P* < 0.05: statistically significant. Purity of the compounds ranged from 95 to 98%, with the exception of: Citral (37% neral/63% geranial) and Sabinene (85% sabinene/13% β-pinene). Chromatographic profiles available in the Supporting Information. Repellency: strong (wSAI >30); medium (10 < wSAI <30); and weak (wSAI <10).

The majority of the essential oils tested displayed low/no toxicity, as evidenced by low/no knockdown (KD) rates. *O. majorana* caused 56% KD in the 9 *Ae. aegypti* females that migrated to the HITSS treatment chamber. *Litsea cubeba* caused 5 KD/11 mosquito (45%) treatment side. *Citrus aurantium amara* promoted 5 KD/6 (83%) treatment chamber. The highest KD was caused by *M. alternifolia* essential oil, 11/12 (92%) in the treatment chamber. *Ocimum basilicum* had caused 9 KD from/11 (82%) knockdown. The test involving *E. staigeriana* oil caused 4 KD, with mosquitoes, 2/8 (25%) occurring in the treatment chamber, with a similar effect observed for the *M. piperita* oil test, with a total of 5 KD and 3/7 (43%) occurring in the treatment chamber. Regarding the volatile compounds tested at 1%, with the exception of citral (KD 41%) and eugenol (KD 50%), low/zero knockdown values (0 to 8%) were observed for mosquitoes that migrated to the treatment chamber (Table 3).

## 4 Discussion

The use of essential oils as repellents for personal protection is well-documented, with the literature reaffirming their potential against mosquitoes.^28^ However, few studies have evaluated the spatial repellent activity of these natural products, mainly focusing on topical repellency effects.^28–30^ Our study reports the spatial repellent activity of 36 commercially available essential oils and 11 volatile compounds using the high-throughput screening system (HITSS). The results showed a high percentage of the oils (83%) induced mosquito avoidance of a treated surface due to the presence of air-borne chemicals. In other words, the mosquitoes moved away from the chemical stimulus without touching a treated surface.

The essential oils of *Citrus aurantium* and *C. aurantium dulcis* caused low repellency, while *C. limon, C. paradisi*, and *Citrus reticulata* proved inactive during HITSS testing. These *Citrus* species oils possessed a high limonene content (63 to 99%, Table 1). Both (*S*)-(−)-limonene (wSAI 9.514) and (*R*)-(+)-limonene (wSAI 4.417) demonstrated weak repellency at 1%.^28^ On the other hand, *C. aurantium amara* (wSAI 58.464) and *C. aurantium bergamia* (wSAI 50.324) samples contained linalool (19–28%) and linalyl acetate (40–61%) and acted as strong spatial repellents.

Tested at 1%, citral (32% neral/63% geranial; wSAI 27.115) and nerol (wSAI 18.333) demonstrated medium repellency (Table 3). Therefore, the presence of α-terpinolene (10%), nerol (2%), neral (15%), and geranial (21%) in *E. staigeriana* oil justified its activity (wSAI 55.527). Isopulegol (6%), citronellal (83%), and citronellol (10%) are present in *E. citriodora* oil (wSAI 27.554), which is determined as a medium repellent. Citronellol on its own, at 1% (wSAI 35.490, Table 3), was very active and may have been the most relevant constituent of the *E. citriodora* oil regarding repellency. The compound 1, 8-cineole at 1% (SAI −0.283, wSAI −5.526, Table 3) showed an attractant activity, corresponding with its elevated presence (93%) in *E. globulus* oil.

Both 1, 8-cineole (Table 3) and (−)-β-pinene (SAI −0.504, wSAI −17.445)^28^ are attractant constituents, while *p*-cymene (SAI 0.246, wSAI 13.191)^28^ and borneol (SAI 0.944, wSAI 18.333, Table 3) have medium repellency. *Rosmarinus officinalis* oil composition included: α-pinene (26%), camphene (9%), β-pinene (6%), myrcene (2%), *p*-cymene (3%) 1, 8-cineole (21%), camphor (26%), borneol (3%), and α-terpineol (3%). The attractant compounds β-pinene^28^ and 1, 8-cineole collectively account for 27% of the oil composition. However, this amount was insufficient to overcome the repellency compounds combined, involving α-pinene, camphene, *p*-cymene,^28^ camphor, borneol, and α-terpineol, corresponding to 70% of the oil composition, promoting *R. officinalis* oil with wSAI 60.530.

Similar to *R. officinalis*, *L. cubeba* oil (wSAI 59.766) contains 5% β-pinene and 18% 1, 8-cineole. Citral, a medium repellent (wSAI 27.115, Table 3), is a 32% neral/63% geranial mixture. In *L. cubeba* oil, both neral (26%) and geranial (37%) contributed to the repellency. Menthol at 1% (wSAI 34.444, Table 3) was a strong repellent. In the *M. piperita* oil, besides menthol (49%), there is also menthone (26%) and α-terpineol (9%), supporting its wSAI 52.916. The sum of linalool (21%) and *p*-allylanisole (75%) corresponded to 96% of *O. basilicum* oil composition, which had a wSAI of 57.187. *O. majorana* oil is composed of 2 repellent constituents, *p*-cymene (12%) and linalool (29%), in addition to terpinen-4-ol (35%), justifying its strong repellency (wSAI 63.076).

In assessing the medium repellent samples, *C. flexuosus* oil (wSAI 42.145) was stronger than the *C. nardus* (wSAI 28.090) and *C. martini* (wSAI 25.902) oils, probably due to neral (31%) and geranial (49%) presence in the *C. flexuosus* oil (80%). Although eugenol was considered a weak repellent (wSAI 2.310, Table 3), its high content in *E. caryophyllus* oil (85%), resulted in a wSAI of 45.679. The repellent activity of the *L. angustifolia* (wSAI 46.470) and *L. hybrida* (wSAI 37.066) oils was associated with linalool (37%), linalyl acetate (62%), and myrtanol (34%) content. Significant contents of α-pinene, ranging from 26 to 67%, were observed in the *B. carteri*, *C. sempervirens*, *J. communis,* and *R. officinalis* oils, accounting for their classification as medium to strong repellents.

The essential oils were tested at a 10% dose to evaluate their effects on *Ae. aegypti* female behavior. This concentration was defined based on previous studies with natural products.^15,29,30^ *O. majorana*, *R. officinalis*, and *L. cubeba* essential oils showed spatial repellent activity (wSAI >50) and low toxicity to mosquitoes. Behavior-based control strategies for *Ae. aegypti*, rather than approaches relying on acute toxicity, may help mitigate the development of resistance.^30^ On the other hand, the *M. alternifolia, C. aurantium amara*, and *O. basilicum* essential oils also exhibited wSAI >50 but were associated with higher toxicity, as indicated by increased knockdown rates.

Our findings determined *O. majorana* oil to be a strong spatial repellent, with the highest wSAI value (63.076). In the literature, we found that *O. majorana* oil presented >60% topical repellency against *Culex pipiens* at 0.005 mg/cm^2^.^31^ It also afforded protection against *Ae. albopictus* at 0.2 mg/cm^2^ following an arm-in-cage methodology.^32^ The *R. officinalis* oil had a wSAI of 60.530 and was also classified as a strong repellent. This oil was previously tested in a modified apparatus^33^ and proved active.^34^ *L. cubeba* was ranked as the third strongest spatial repellent in the present study according to the wSAI (59.766) and was previously associated with repellency against *Ae. albopictus* when tested in a y-tube olfactometer.^35^ *L. cubeba* also presented topical repellency against *Ae. aegypti*.^36^

*M. alternifolia* oil, in which γ-terpinene (23%) and terpinen-4-ol (51%) collectively accounted for 74% of its composition, demonstrated a wSAI of 58.095. *O. basilicum* oil, classified herein as a strong spatial repellent (wSAI 57.187) also showed topical repellency when tested in an arm-in-cage methodology against *Ae. albopictus*.^32^ The oils from *C. aurantium* varieties showed different degrees of spatial repellency in the HITSS: petitgrain (*C. aurantium amara*, wSAI 58.464) and bergamot (*C. aurantium bergamia*, wSAI 50.324) presented strong repellency, while sour orange (*C. aurantium*) and sweet orange (*C*. *aurantium dulcis*) displayed weak repellency. Although mandarin (*C. reticulata*) oil did not promote repellency in the present study, both mandarin and lemon (*C*. *limon*) oils were previously associated with *Ae*. *aegypti* repellency in human volunteer trials which evaluated mosquito landing in the presence of these oils.^37^ Grapefruit (*C. paradisi*) oil also demonstrated repellency in an olfactometer assay with *C. pipiens pallens*.^31^

Menthol (49%) was the major compound in *M. piperita* oil (Table 1) and was determined to be a strong repellent (Table 3). This oil had its topical repellency studied in vivo, where menthol was the major compound conferring bite protection when compared to DEET.^38^ *E. globulus* essential oil was classified as a weak spatial repellent when tested in the HITSS, while *E. staigeriana* and *E. citriodora* exhibited strong and medium repellency, respectively. Interestingly, *E. globulus* essential oil is registered in the United States as a safe and effective topical *Ae. aegypti* repellent.^39^ Both *Lavandula* species demonstrated medium *Ae. aegypti* spatial repellency in our study, however, in a previous study employing different test methodology, *L. angustifolia* oil showed topical repellency against *Ae. albopictus*.^32^ An oil from *L. gibsoni* also demonstrated repellent activity, extending the protection time of treated human hands against *Ae. aegypti* females.^40^ Other essential oils with medium/weak spatial repellency at 10% were reported in the literature as topical repellents against mosquitoes: clove,^41^ sage oil,^31^ junipers,^36^ palm rose,^42,43^ cypresses,^32^ chamomiles,^31^ copaiba,^39^ fennels, patchouli, and ylang ylang.^31^

## 5 Conclusion

Of the 36 commercial essential oils evaluated in this comprehensive study, 20 modified *Ae. aegypti* mosquito behavior and could potentially create vector-free spaces if employed as environmental repellents, either in an individual or combined formulation to develop new spatial repellent strategies. Our study specifically identified: 9 strong spatial repellents (wSAI 50 to 63)—*O. majorana*, *R. officinalis*, *L. cubeba*, *M. alternifolia*, *C. aurantium amara*, *O. basilicum*, *E. staigeriana*, *M. piperita,* and *C. aurantium bergamia*; and 11 medium spatial repellents (wSAI 20 to 46)—*A. nobilis, C. sempervirens, C. flexuosus, C. martini, C. nardus, E. citriodora, E. caryophyllus, J. communis, L. angustifolia, L. hybrida,* and *S. sclarea*. Our HITSS tests evaluated 11 volatile compounds contained in the essential oils with spatial repellent activity individually, adding previously undescribed data. The spatial repellent knowledge acquired for these compounds, together with the identification of 92 chemical structures present in the oils, suggested structure-repellency relationships. Linalool, citronellol, menthol, citral (neral/geranial), borneol, nerol, and carvacrol acted as spatial repellents, while 1, 8-cineole and sabinene (sabinene/β-pinene) behaved as attractants. Future studies should focus on developing formulations involving the most active essential oils, either individually, combined or enriched with isolated constituents, and advance to semifield trials designed to assess spatial repellency as the next important step.

## Supplementary Material



## Notes

GC-FID chromatographic profiles of the 36 commercial essential oil samples and the 11 volatile compounds studied (PDF)
