# Supplementary material for: Essential Oil Composition and Spatial Repellency against Aedes aegypti Using a High-Throughput Screening System
Source: ACS Omega. 2026 Jul 25;11(31):46711–22. doi: 10.1021/acsomega.6c04497 (PMC13470846; doi:10.1021/acsomega.6c04497)
Supplement: Supplementary file 1 [file ao6c04497_si_001.pdf]

Supporting Information

Essential oil composition and spatial repellency against *Aedes aegypti* using a High-Throughput Screening System

João Paulo B. Sousa[a†], Natália M.G. Magalhães[a†], Naira S. Campos[a], Lais S. Morais[a,b], Thiago R.B. Mello[a], Wanessa G. Santiago[a], Ciro Martins Gomes[b], Nicole L. Achee[c], John P. Grieco[c], Lorena C. Albernaz[a,b] and Laila S. Espindola[a,b*]

[a]Laboratório de Farmacognosia, Universidade de Brasília, Campus Universitário Darcy Ribeiro, Brasília, 70910-900, Brazil

[b]Programa de Pós-Graduação em Ciências Médicas, Universidade de Brasília, Campus Universitário Darcy Ribeiro, Brasília, 70910-900, Brazil

[c]Department of Biological Sciences, Eck Institute for Global Health, University of Notre Dame, Notre Dame, IN 46556

[†]Equal contribution.

*Corresponding author: darvenne@unb.br (Espindola LS)

1. GC-FID Chromatographic profiles of the studied essential oil samples (Figures SI1-SI36).

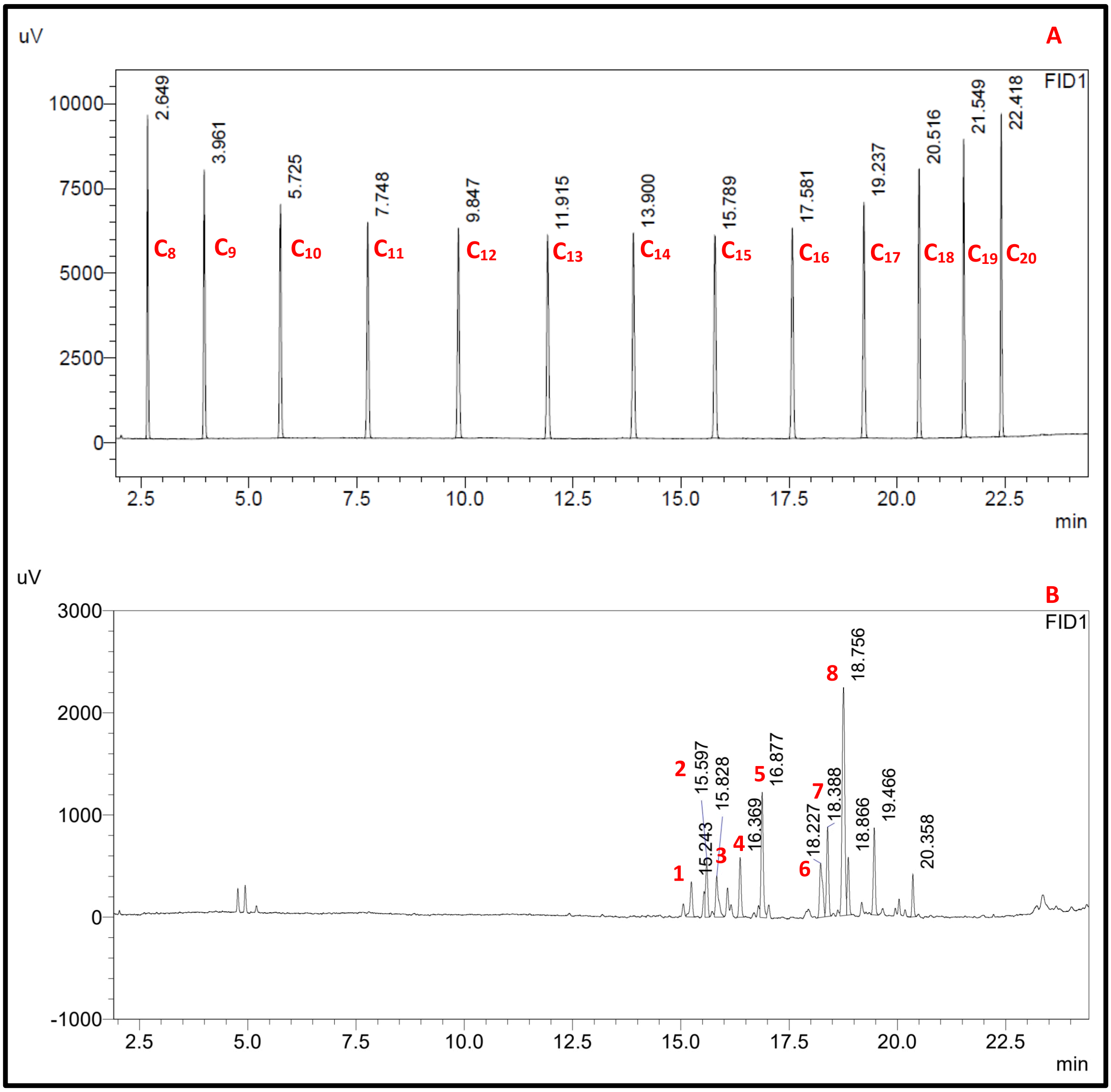

Figure S1: GC-FID Chromatographic profiles. A) Homologous hydrocarbon series, C_8-C_{20}. B) *Amyris balsamifera bark* commercial essential oil. Very minor peaks at five minutes are solvent impurities (hexane). The peak numbering is detailed in Table 1.

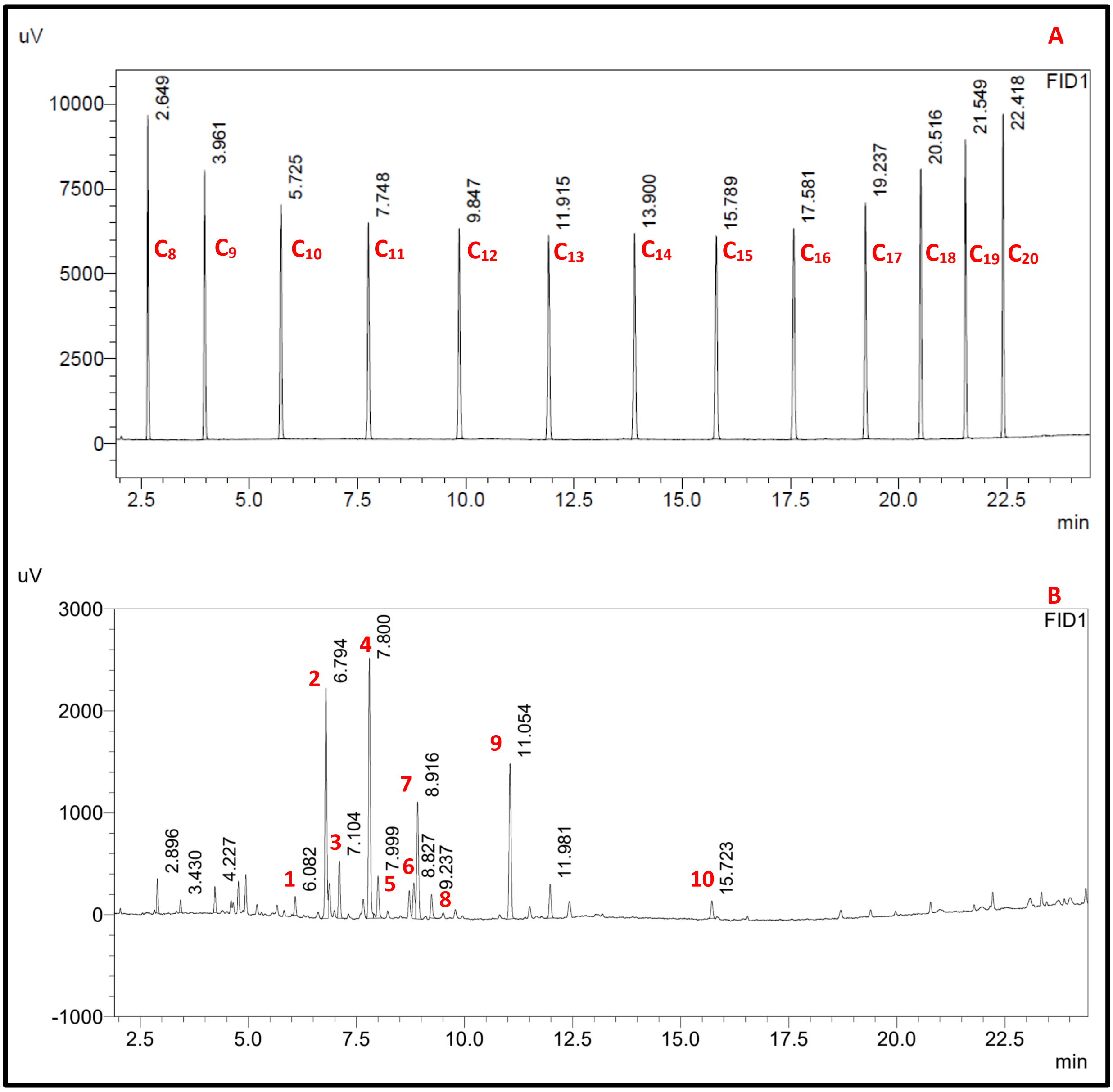

Figure S2: GC-FID Chromatographic profiles. A) Homologous hydrocarbon series, C_8-C_{20}. B) *Anthemis nobilis* commercial essential oil. Very minor peaks at five minutes are solvent impurities (hexane). The peak numbering is detailed in Table 1.

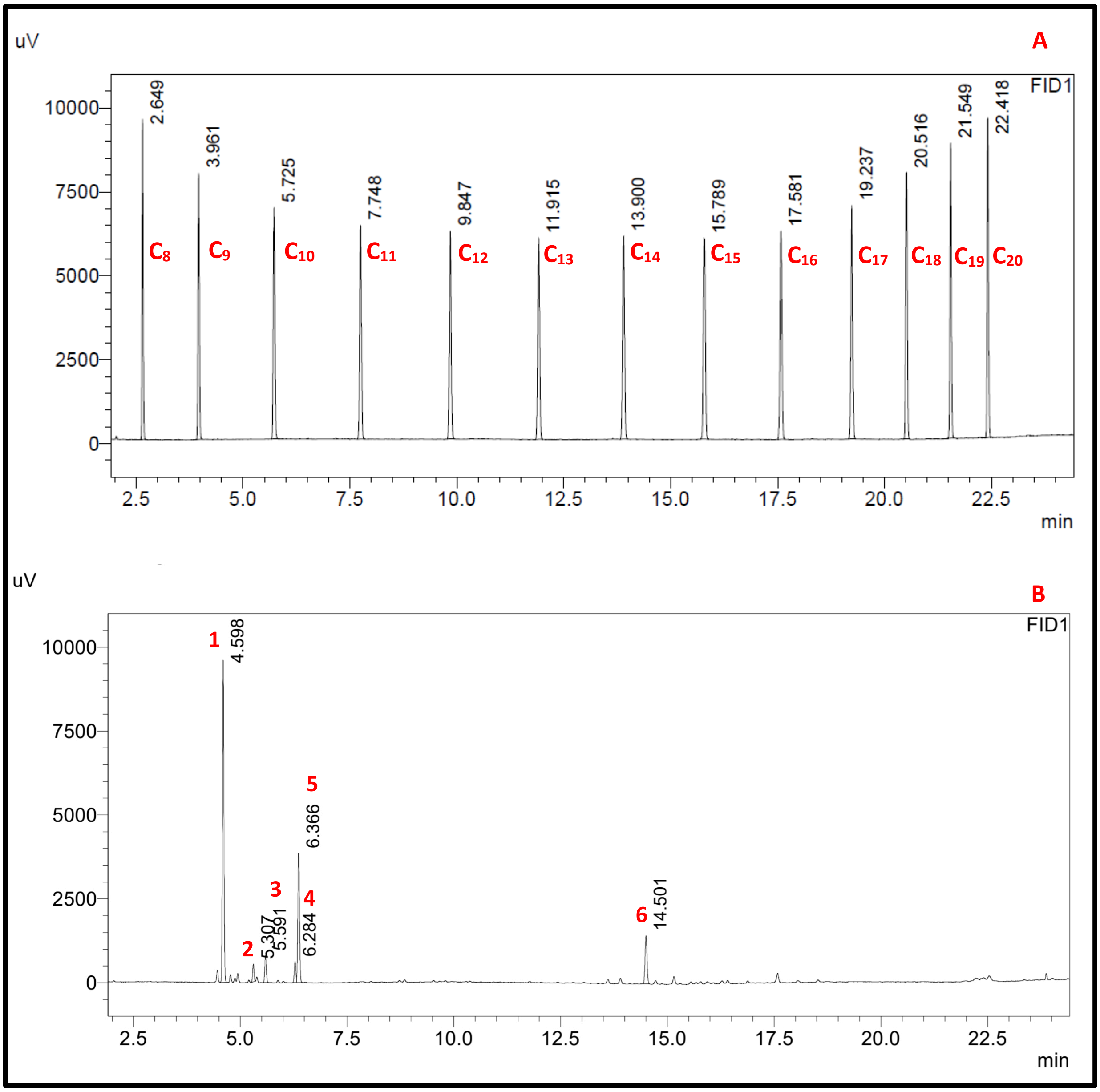

Figure S3: GC-FID Chromatographic profiles. A) Homologous hydrocarbon series, C_8-C_{20}. B) *Boswellia carteri* commercial essential oil. Very minor peaks at five minutes are solvent impurities (hexane). The peak numbering is detailed in Table 1.

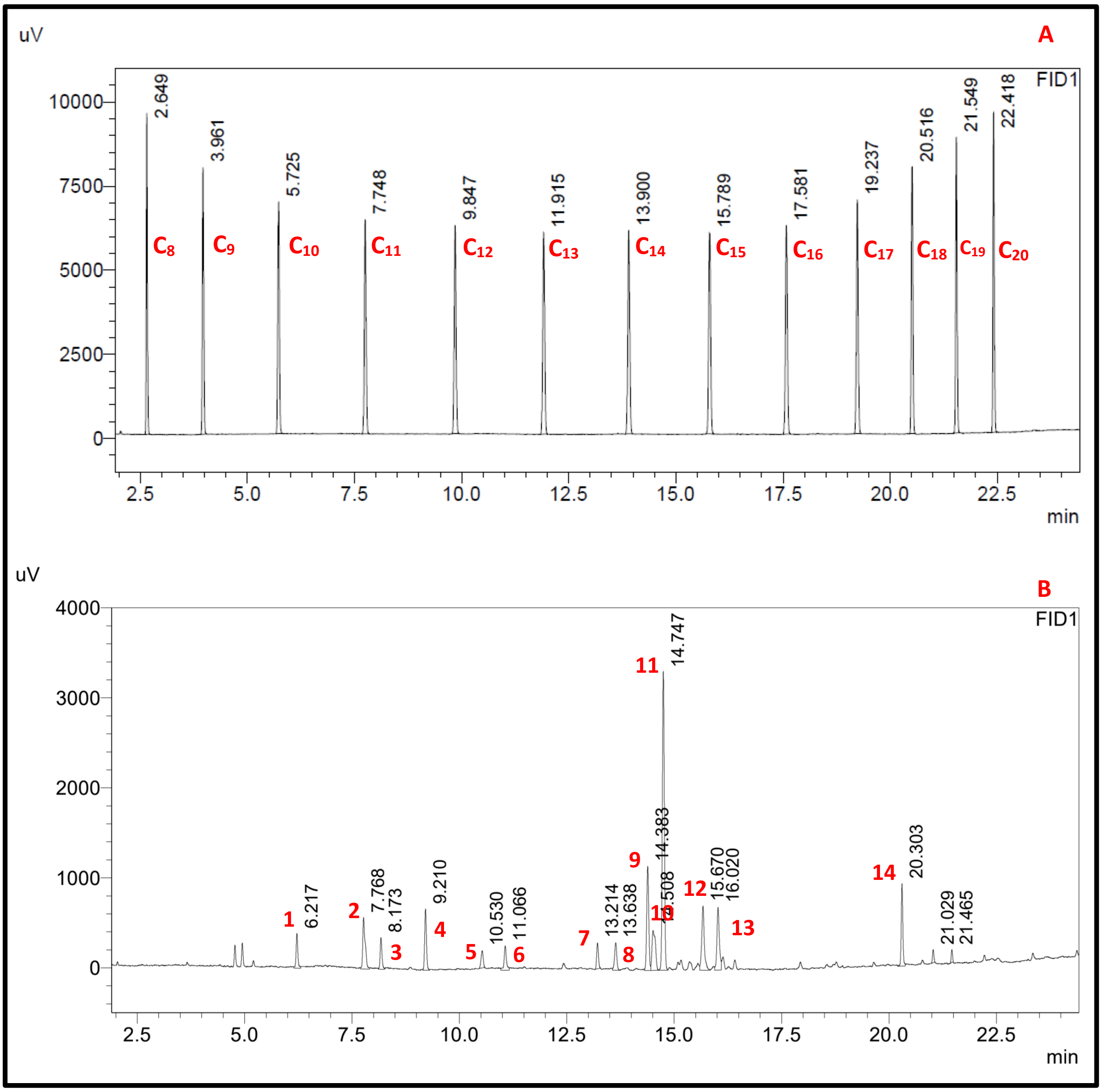

Figure S4: GC-FID Chromatographic profiles. A) Homologous hydrocarbon series, C_8-C_{20}. B) *Cananga adorata* commercial essential oil. Very minor peaks at five minutes are solvent impurities (hexane). The peak numbering is detailed in Table 1.

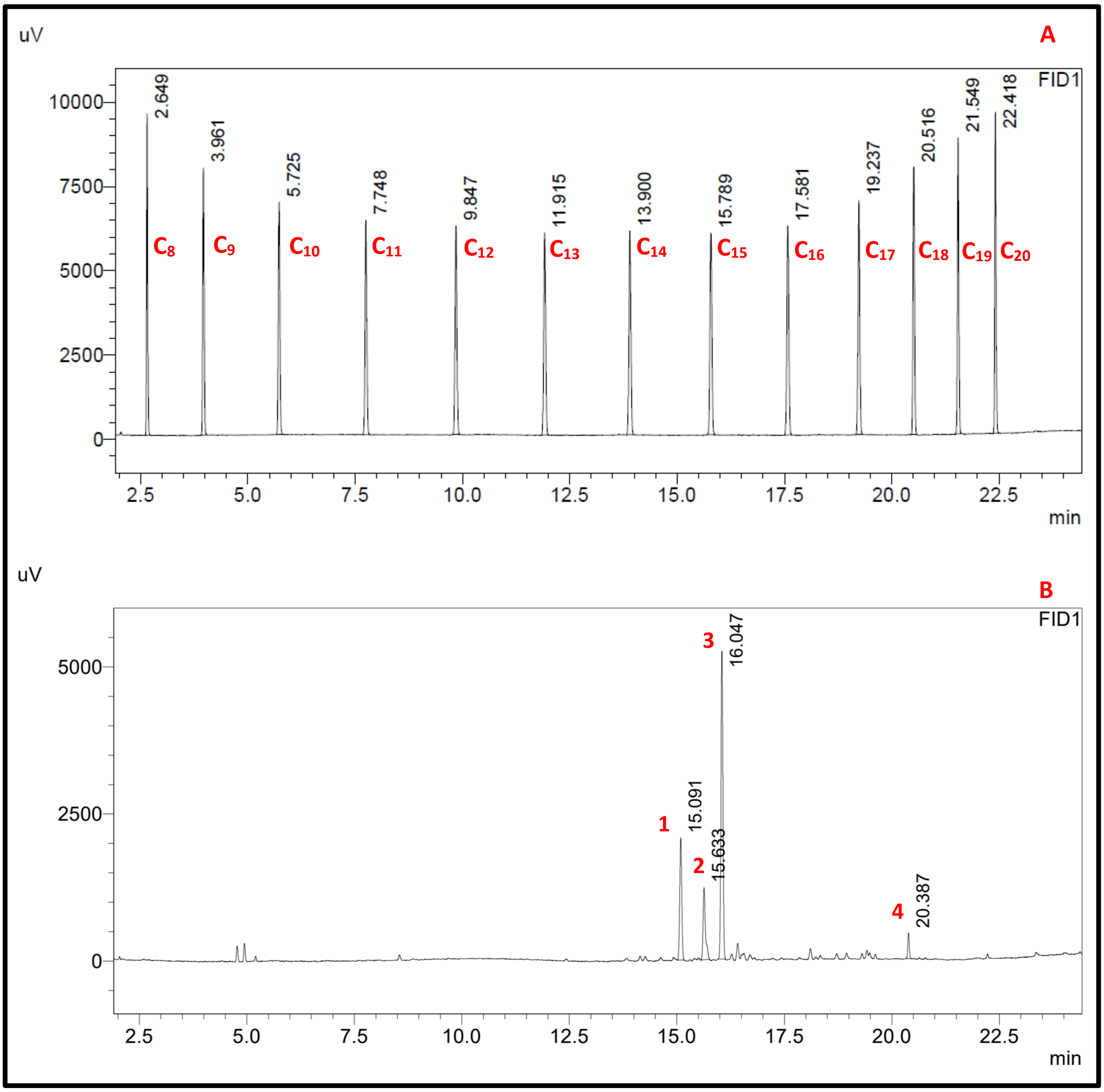

Figure S5: GC-FID Chromatographic profiles. A) Homologous hydrocarbon series, C_8-C_{20}. B) *Cedrus atlantica* commercial essential oil. Very minor peaks at five minutes are solvent impurities (hexane). The peak numbering is detailed in Table 1.

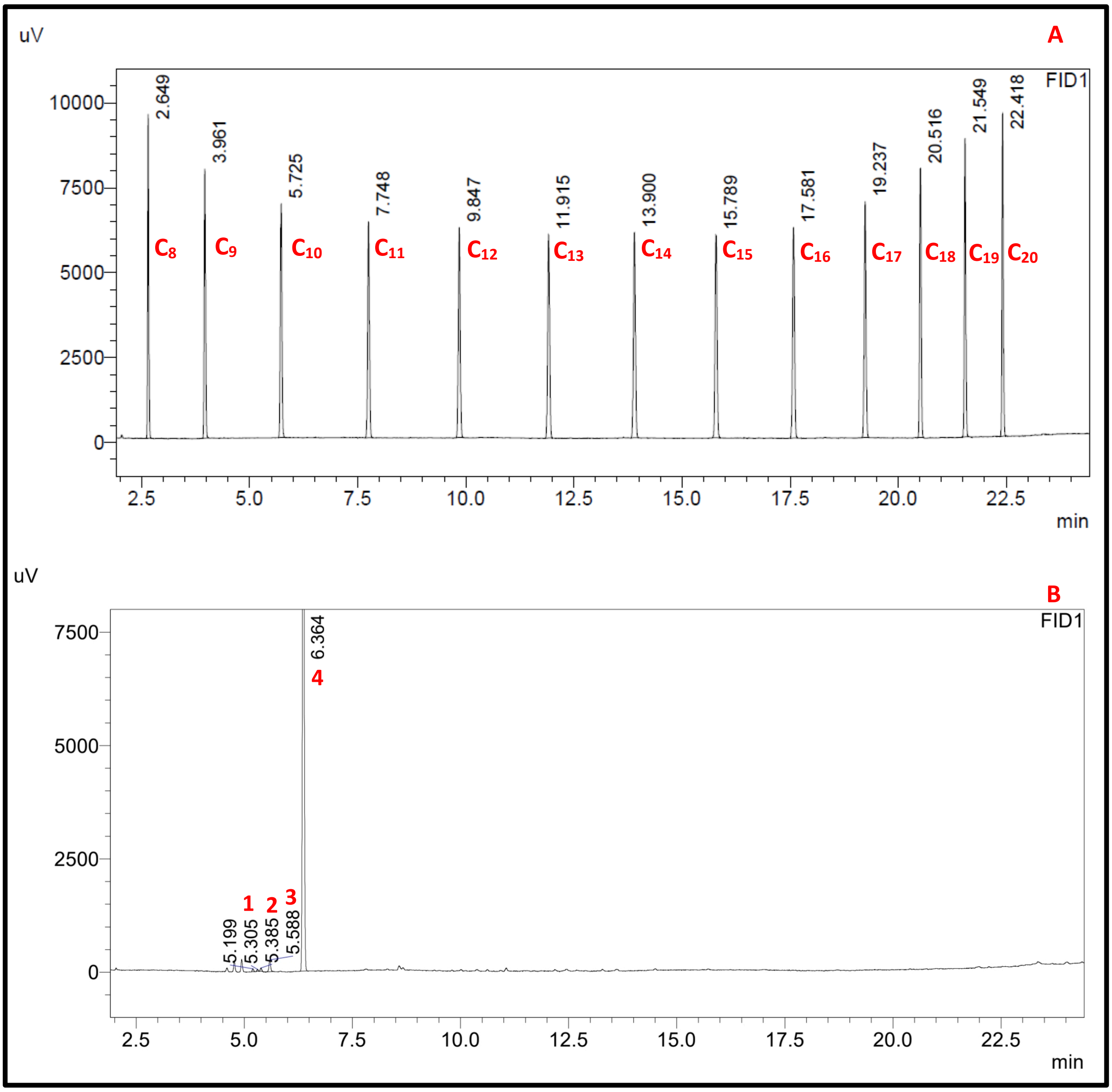

Figure S6: GC-FID Chromatographic profiles. A) Homologous hydrocarbon series, C_8-C_{20}. B) *Citrus aurantium* (sour orange) commercial essential oil. Very minor peaks at five minutes are solvent impurities (hexane). The peak numbering is detailed in Table 1.

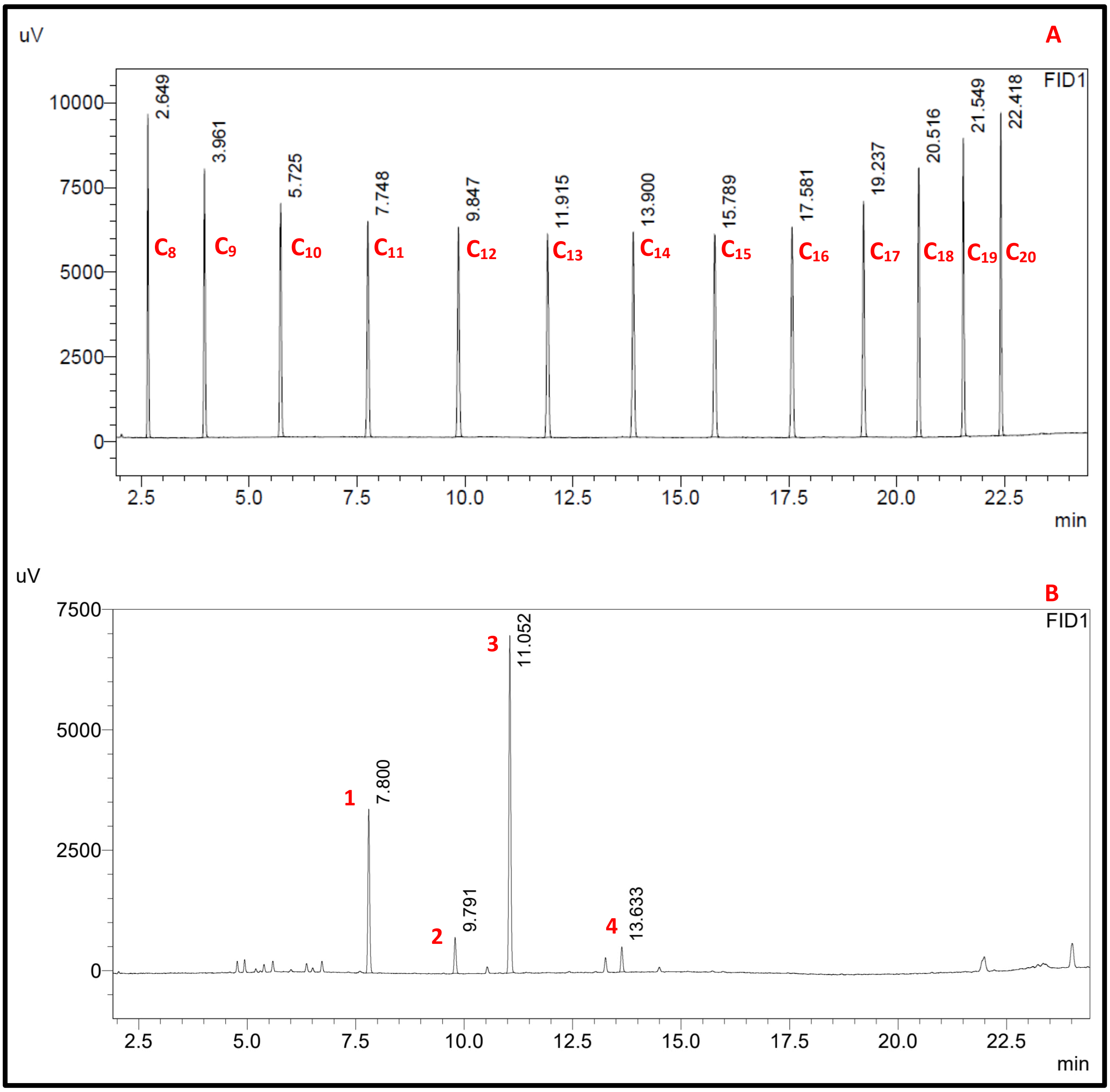

Figure S7: GC-FID Chromatographic profiles. A) Homologous hydrocarbon series, C_8-C_{20}. B) *Citrus aurantium amara* commercial essential oil. Very minor peaks at five minutes are solvent impurities (hexane). The peak numbering is detailed in Table 1.

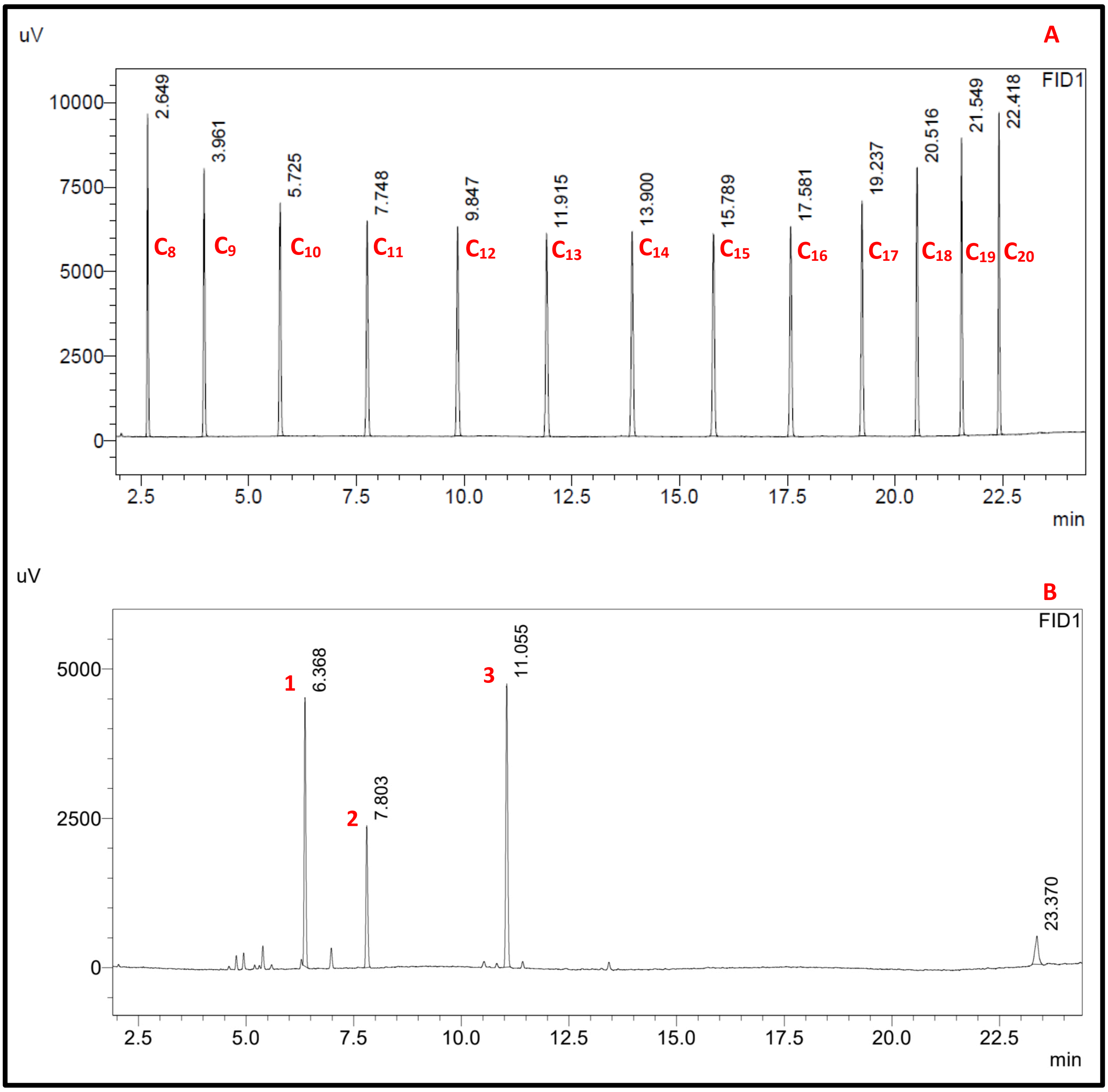

Figure S8: GC-FID Chromatographic profiles. A) Homologous hydrocarbon series, C_8-C_{20}. B) *Citrus aurantium bergamia* commercial essential oil. Very minor peaks at five minutes are solvent impurities (hexane). The peak numbering is detailed in Table 1.

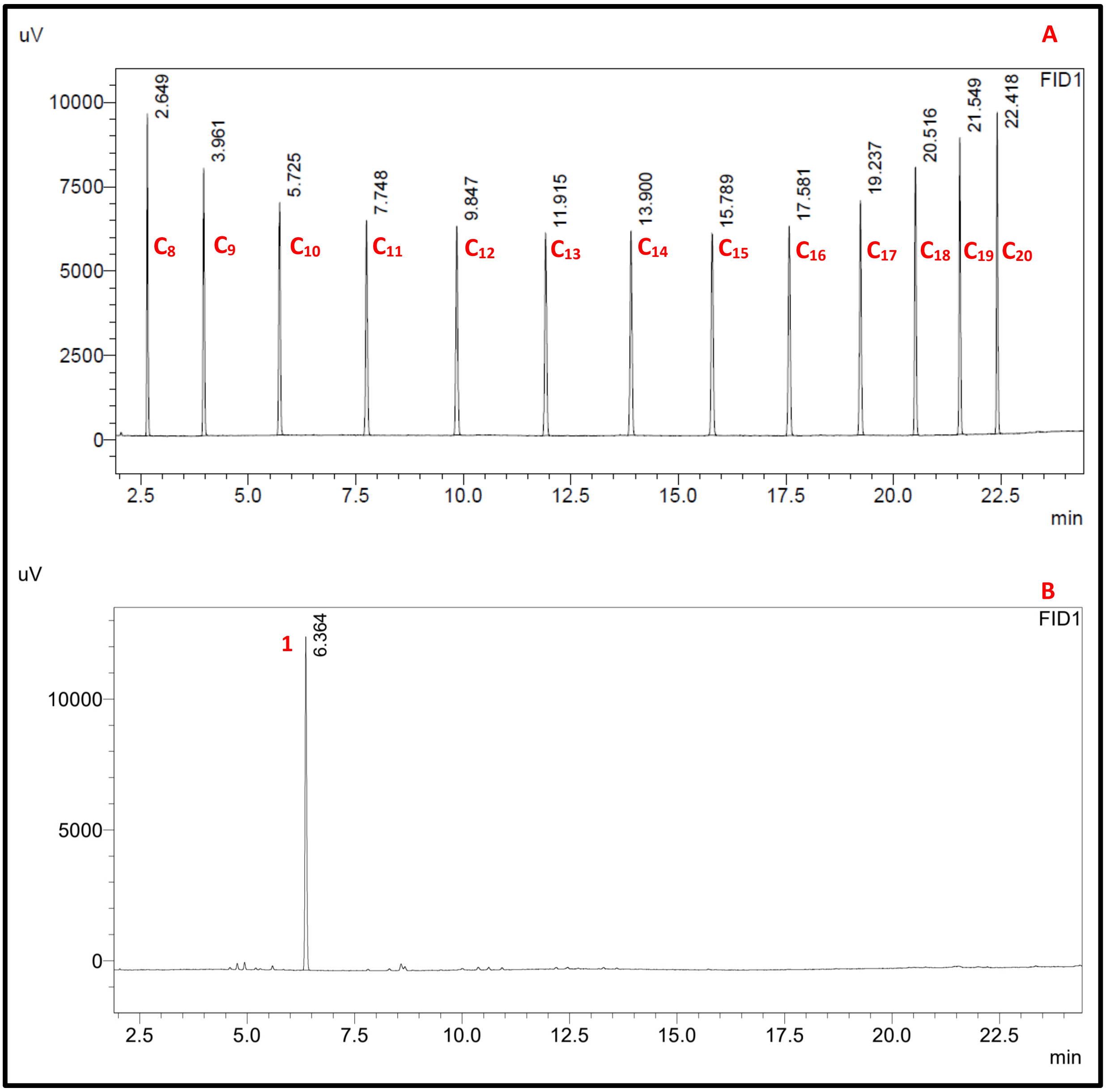

Figure S9: GC-FID Chromatographic profiles. A) Homologous hydrocarbon series, C_8-C_{20}. B) *Citrus aurantium dulcis* commercial essential oil. Very minor peaks at five minutes are solvent impurities (hexane). The peak numbering is detailed in Table 1.

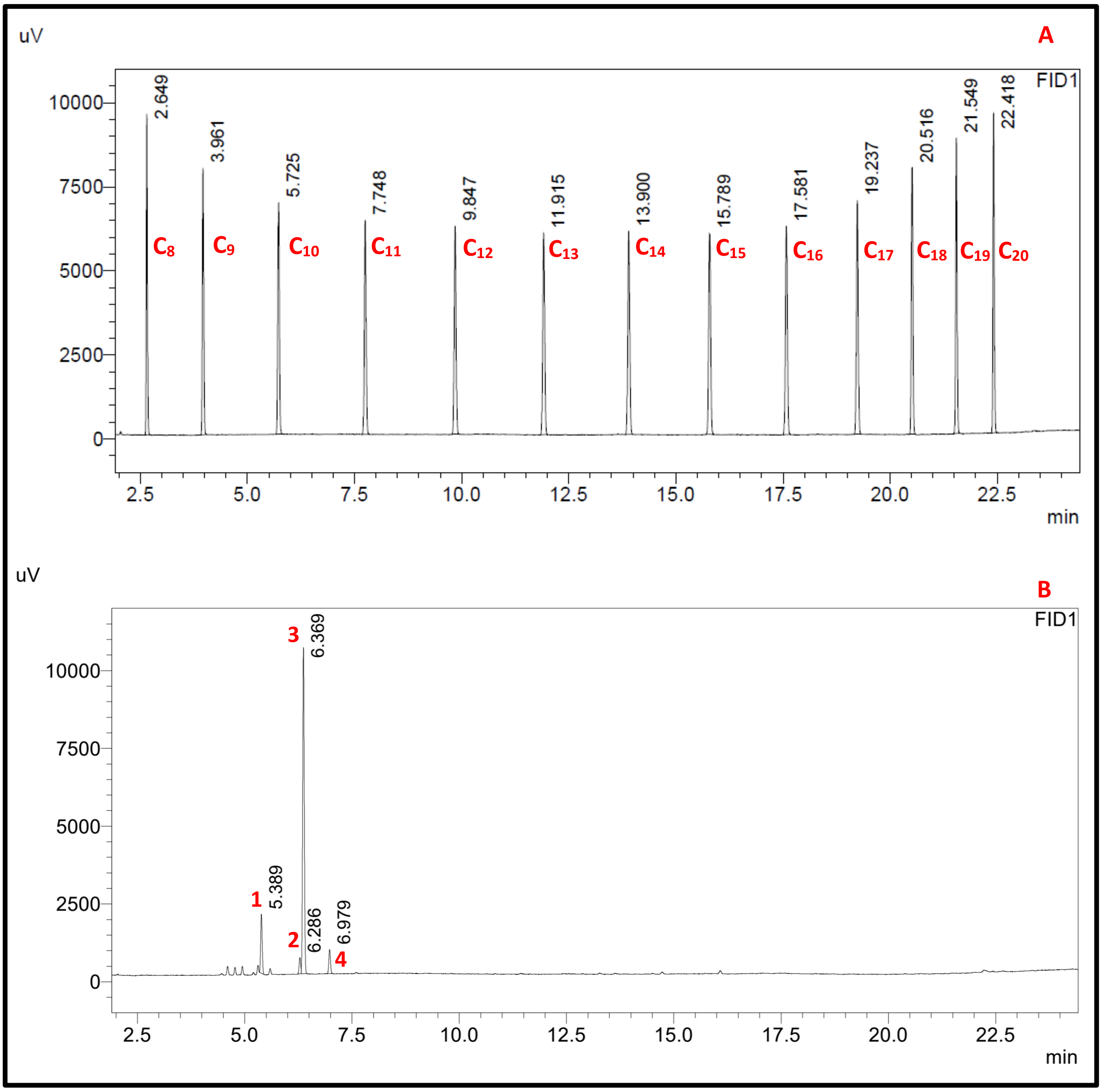

Figure S10: GC-FID Chromatographic profiles. A) Homologous hydrocarbon series, C_8-C_{20}. B) *Citrus limon* commercial essential oil. Very minor peaks at five minutes are solvent impurities (hexane). The peak numbering is detailed in Table 1.

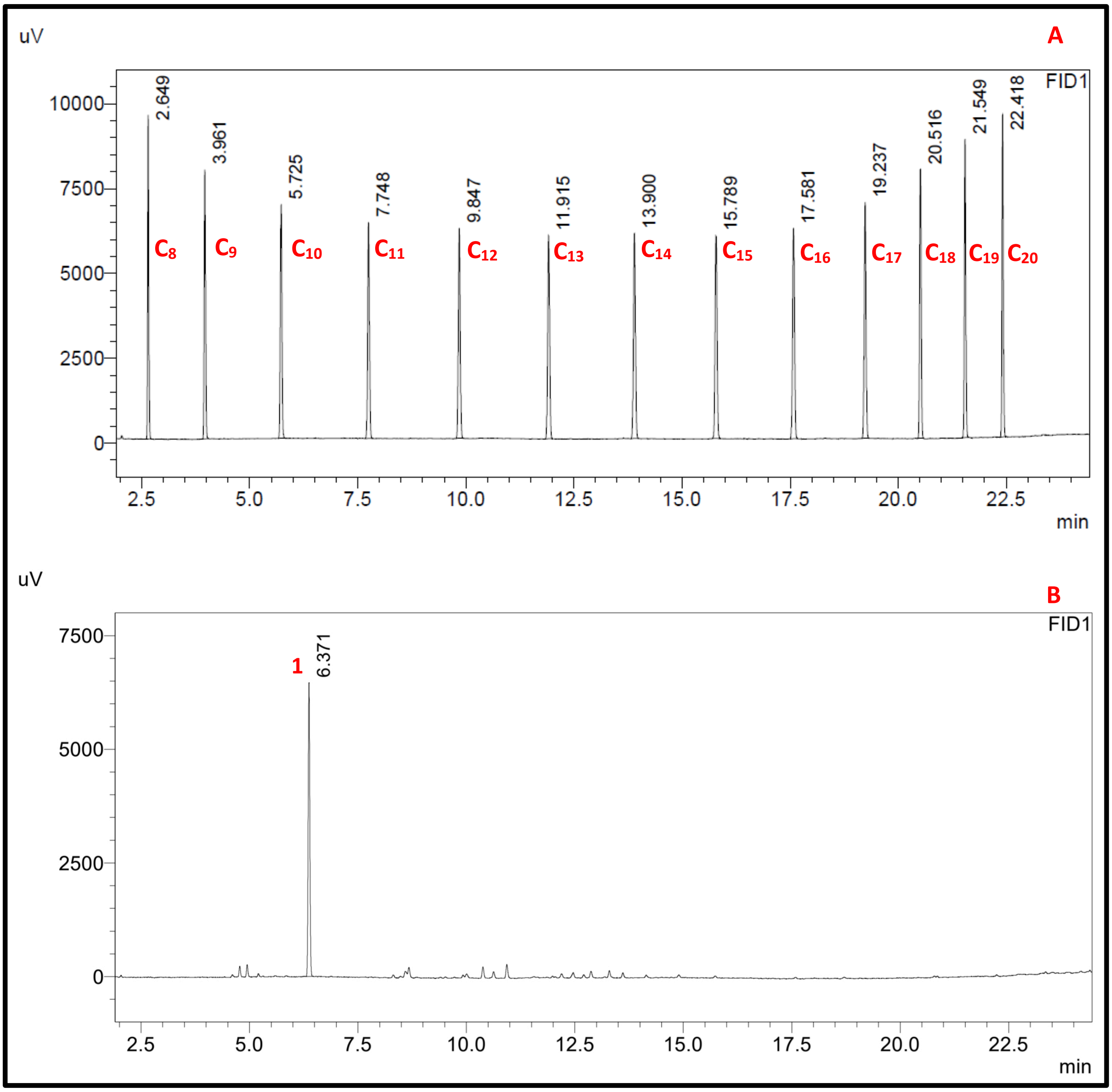

Figure S11: GC-FID Chromatographic profiles. A) Homologous hydrocarbon series, C_8-C_{20}. B) *Citrus paradisi* commercial essential oil. Very minor peaks at five minutes are solvent impurities (hexane). The peak numbering is detailed in Table 1.

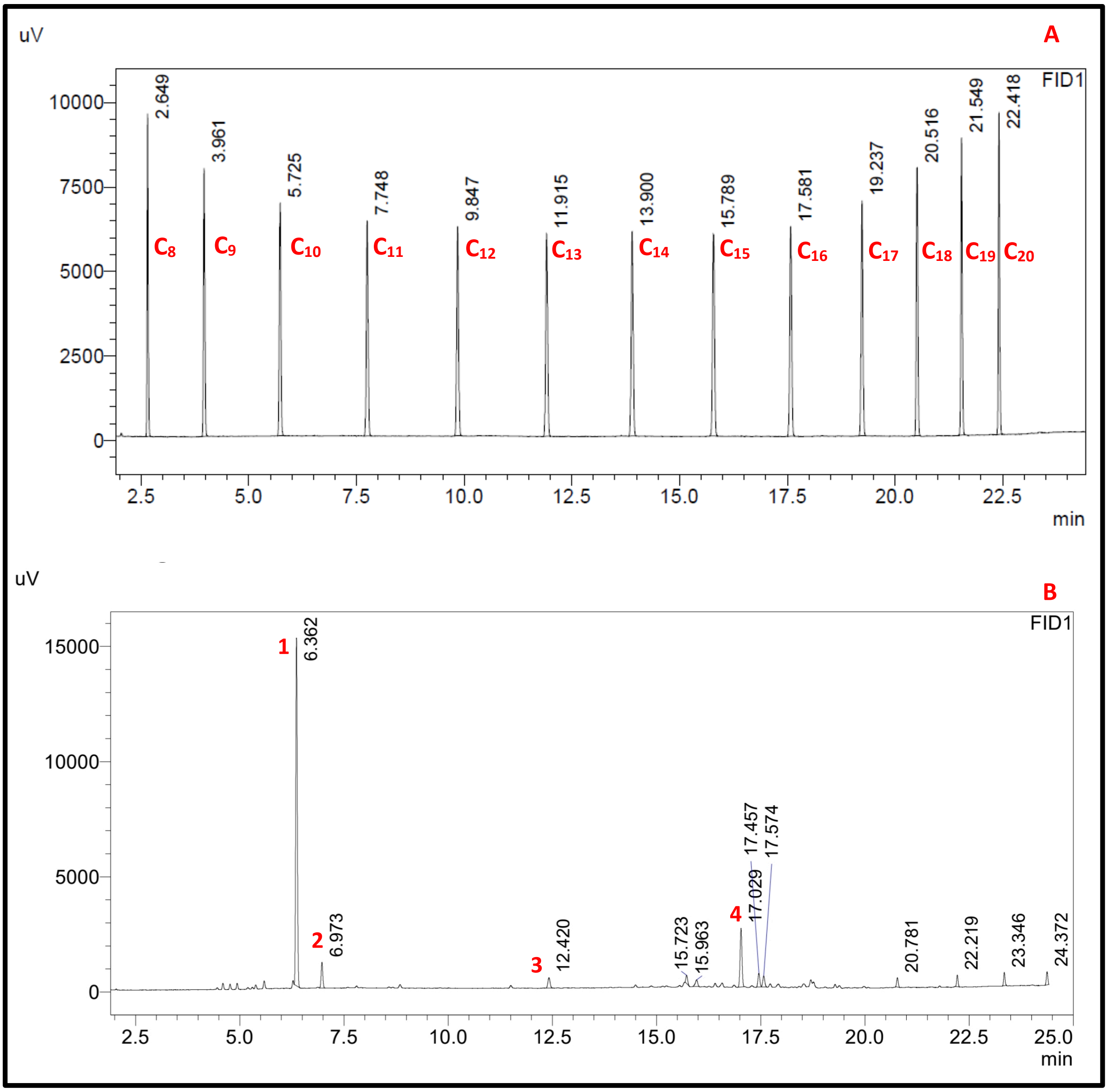

Figure S12: GC-FID Chromatographic profiles. A) Homologous hydrocarbon series, C_8-C_{20}. B) *Citrus reticulata* commercial essential oil. Very minor peaks at five minutes are solvent impurities (hexane). The peak numbering is detailed in Table 1.

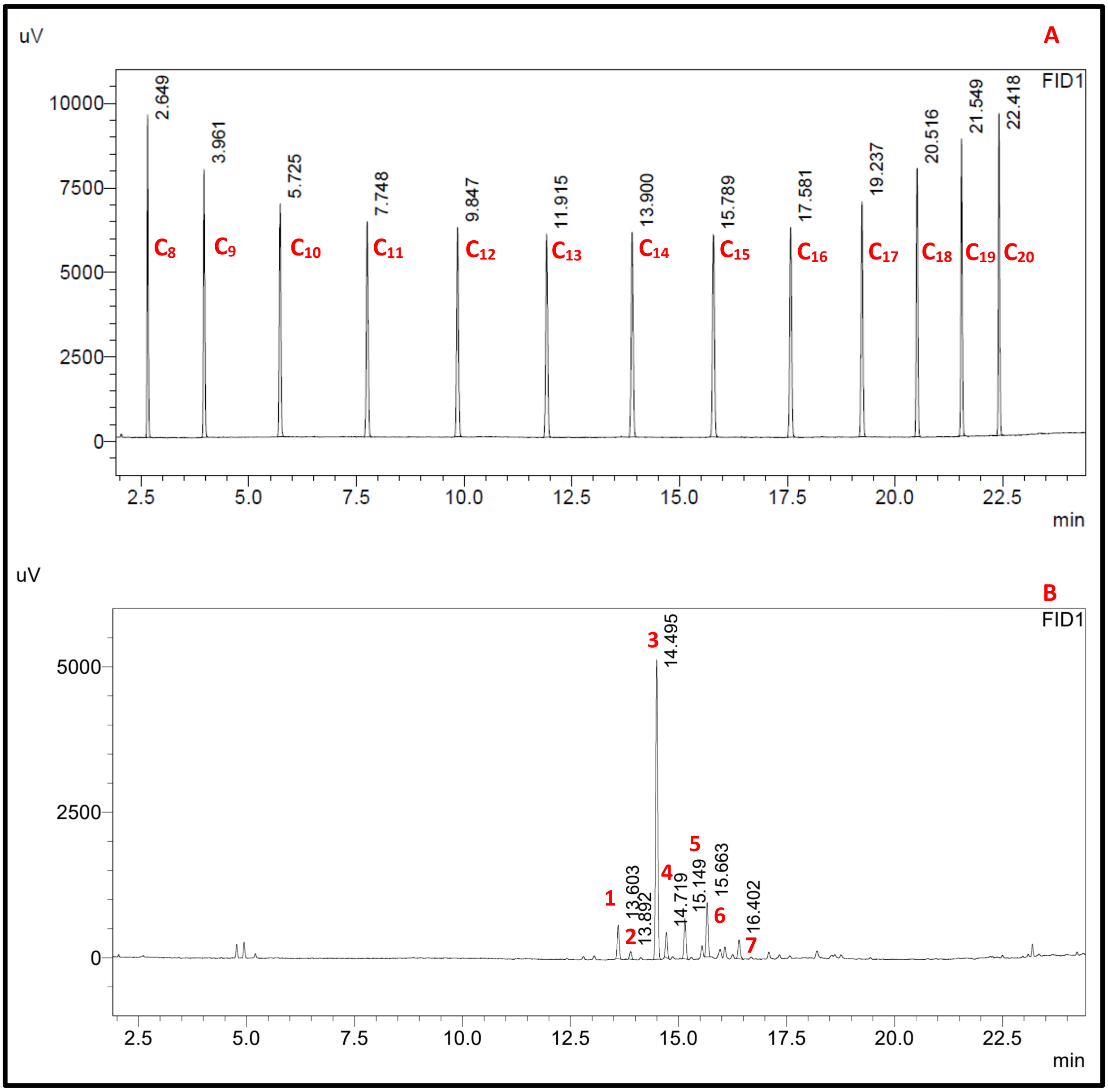

Figure S13: GC-FID Chromatographic profiles. A) Homologous hydrocarbon series, C_8-C_{20}. B) *Copaifera reticulata* commercial essential oil. Very minor peaks at five minutes are solvent impurities (hexane). The peak numbering is detailed in Table 1.

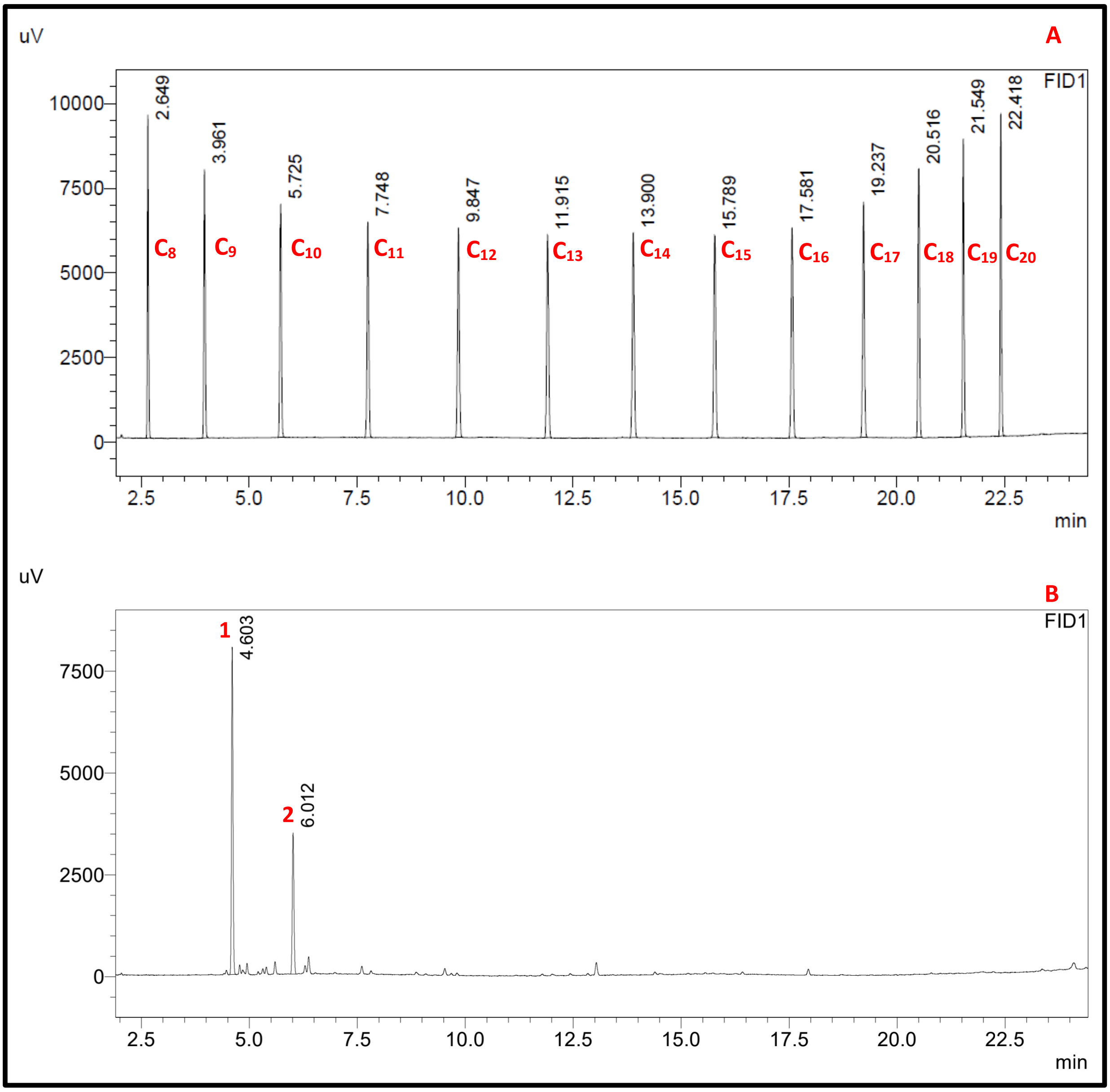

Figure S14: GC-FID Chromatographic profiles. A) Homologous hydrocarbon series, C_8-C_{20}. B) *Cupressus sempervirens* commercial essential oil. Very minor peaks at five minutes are solvent impurities (hexane). The peak numbering is detailed in Table 1.

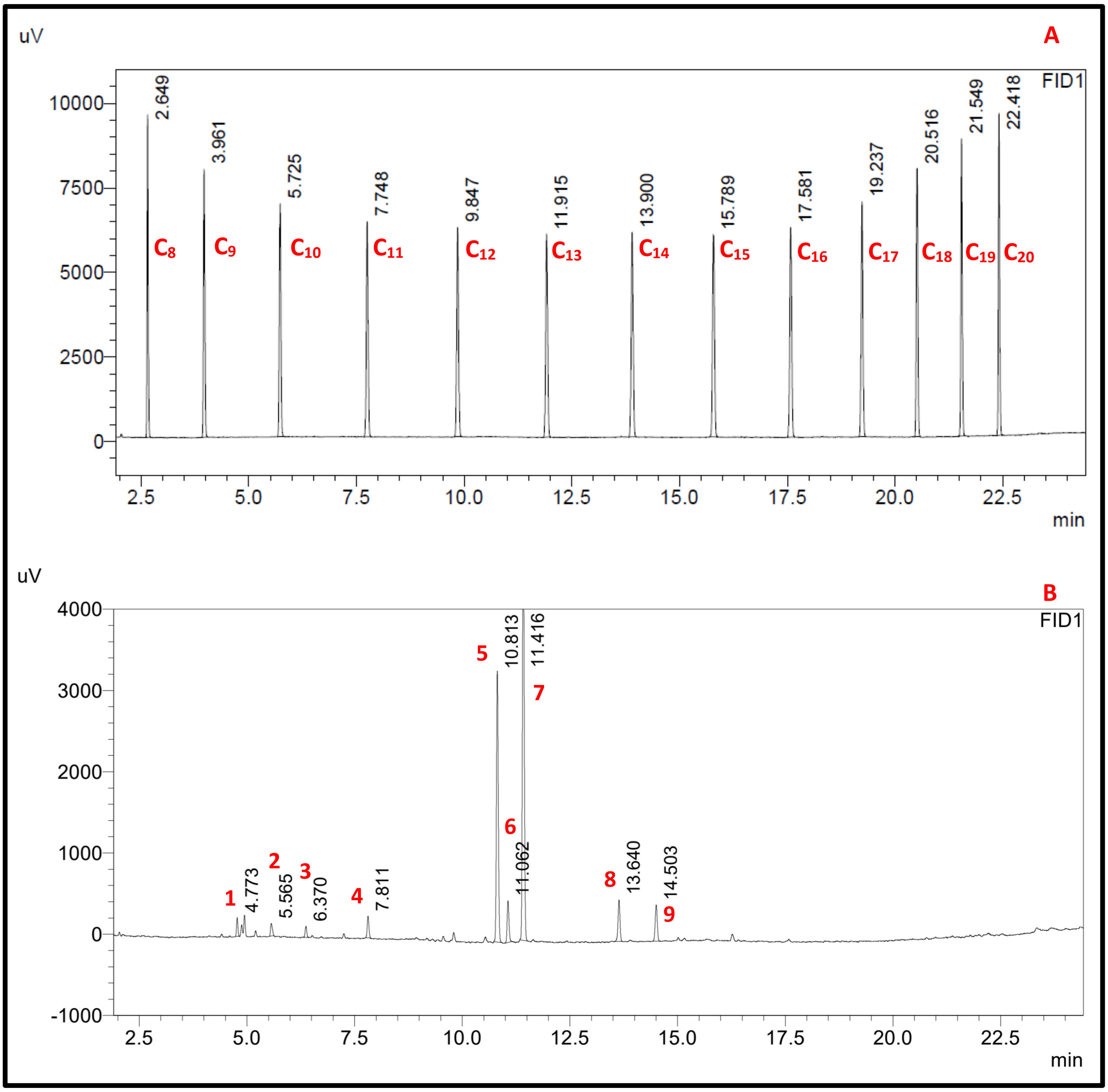

Figure S15: GC-FID Chromatographic profiles. A) Homologous hydrocarbon series, C_8-C_{20}. B) *Cymbopogon flexuosus* commercial essential oil. Very minor peaks at five minutes are solvent impurities (hexane). The peak numbering is detailed in Table 1.

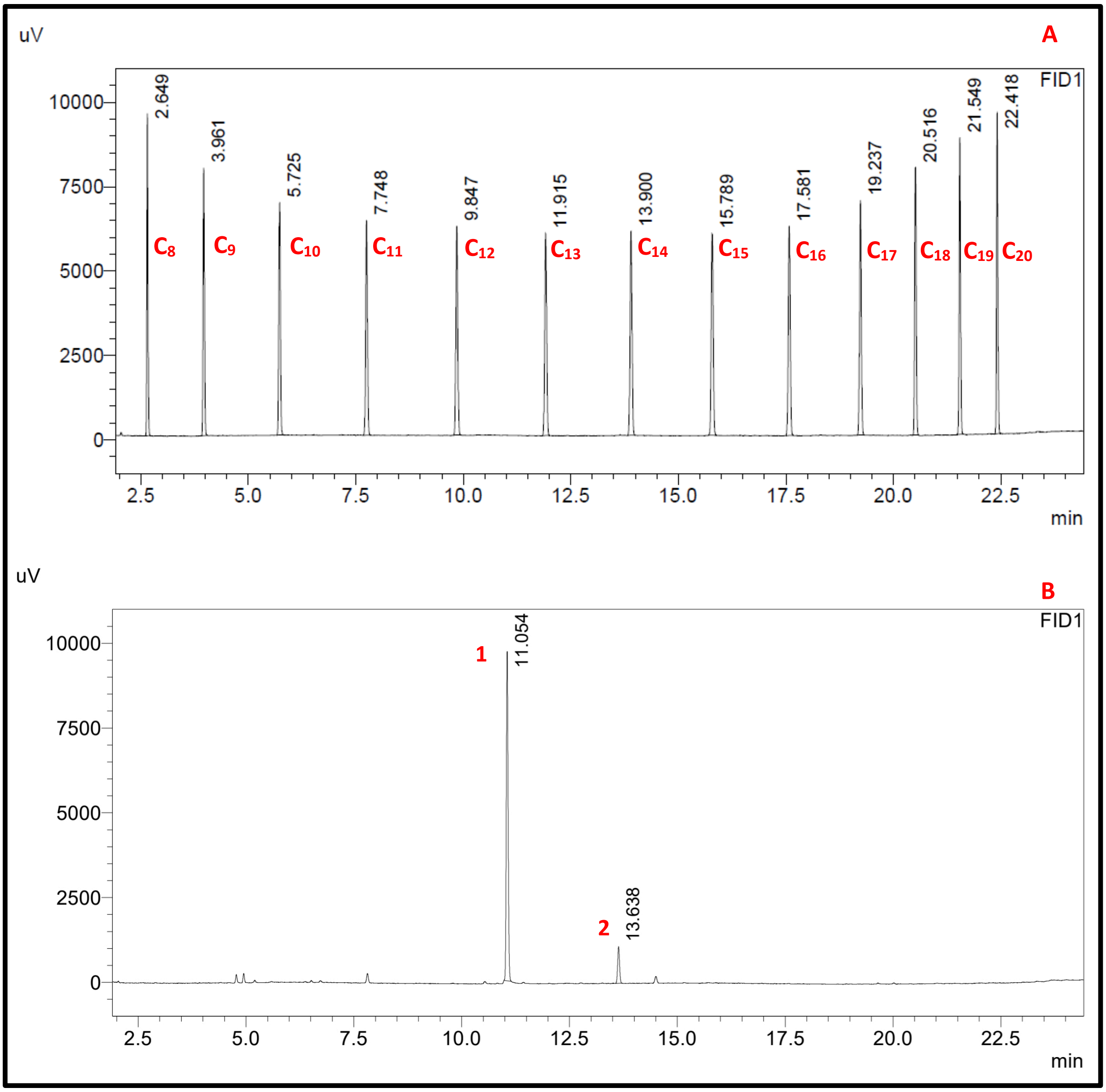

Figure S16: GC-FID Chromatographic profiles. A) Homologous hydrocarbon series, C_8-C_{20}. B) *Cymbopogon martini* commercial essential oil. Very minor peaks at five minutes are solvent impurities (hexane). The peak numbering is detailed in Table 1.

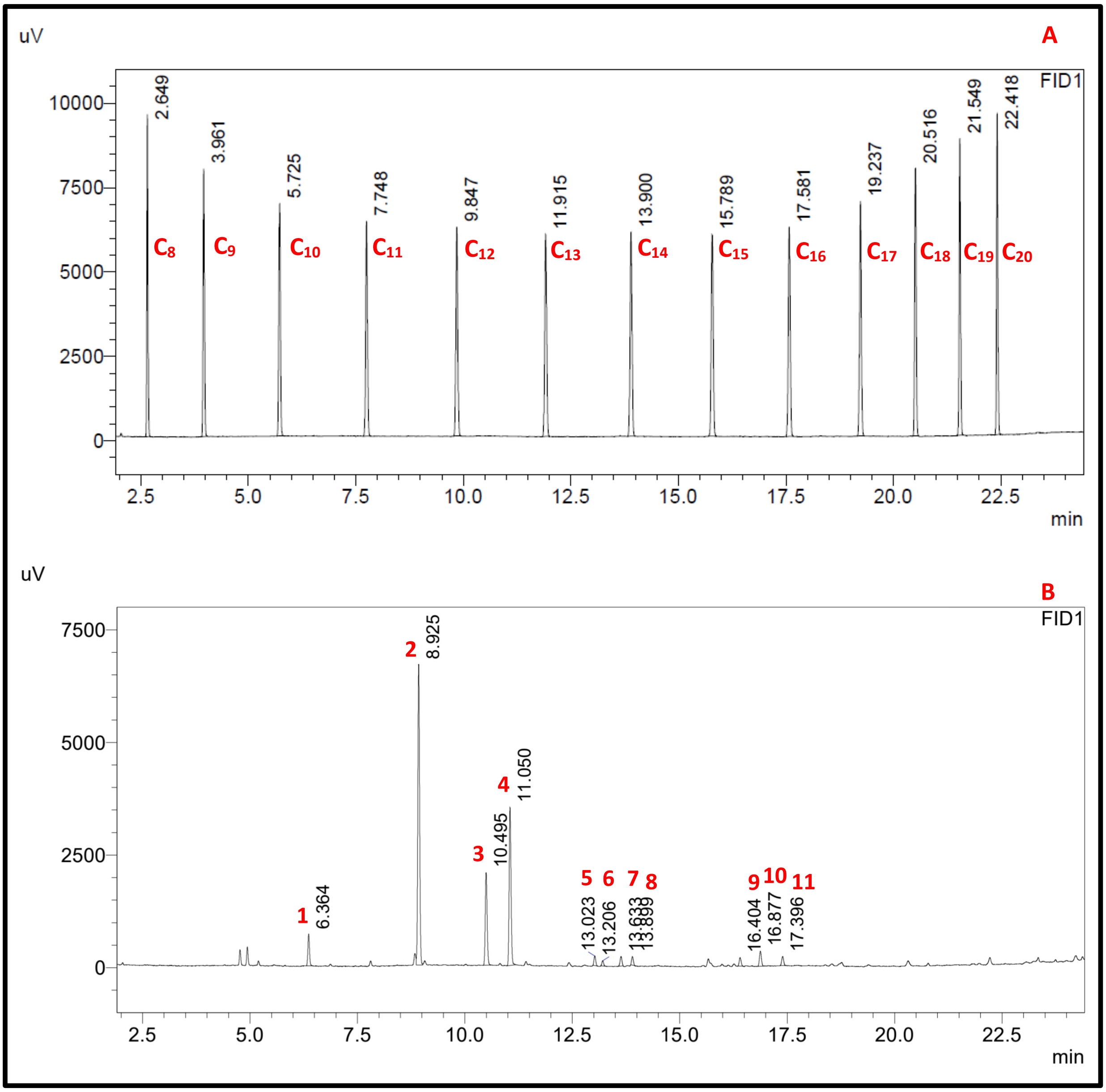

Figure S17: GC-FID Chromatographic profiles. A) Homologous hydrocarbon series, C_8-C_{20}. B) *Cymbopogon nardus* commercial essential oil. Very minor peaks at five minutes are solvent impurities (hexane). The peak numbering is detailed in Table 1.

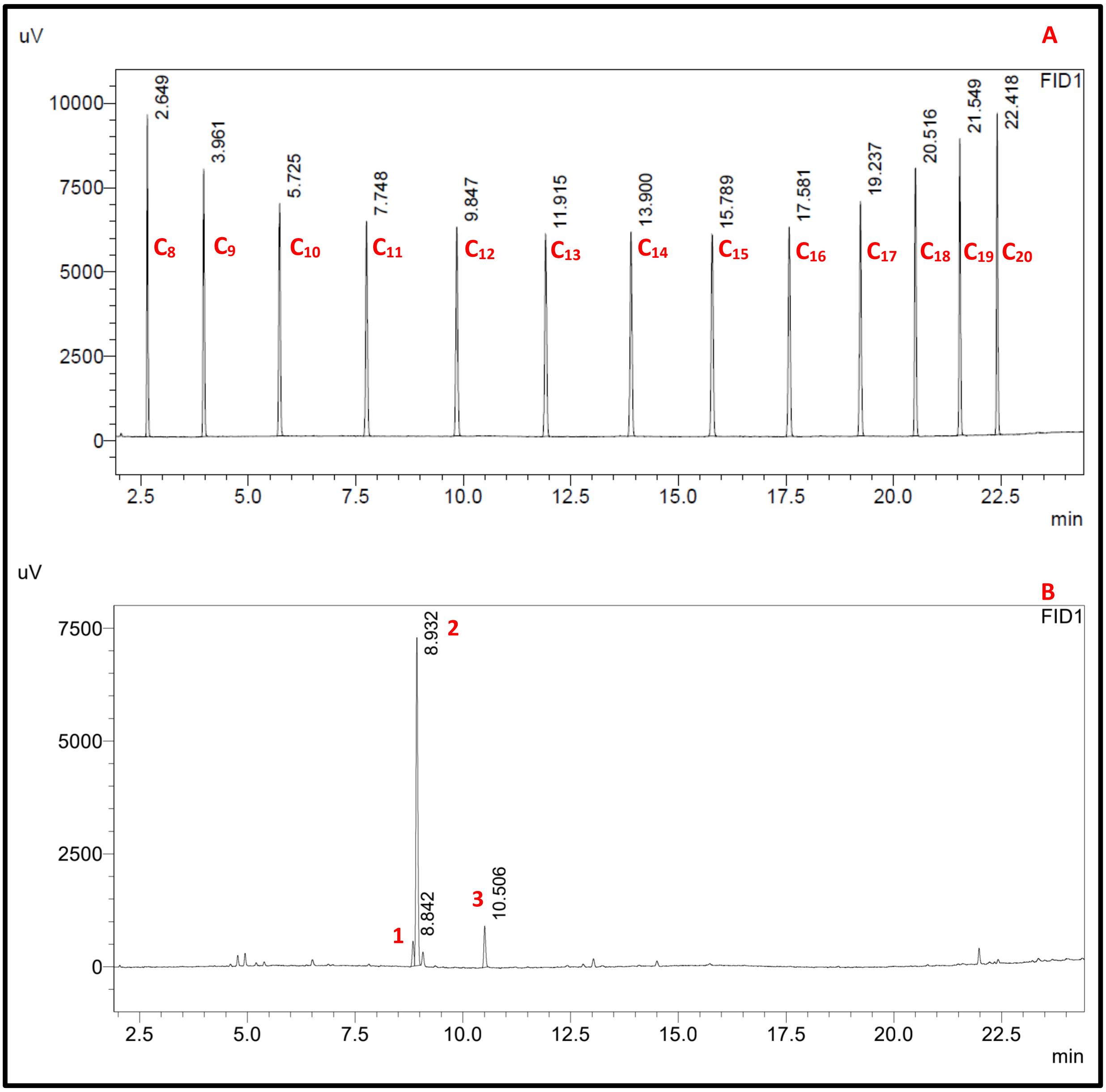

Figure S18: GC-FID Chromatographic profiles. A) Homologous hydrocarbon series, C_8-C_{20}. B) *Eucalyptus citriodora* commercial essential oil. Very minor peaks at five minutes are solvent impurities (hexane). The peak numbering is detailed in Table 1.

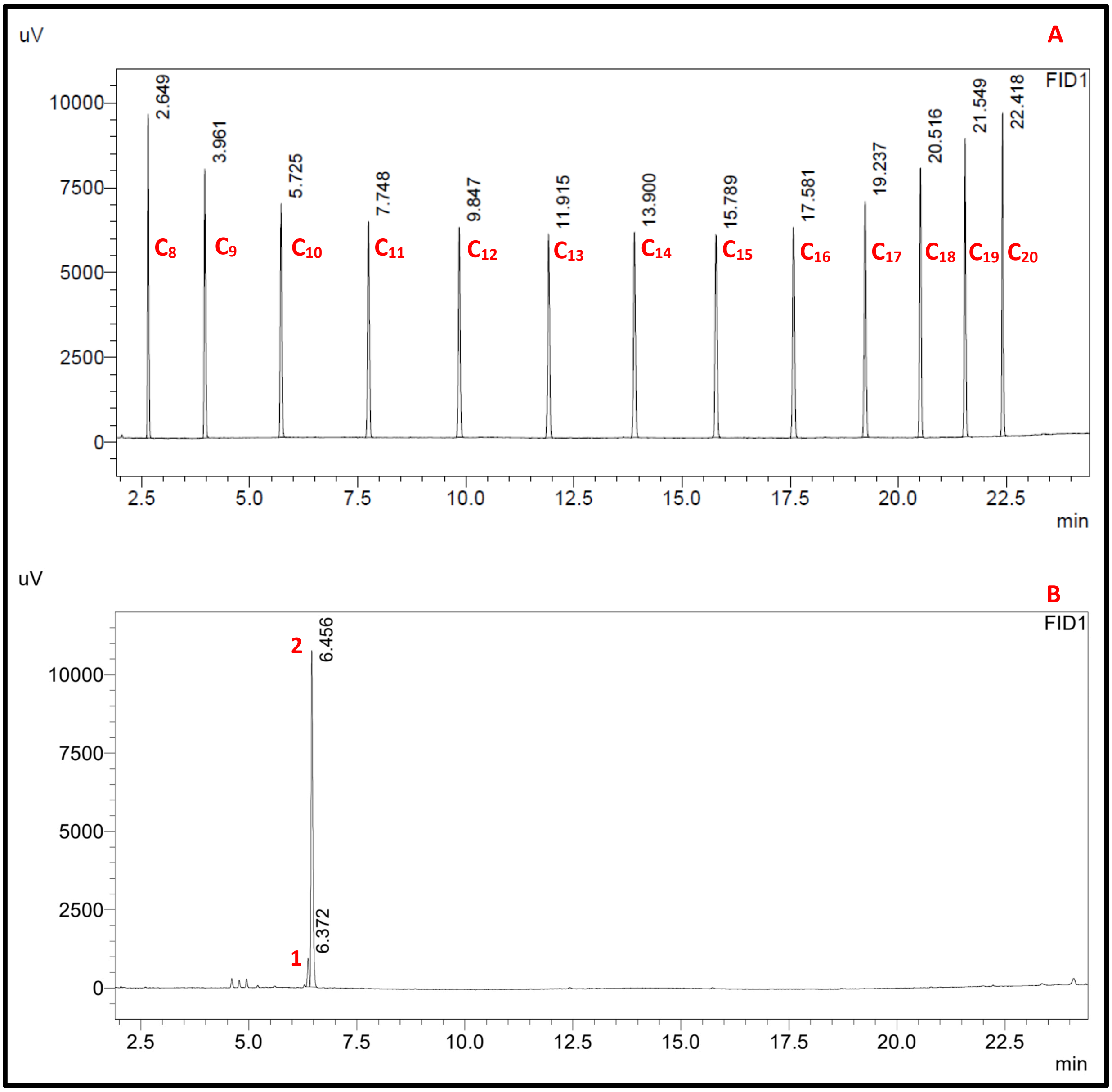

Figure S19: GC-FID Chromatographic profiles. A) Homologous hydrocarbon series, C_8-C_{20}. B) *Eucalyptus globulus* commercial essential oil. Very minor peaks at five minutes are solvent impurities (hexane). The peak numbering is detailed in Table 1.

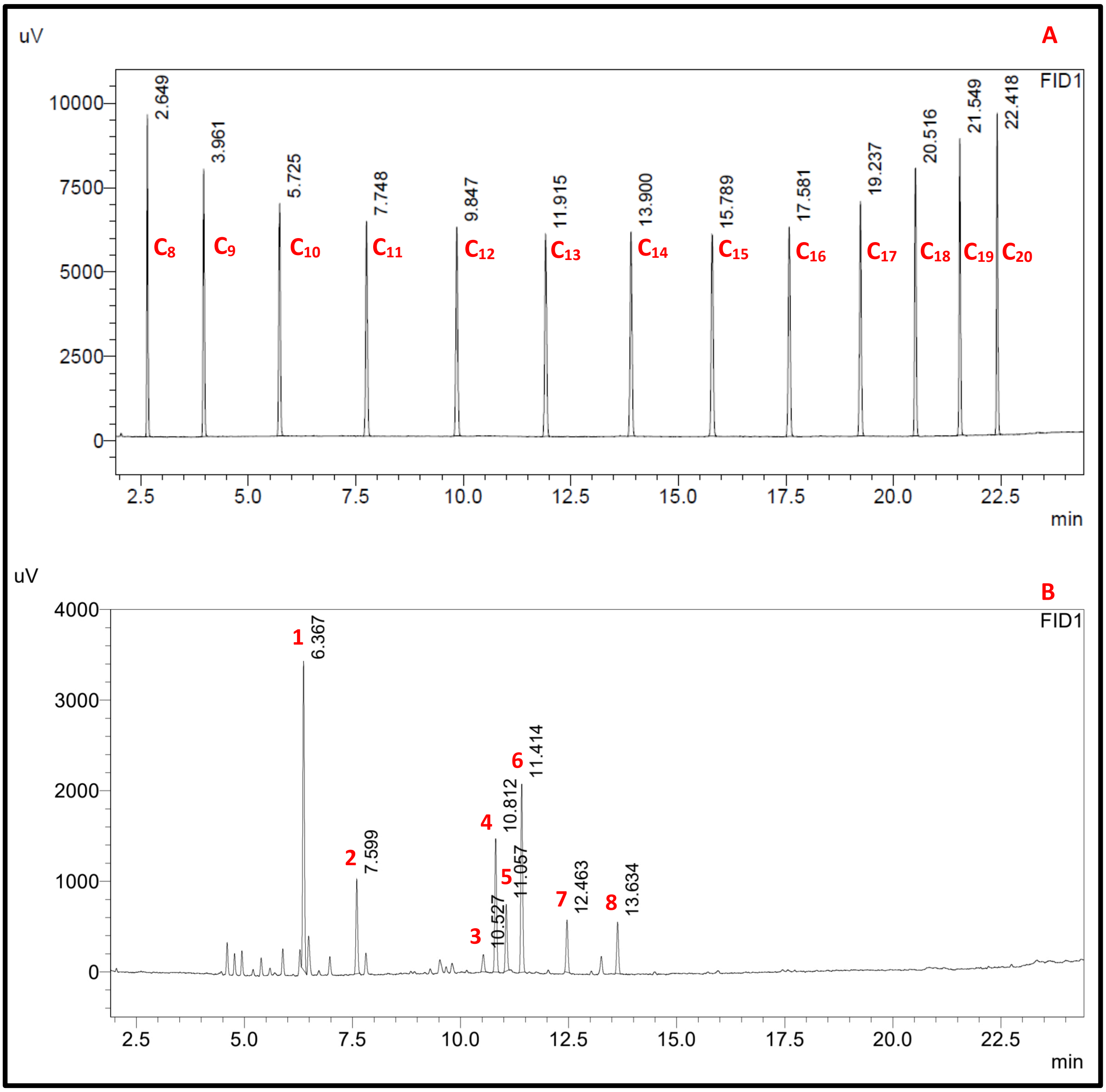

Figure S20: GC-FID Chromatographic profiles. A) Homologous hydrocarbon series, C_8-C_{20}. B) *Eucalyptus staigeriana* commercial essential oil. Very minor peaks at five minutes are solvent impurities (hexane). The peak numbering is detailed in Table 1.

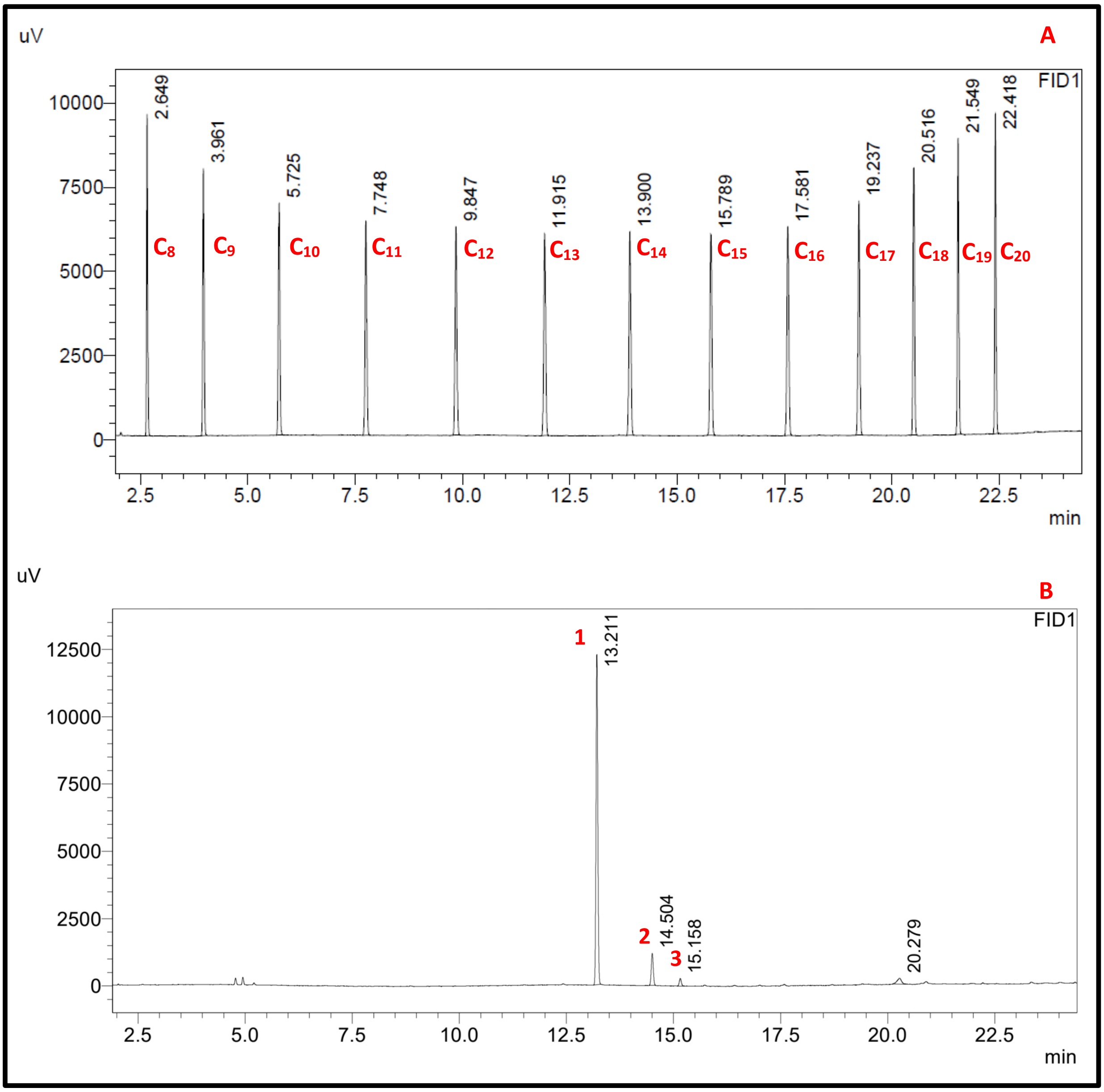

Figure S21: GC-FID Chromatographic profiles. A) Homologous hydrocarbon series, C_8-C_{20}. B) *Eugenia caryophyllus* commercial essential oil. Very minor peaks at five minutes are solvent impurities (hexane). The peak numbering is detailed in Table 1.

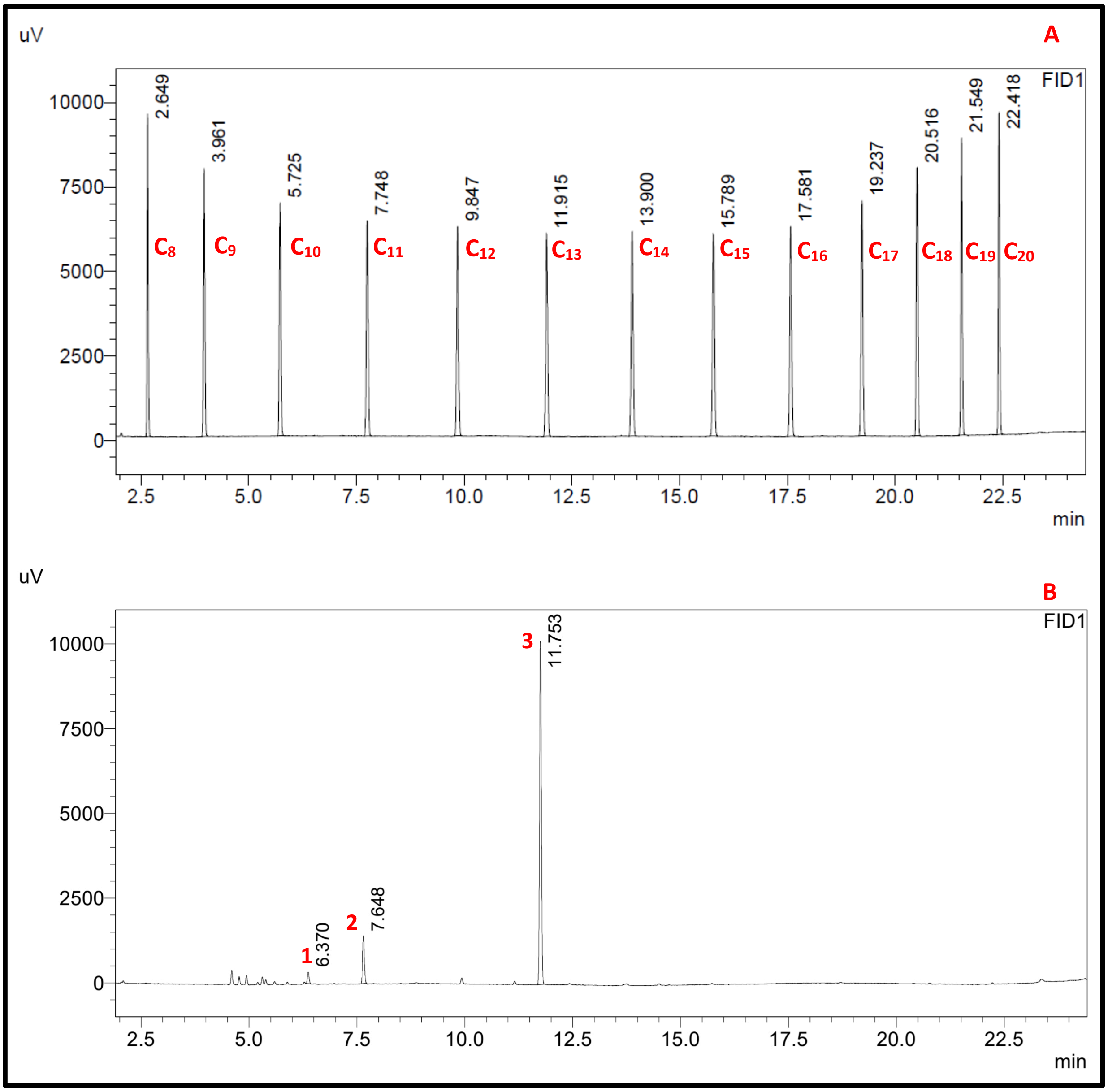

Figure S22: GC-FID Chromatographic profiles. A) Homologous hydrocarbon series, C_8-C_{20}. B) *Foeniculum vulgare* commercial essential oil. Very minor peaks at five minutes are solvent impurities (hexane). The peak numbering is detailed in Table 1.

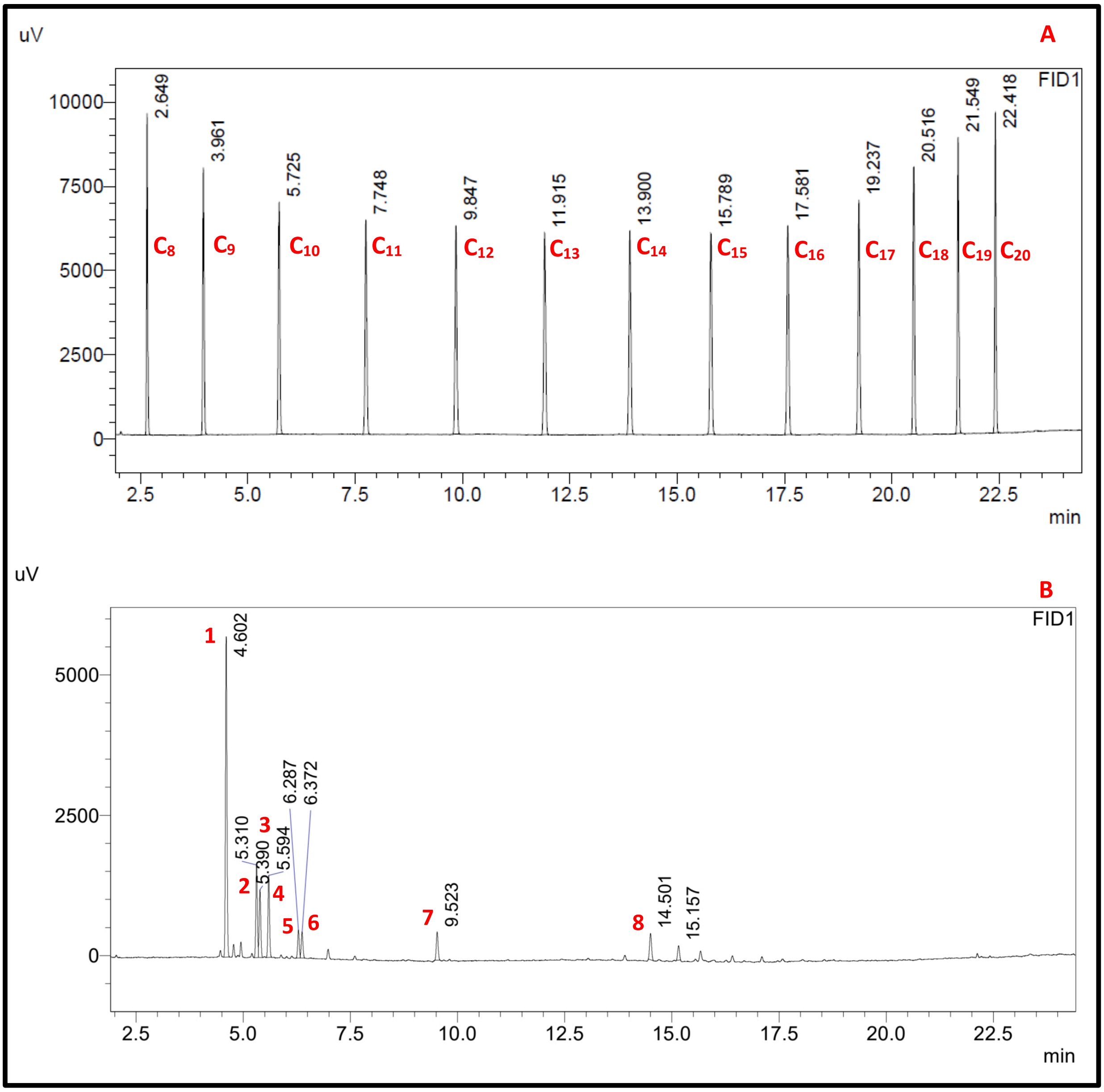

Figure S23: GC-FID Chromatographic profiles. A) Homologous hydrocarbon series, C_8-C_{20}. B) *Juniperus communis* commercial essential oil. Very minor peaks at five minutes are solvent impurities (hexane). The peak numbering detailed in Table 1.

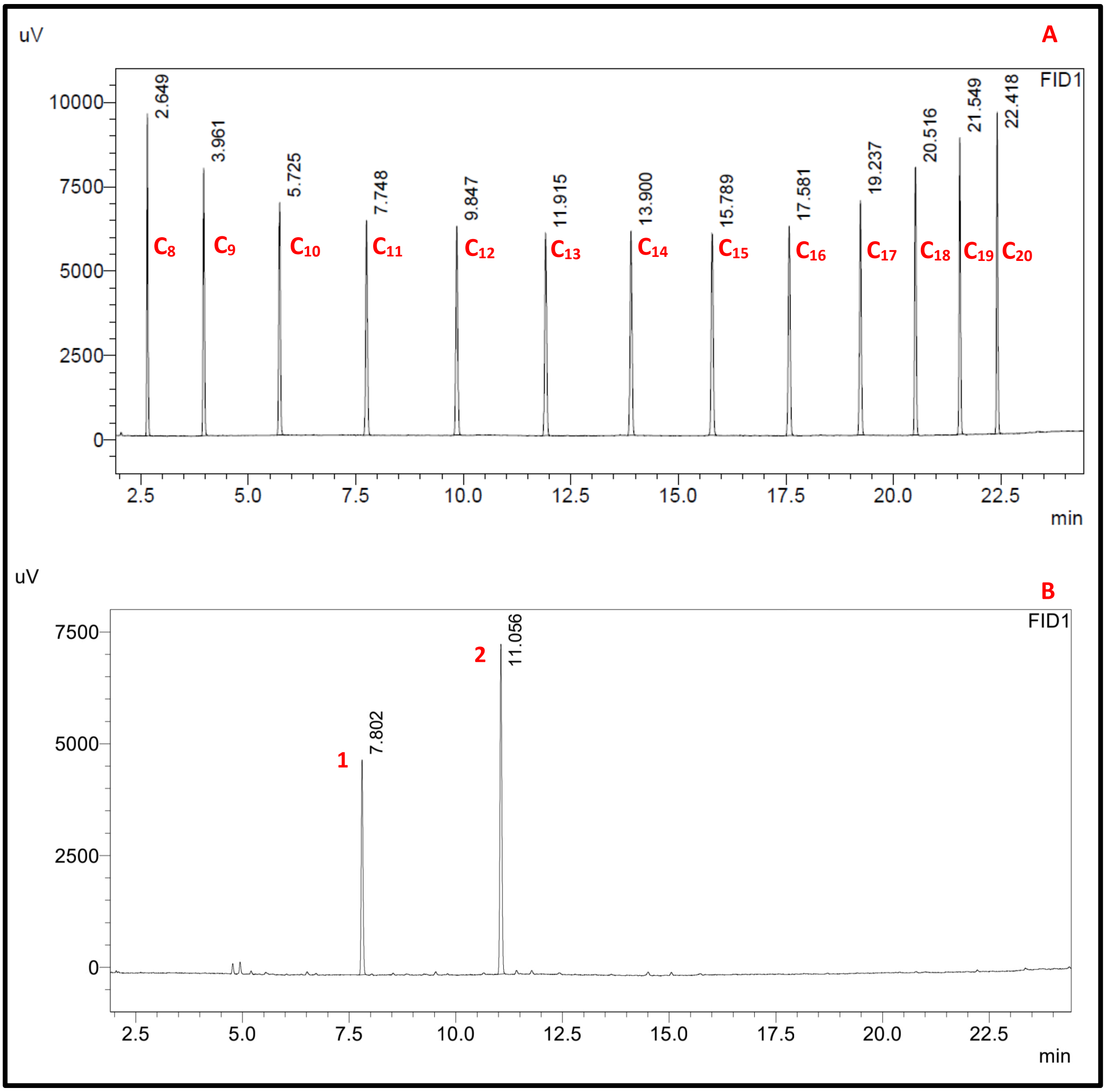

Figure S24: GC-FID Chromatographic profiles. A) Homologous hydrocarbon series, C_8-C_{20}. B) *Lavandula angustifolia* commercial essential oil. Very minor peaks at five minutes are solvent impurities (hexane). The peak numbering is detailed in Table 1.

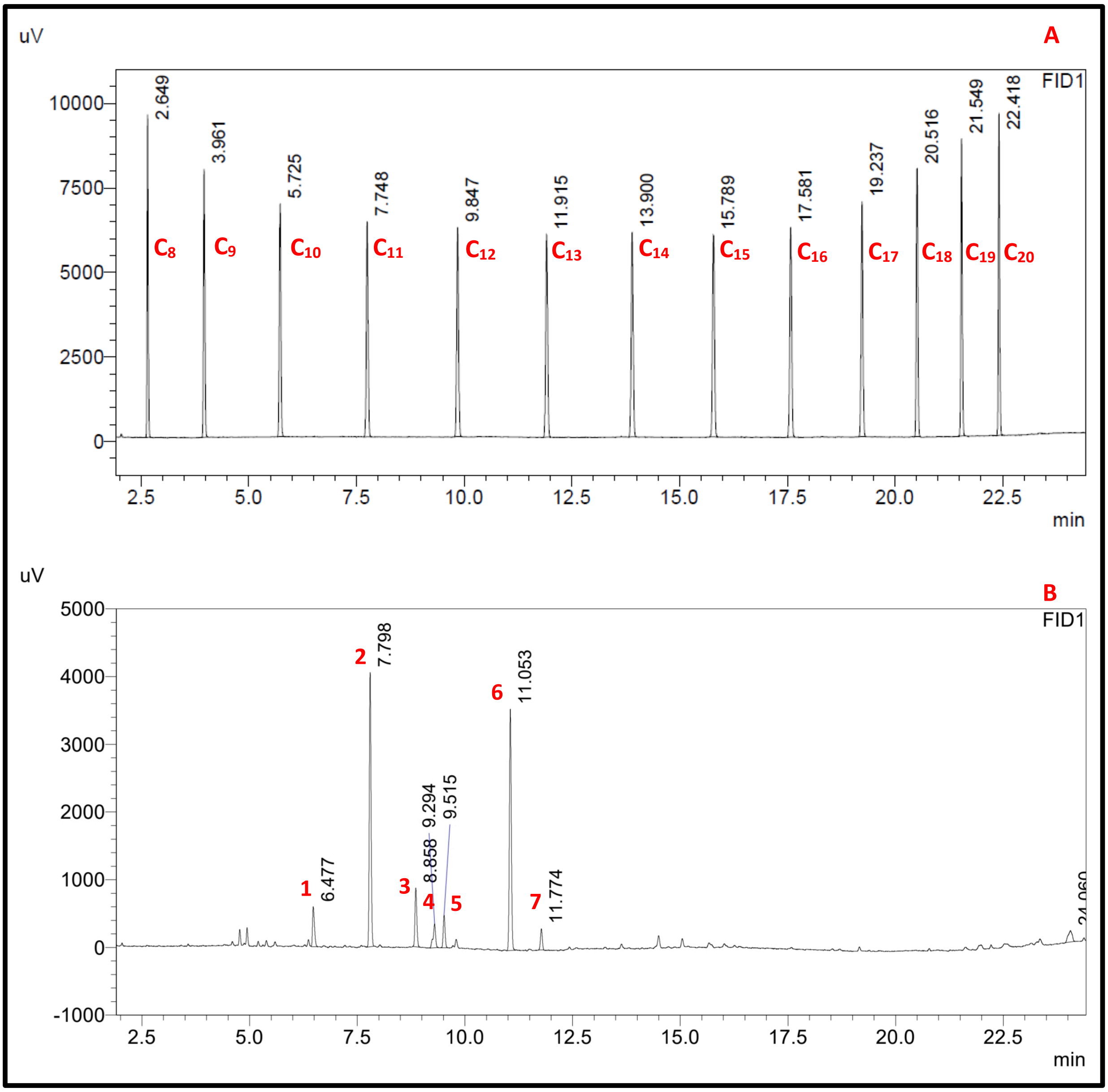

Figure S25: GC-FID Chromatographic profiles. A) Homologous hydrocarbon series, C_8-C_{20}. B) *Lavandula hybrida* commercial essential oil. Very minor peaks at five minutes are solvent impurities (hexane). The peak numbering is detailed in Table 1.

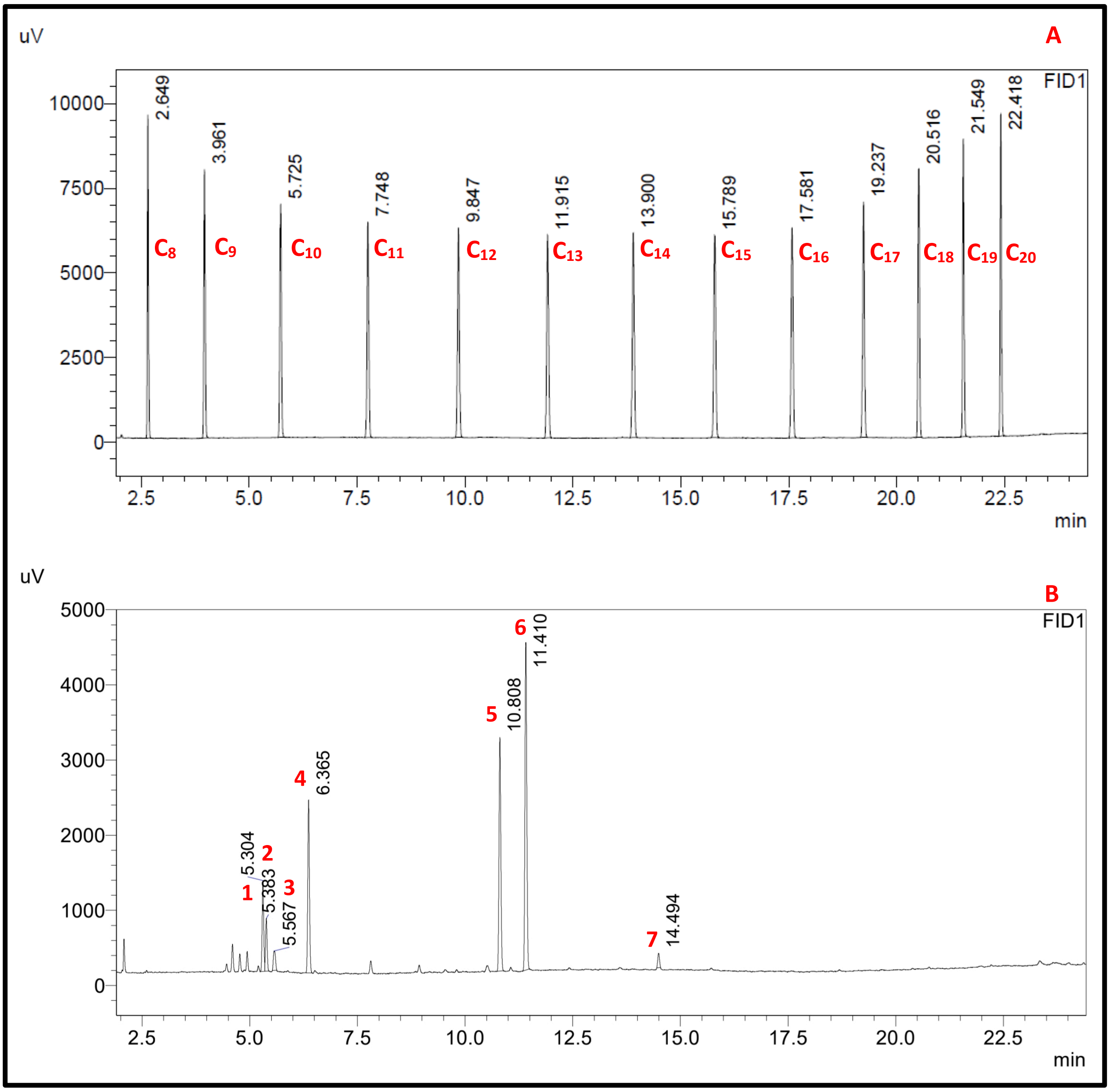

Figure S26: GC-FID Chromatographic profiles. A) Homologous hydrocarbon series, C_8-C_{20}. B) *Litsea cubeba* commercial essential oil. Very minor peaks at five minutes are solvent impurities (hexane). The peak numbering is detailed in Table 1.

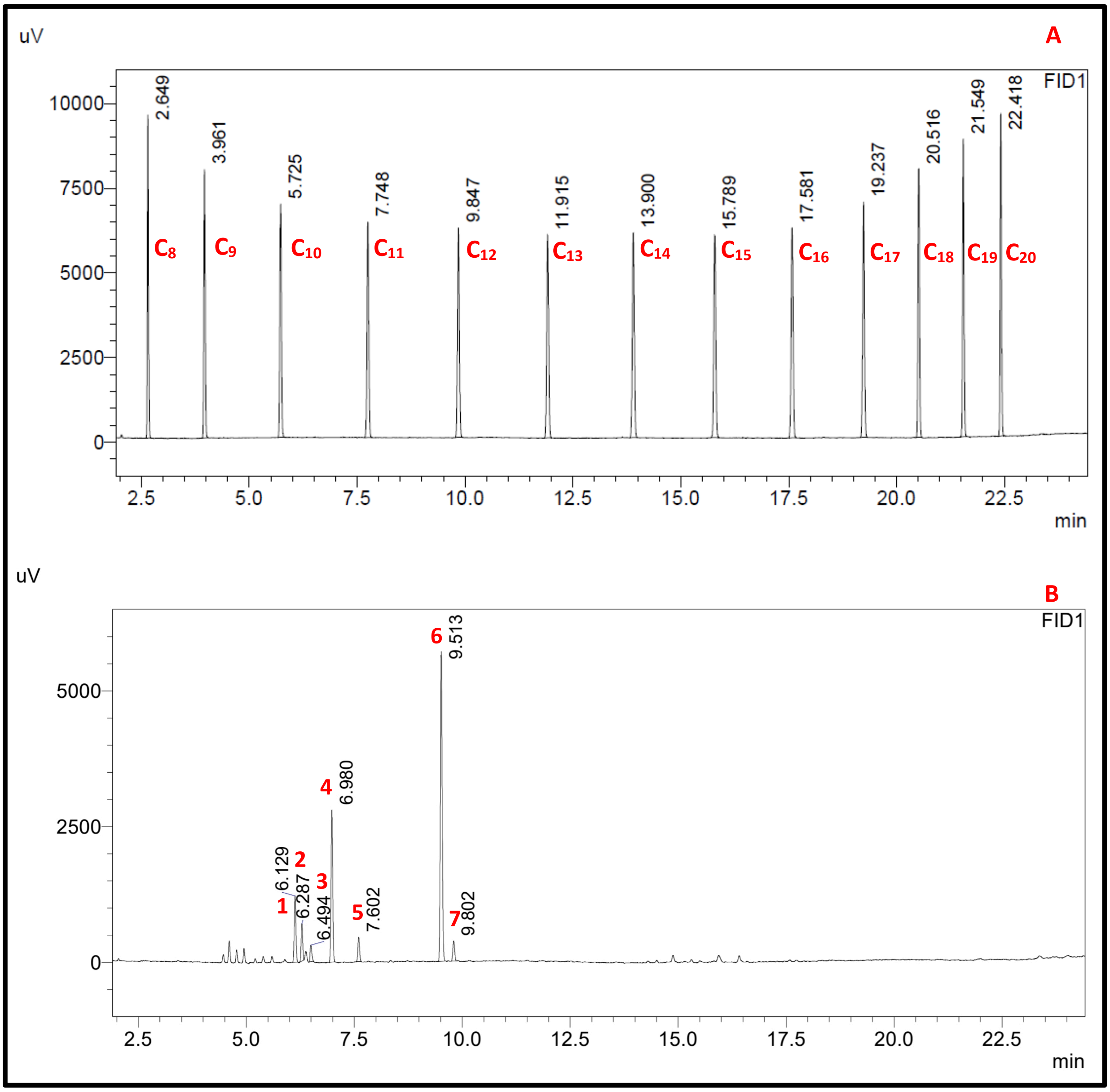

Figure S27: GC-FID Chromatographic profiles. A) Homologous hydrocarbon series, C_8-C_{20}. B) *Melaleuca alternifolia* commercial essential oil. Very minor peaks at five minutes are solvent impurities (hexane). The peak numbering is detailed in Table 1.

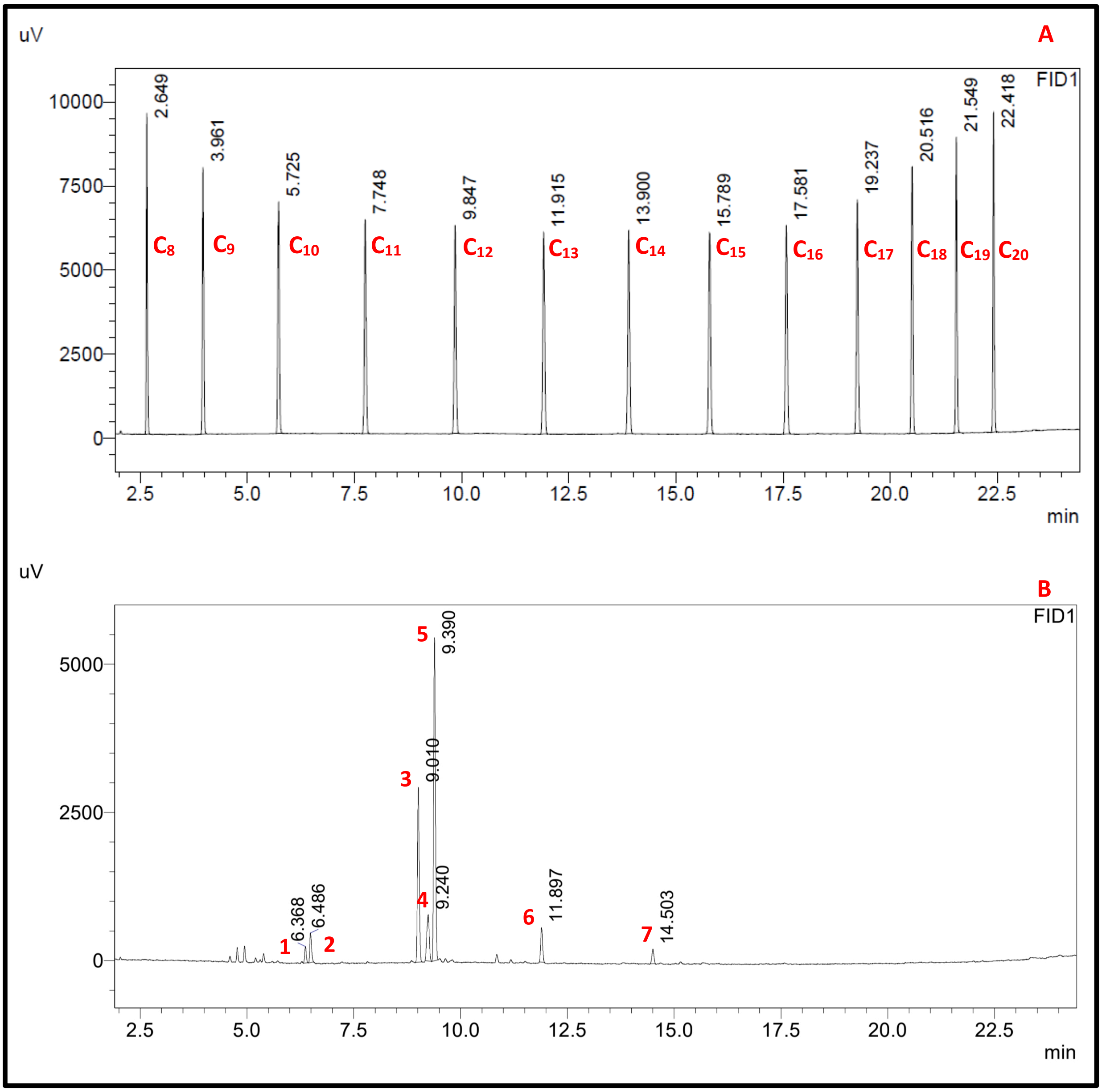

Figure S28: GC-FID Chromatographic profiles. A) Homologous hydrocarbon series, C_8-C_{20}. B) *Mentha piperita* commercial essential oil. Very minor peaks at five minutes are solvent impurities (hexane). The peak numbering is detailed in Table 1.

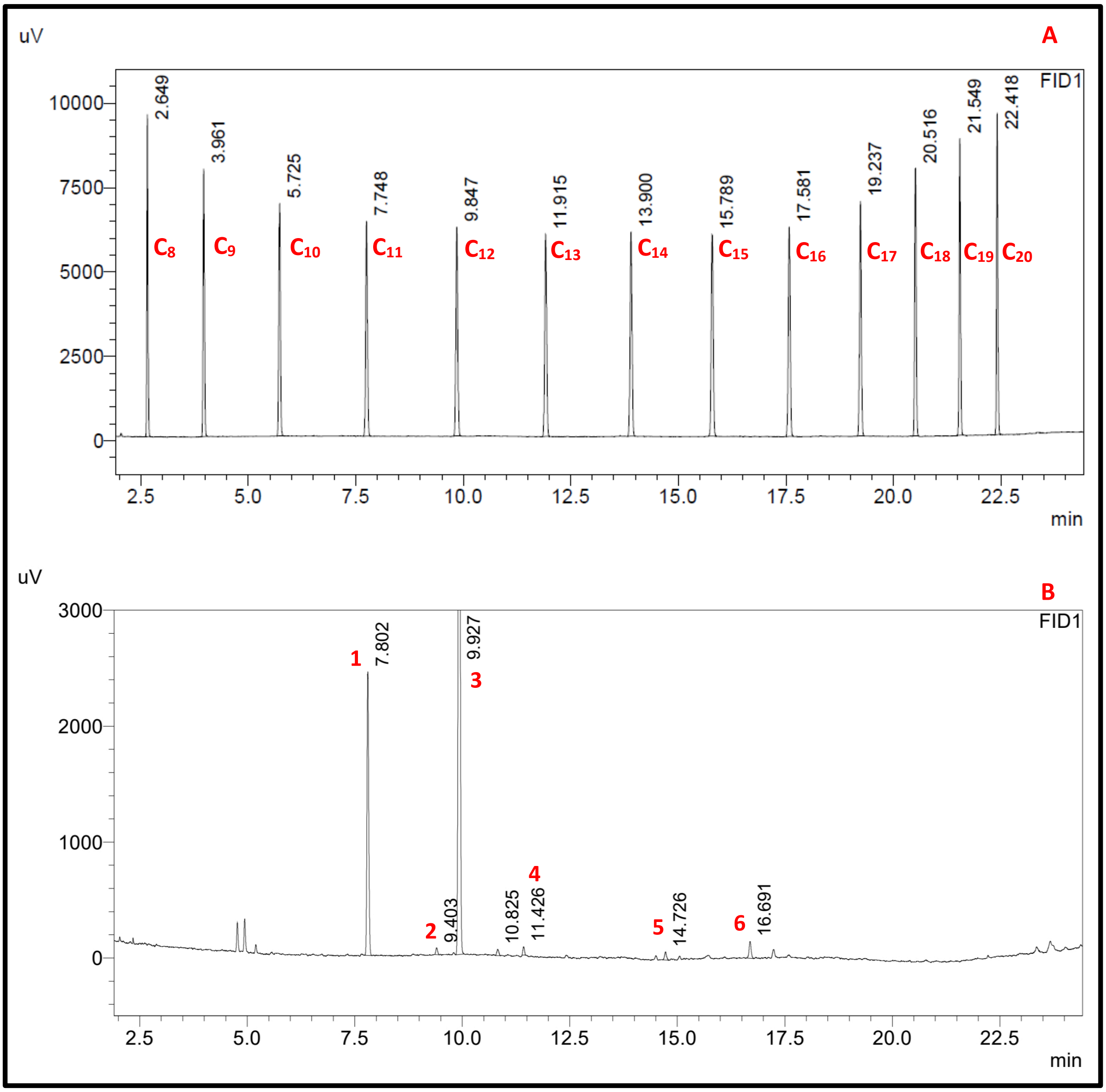

Figure S29: GC-FID Chromatographic profiles. A) Homologous hydrocarbon series, C_8-C_{20}. B) *Ocimum basilicum* commercial essential oil. Very minor peaks at five minutes are solvent impurities (hexane). The peak numbering is detailed in Table 1.

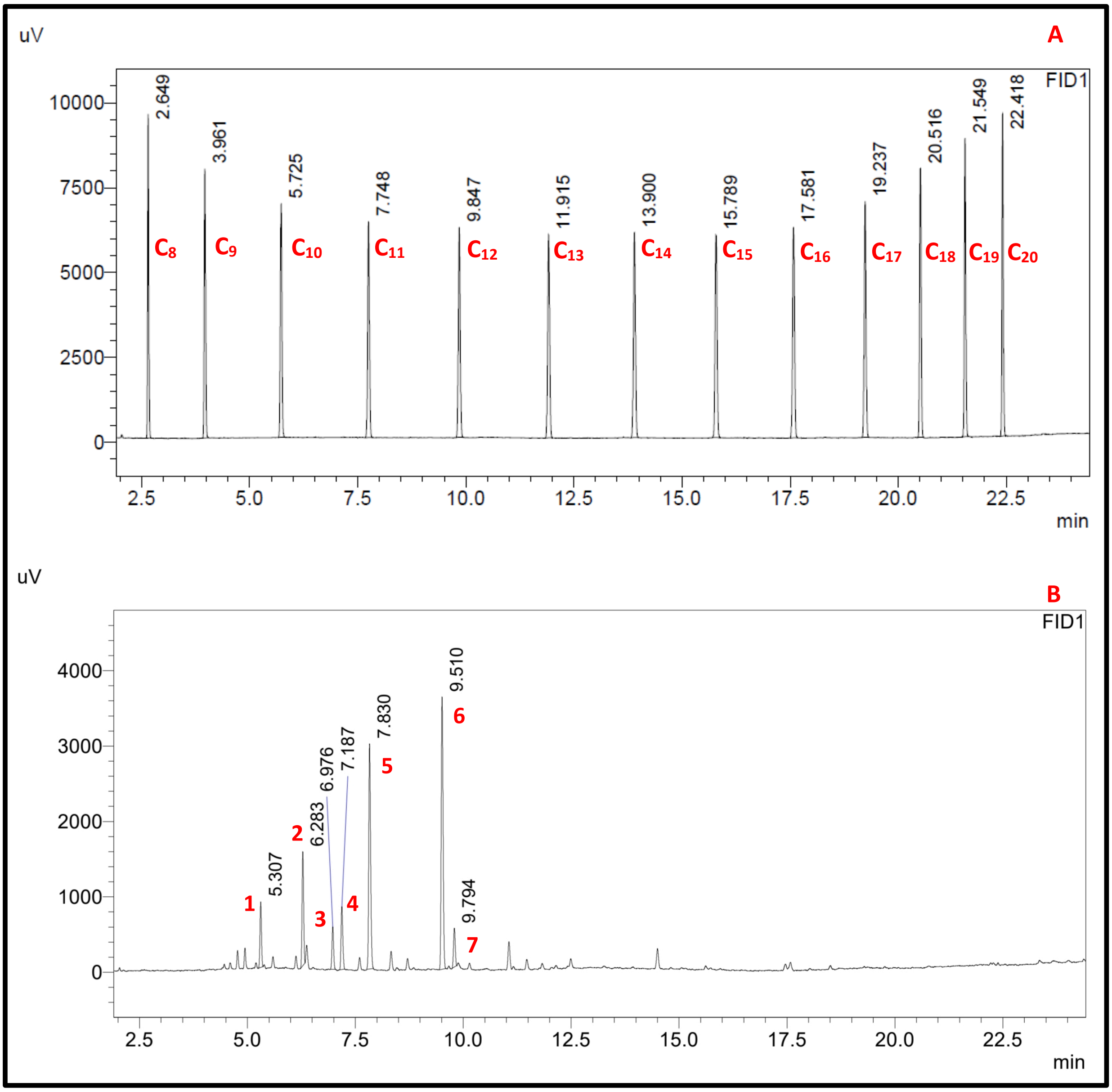

Figure S30: GC-FID Chromatographic profiles. A) Homologous hydrocarbon series, C_8-C_{20}. B) *Origanum majorana* commercial essential oil. Very minor peaks at five minutes are solvent impurities (hexane). The peak numbering is detailed in Table 1.

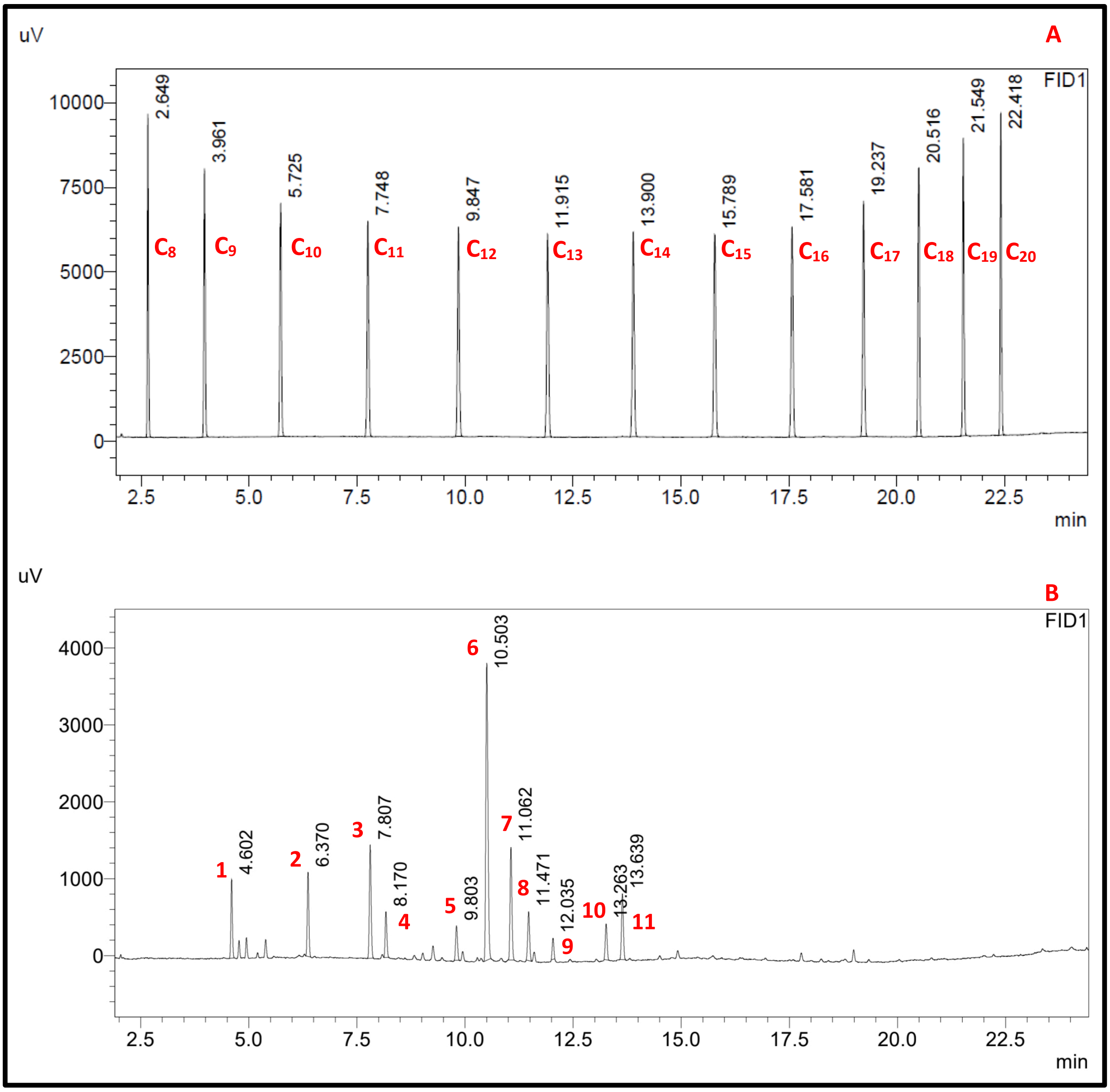

Figure S31: GC-FID Chromatographic profiles. A) Homologous hydrocarbon series, C_8-C_{20}. B) *Pelargonium graveolens* commercial essential oil. Very minor peaks at five minutes are solvent impurities (hexane). The peak numbering is detailed in Table 1.

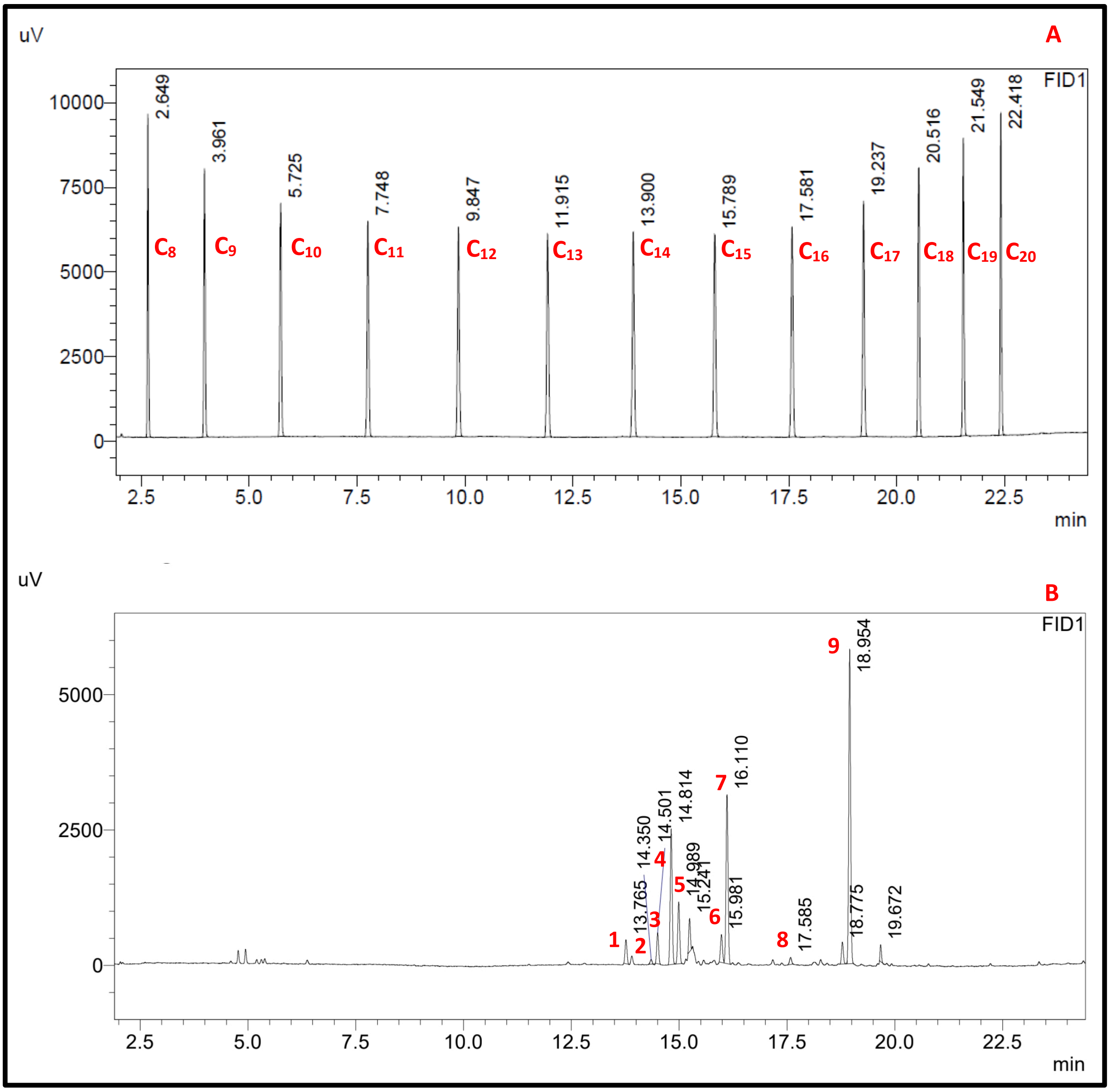

Figure S32: GC-FID Chromatographic profiles. A) Homologous hydrocarbon series, C_8-C_{20}. B) *Pogostemon cablin* commercial essential oil. Very minor peaks at five minutes are solvent impurities (hexane). The peak numbering is detailed in Table 1.

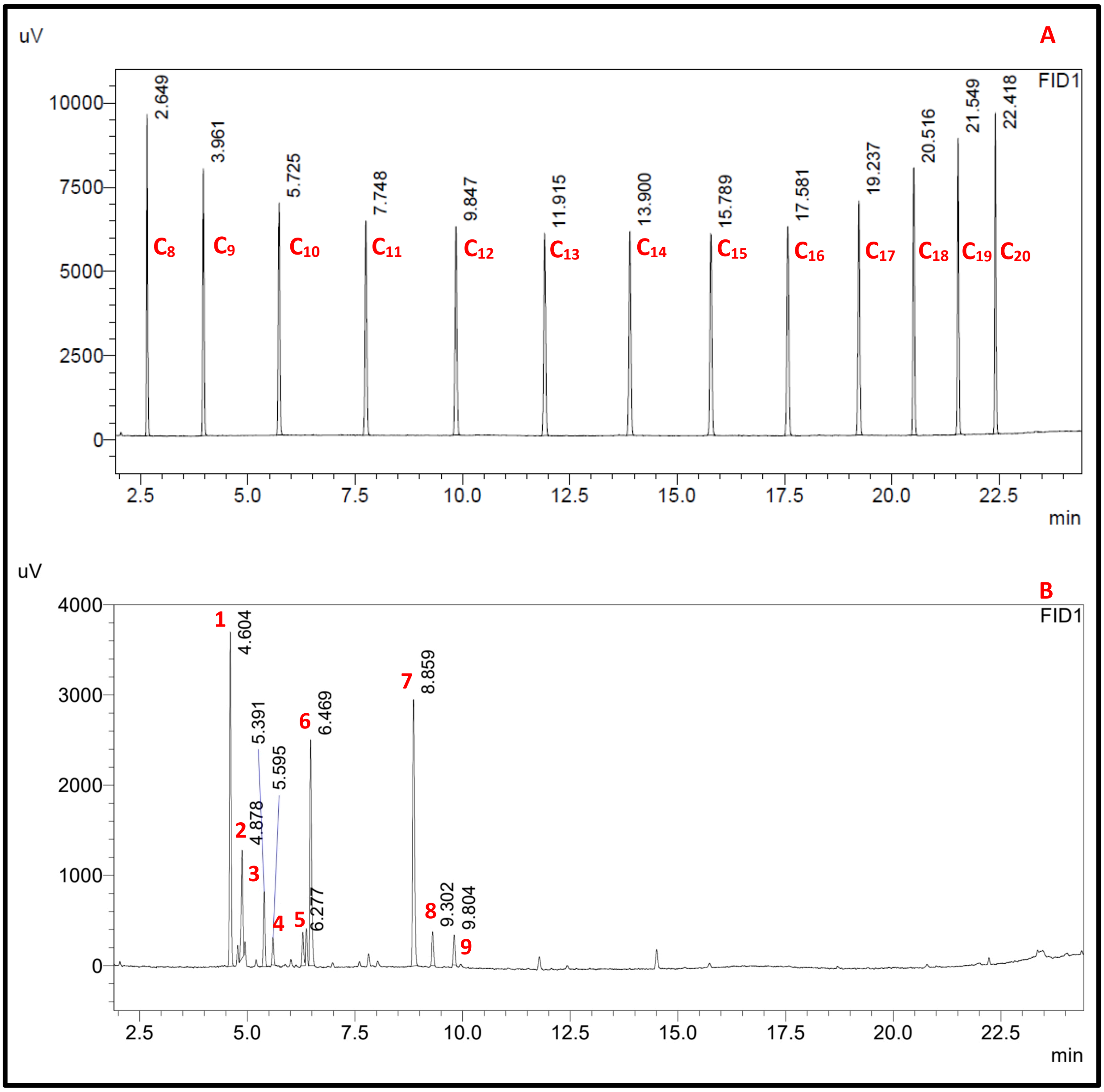

Figure S33: GC-FID Chromatographic profiles. A) Homologous hydrocarbon series, C_8-C_{20}. B) *Rosmarinus officinalis* commercial essential oil. Very minor peaks at five minutes are solvent impurities (hexane). The peak numbering is detailed in Table 1.

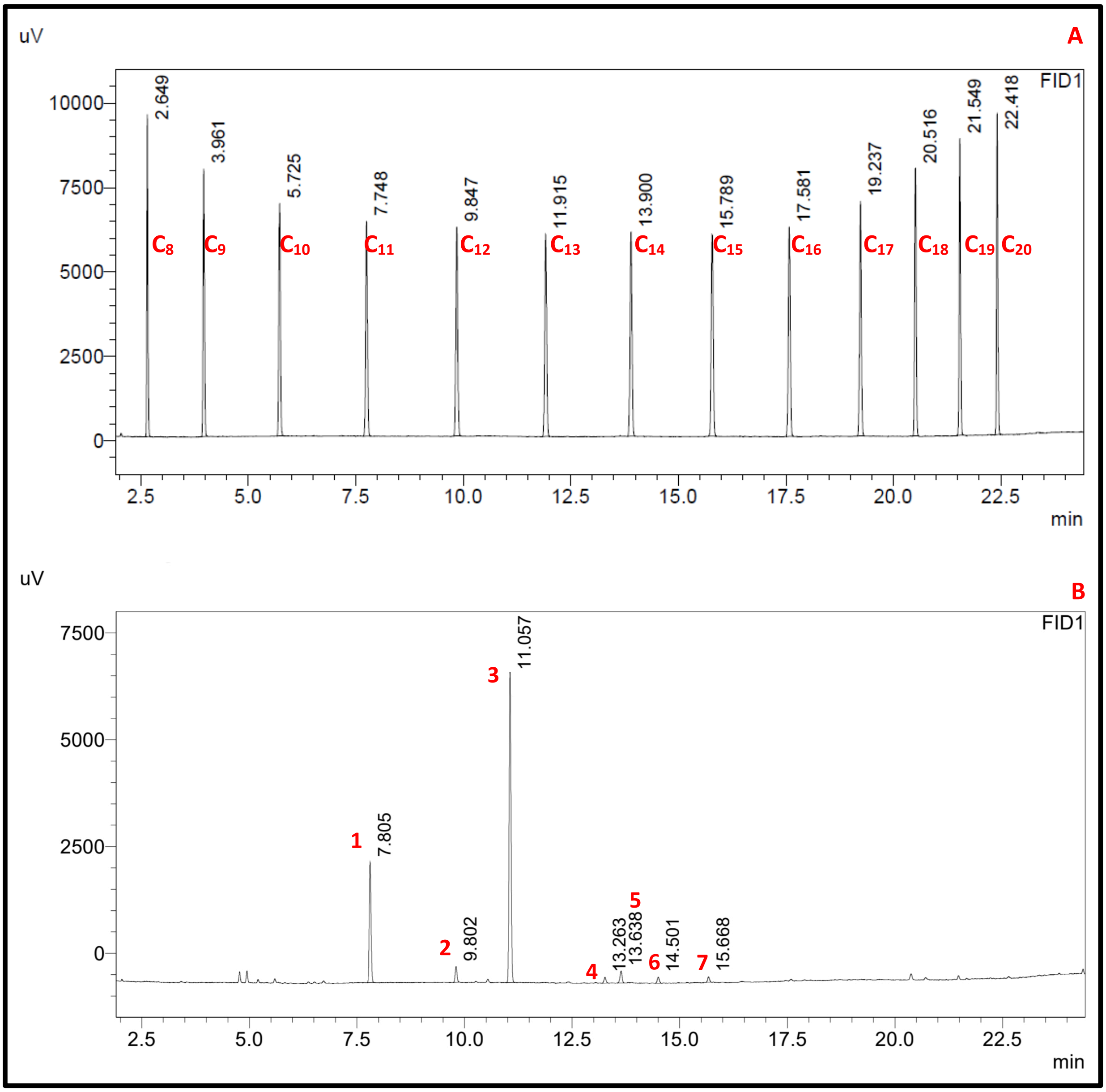

Figure S34: GC-FID Chromatographic profiles. A) Homologous hydrocarbon series, C_8-C_{20}. B) *Salvia sclarea* commercial essential oil. Very minor peaks at five minutes are solvent impurities (hexane). The peak numbering is detailed in Table 1.

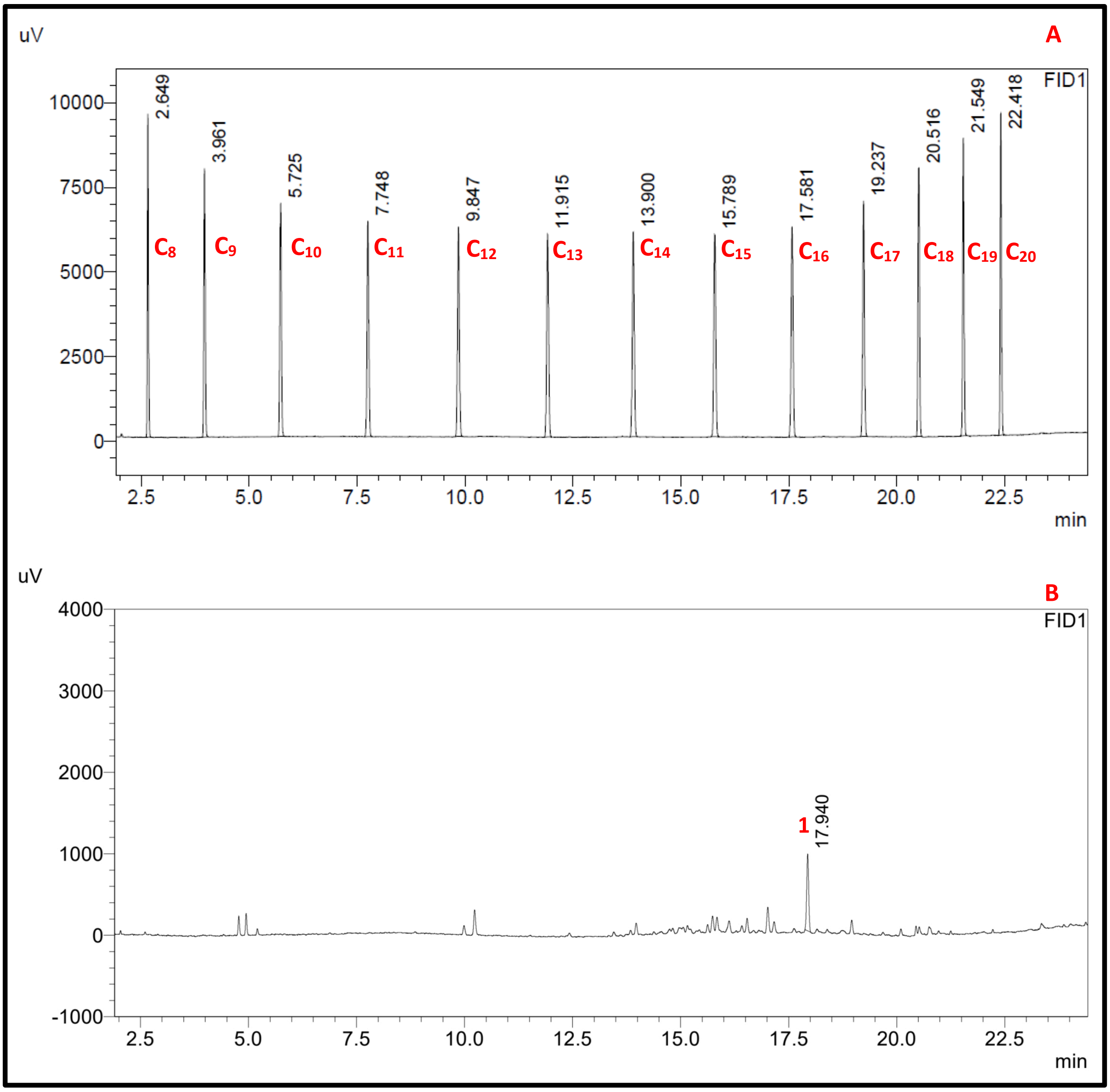

Figure S35: GC-FID Chromatographic profiles. A) Homologous hydrocarbon series, C_8-C_{20}. B) *Vetiveria zizanioides* commercial essential oil. Very minor peaks at five minutes are solvent impurities (hexane). The peak numbering is detailed in Table 1.

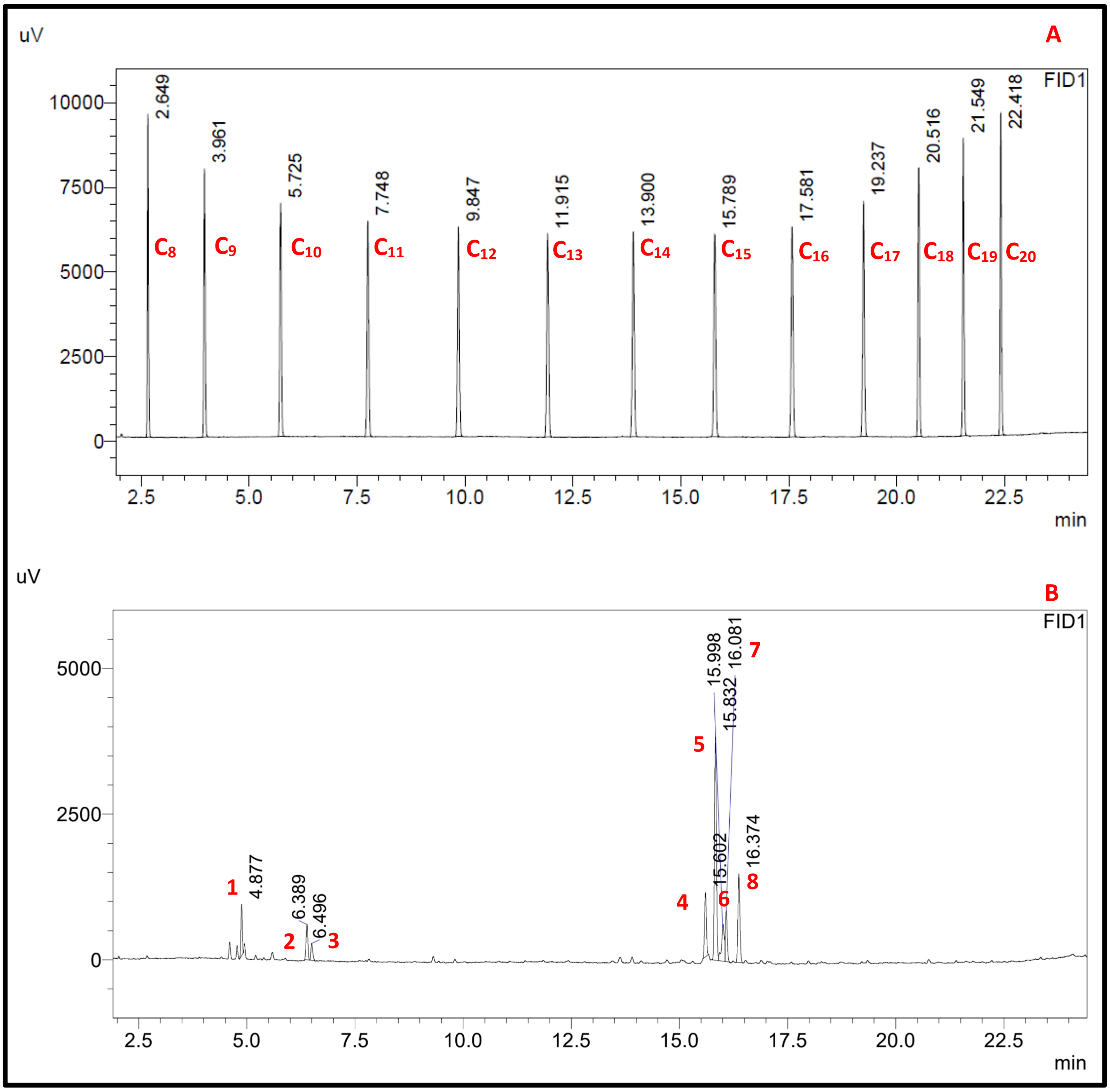

Figure S36: GC-FID Chromatographic profiles. A) Homologous hydrocarbon series, C_8-C_{20}. B) *Zingiber officinale* commercial essential oil. Very minor peaks at five minutes are solvent impurities (hexane). The peak numbering is detailed in Table 1.

2. GC-FID Chromatographic profiles from volatile compounds (Figures SI37 - SI47).

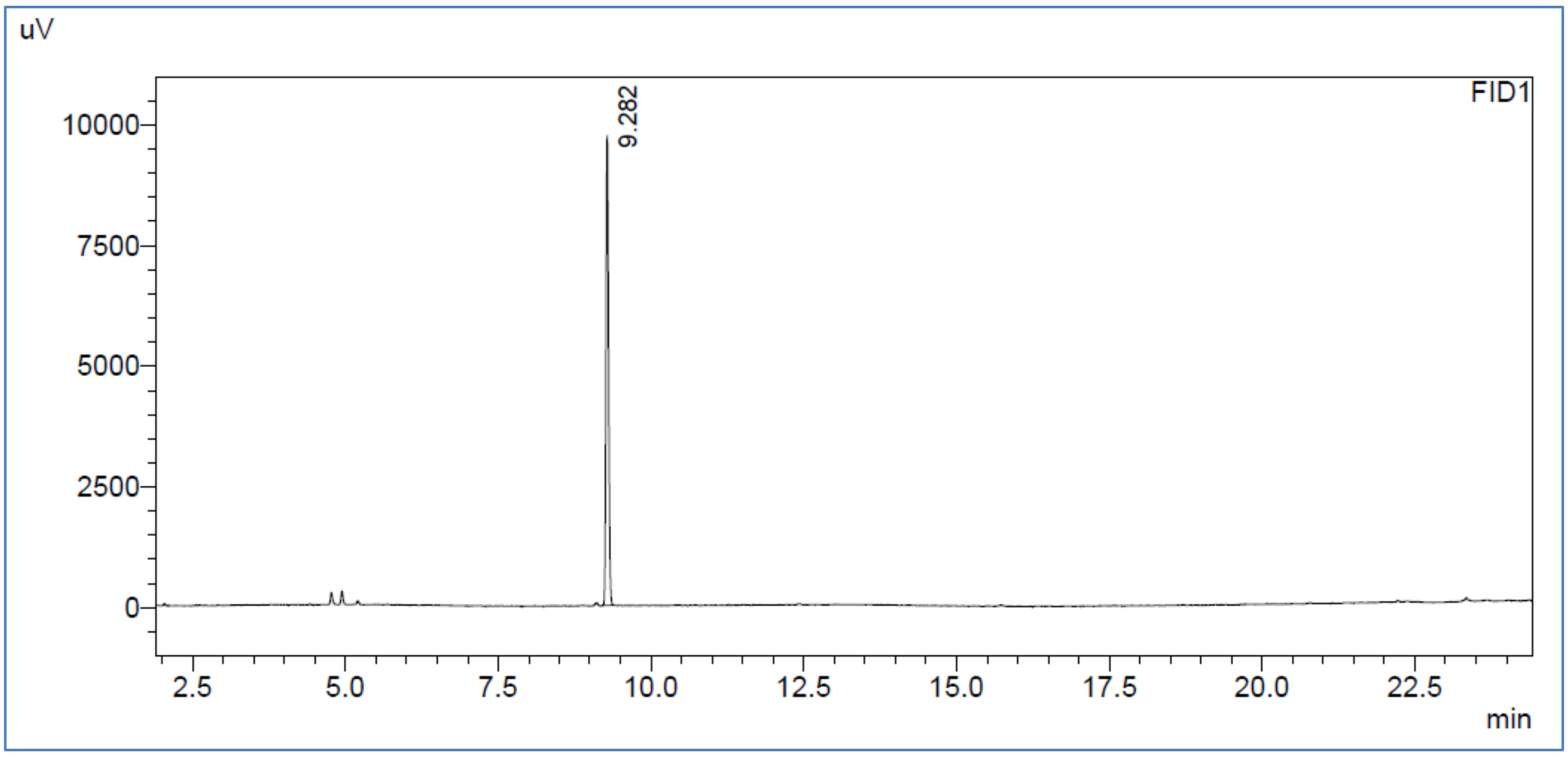

Figure S37: GC-FID Chromatographic profile of the borneol with 98% purity. Very minor peaks at five minutes are solvent impurities (hexane).

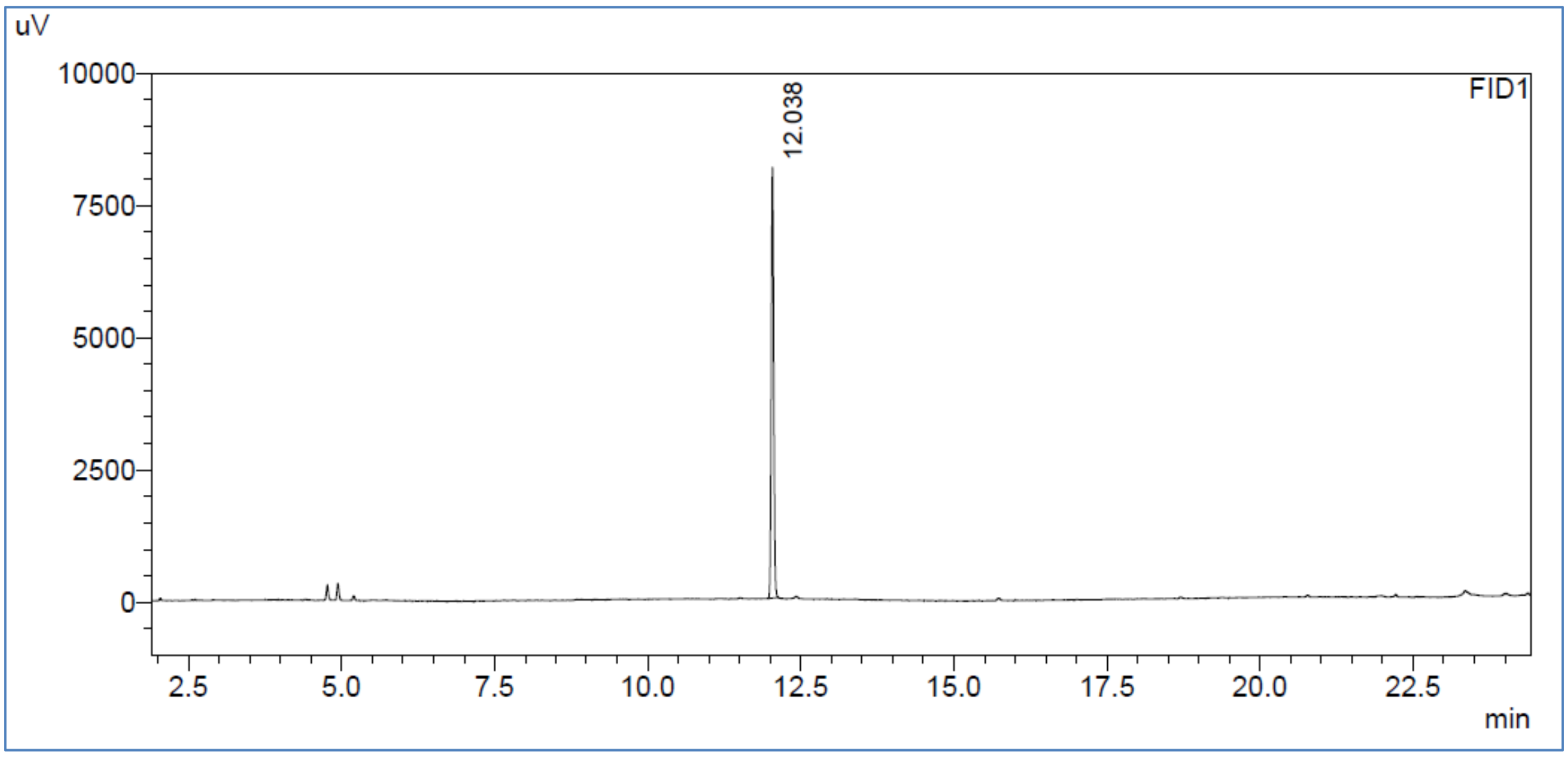

Figure S38: GC-FID Chromatographic profile of the carvacrol with 98% purity. Very minor peaks at five minutes are solvent impurities (hexane).

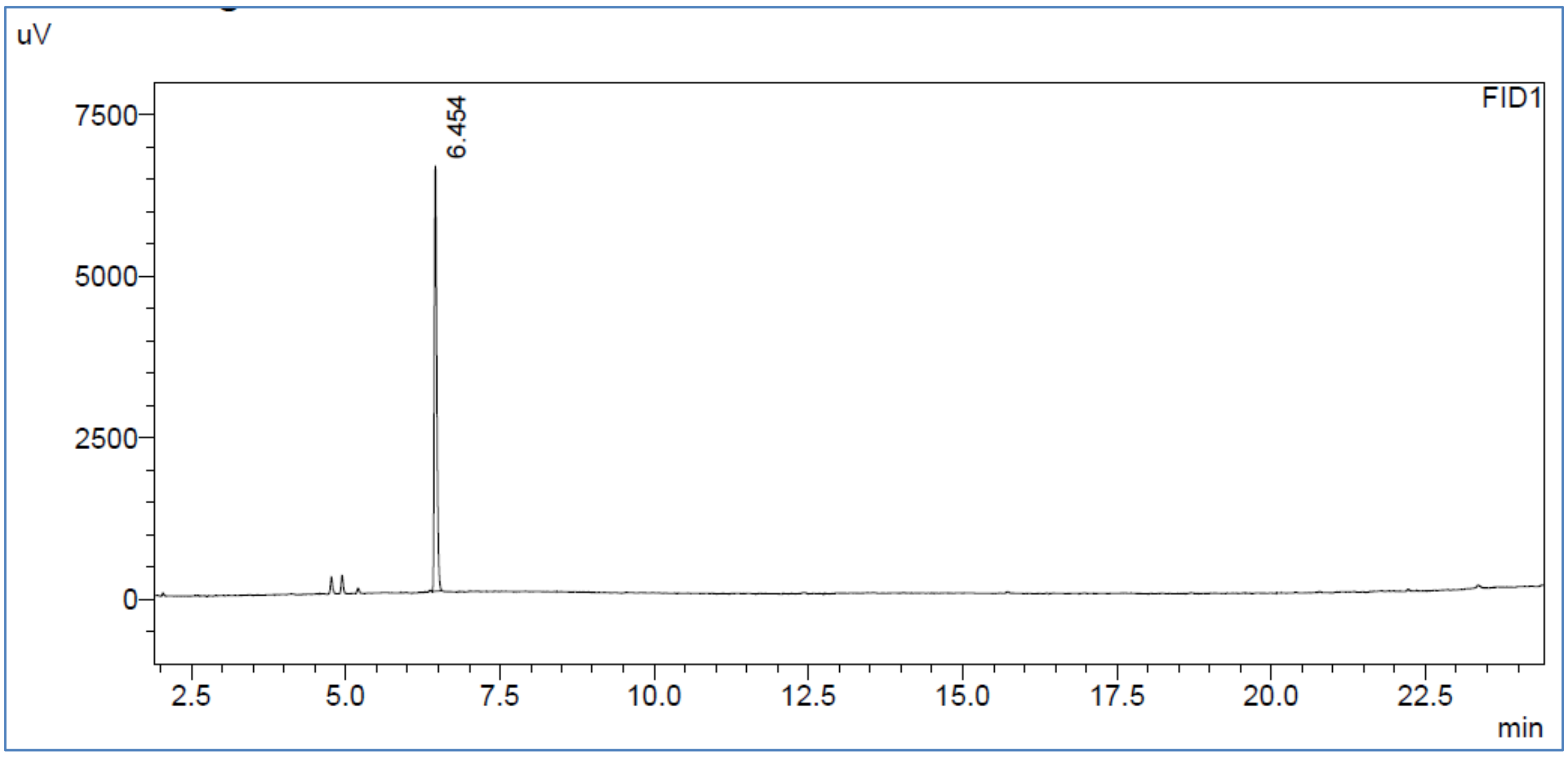

Figure S39: GC-FID Chromatographic profile of the 1,8-cineole with 98% purity. Very minor peaks at five minutes are solvent impurities (hexane).

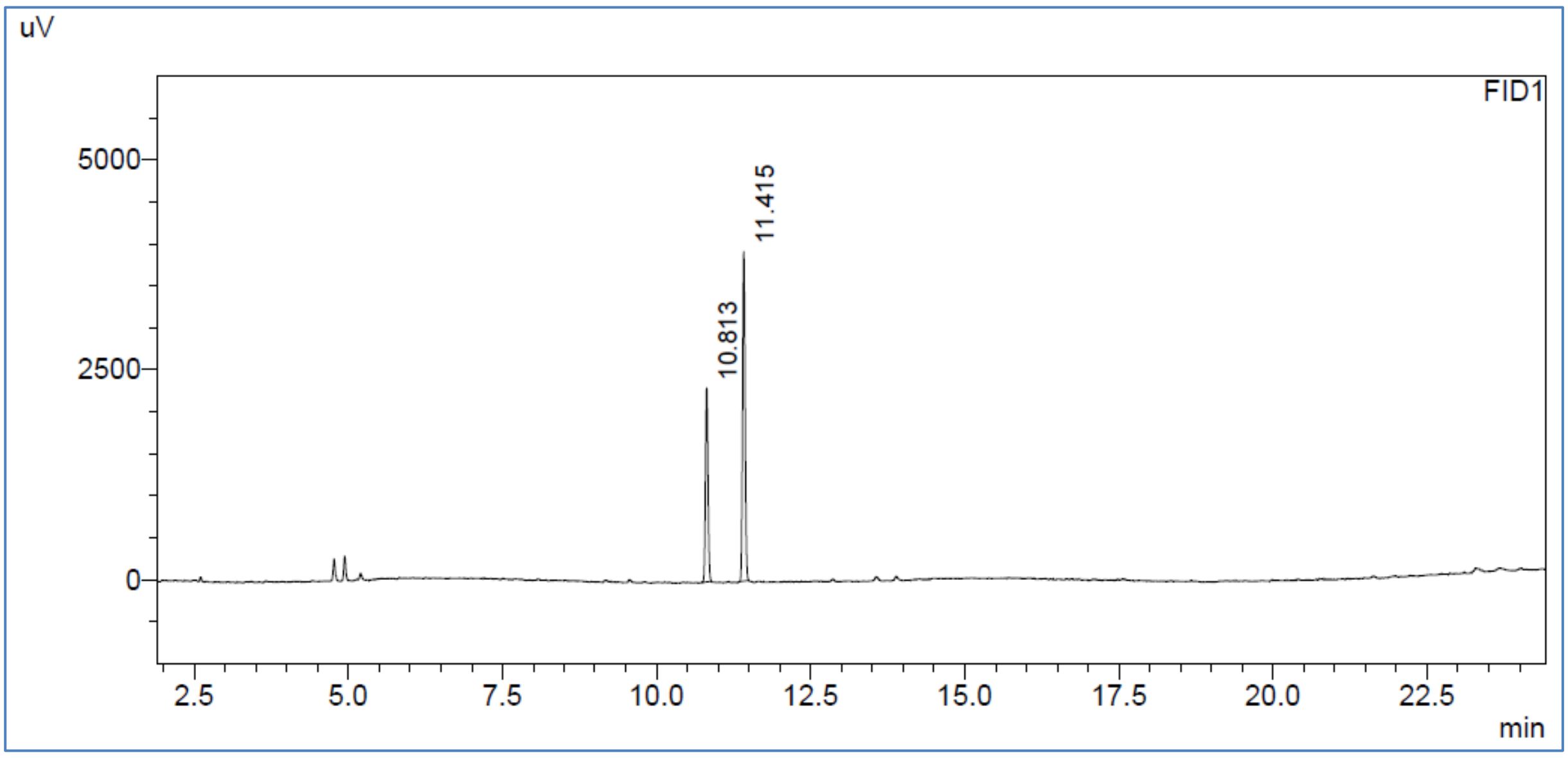

Figure S40: GC-FID Chromatographic profile of the citral with 32% of neral (10.8 min.) and 63% of geranial (11.4 min.). Very minor peaks at five minutes are solvent impurities (hexane).

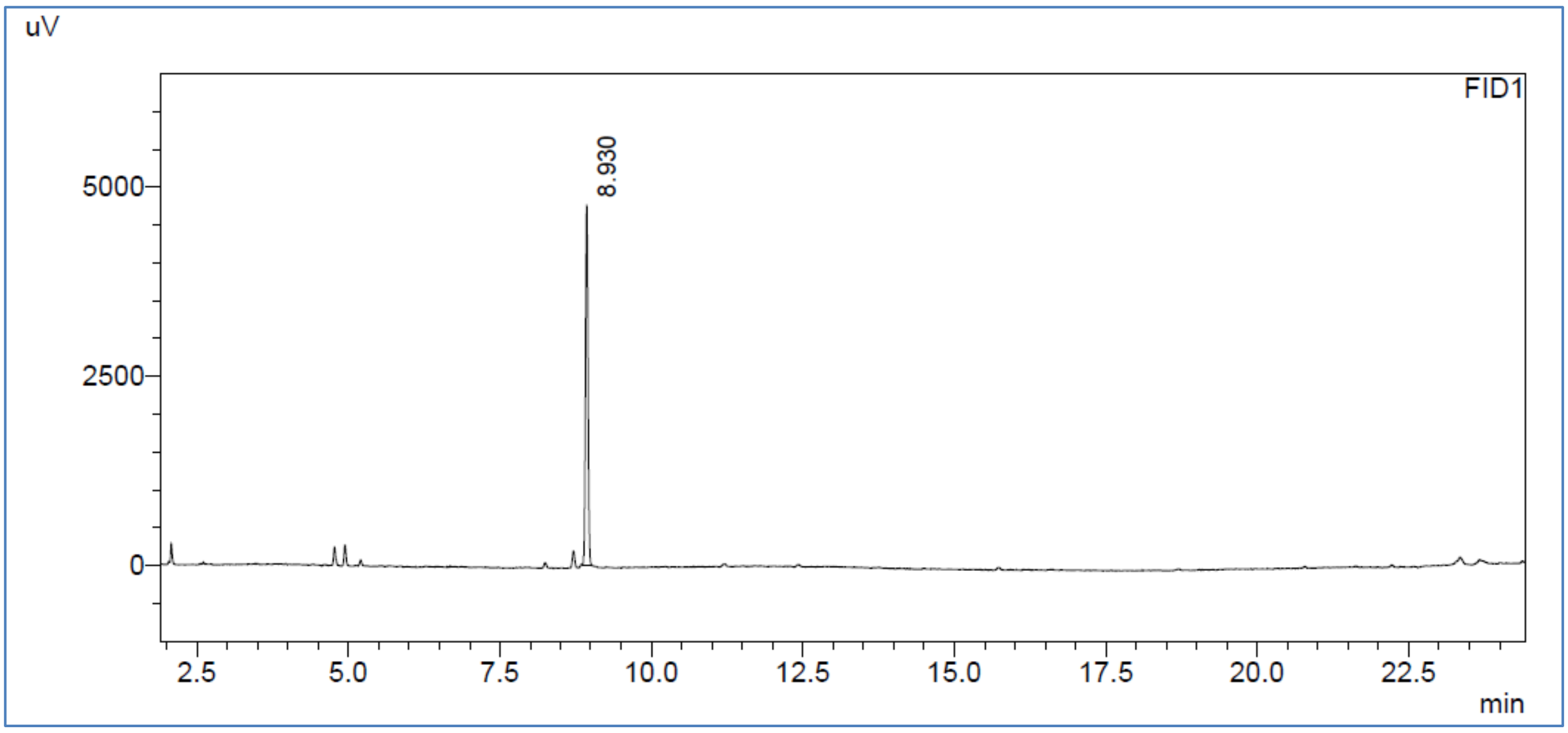

Figure S41: GC-FID Chromatographic profile of the citronellal with 95% purity. Very minor peaks at five minutes are solvent impurities (hexane).

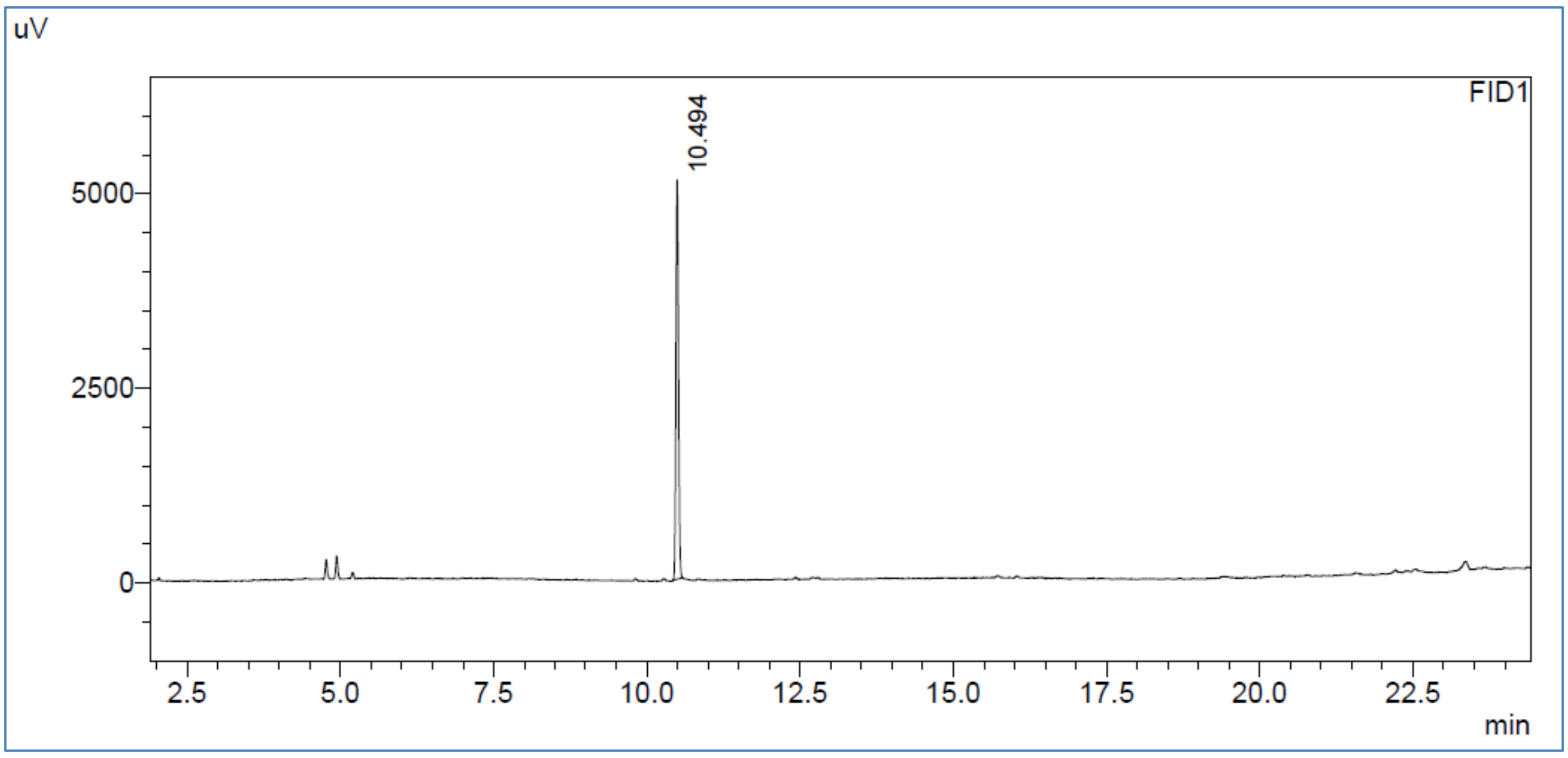

Figure S42: GC-FID Chromatographic profile of the citronellol with 98% purity. Very minor peaks at five minutes are solvent impurities (hexane).

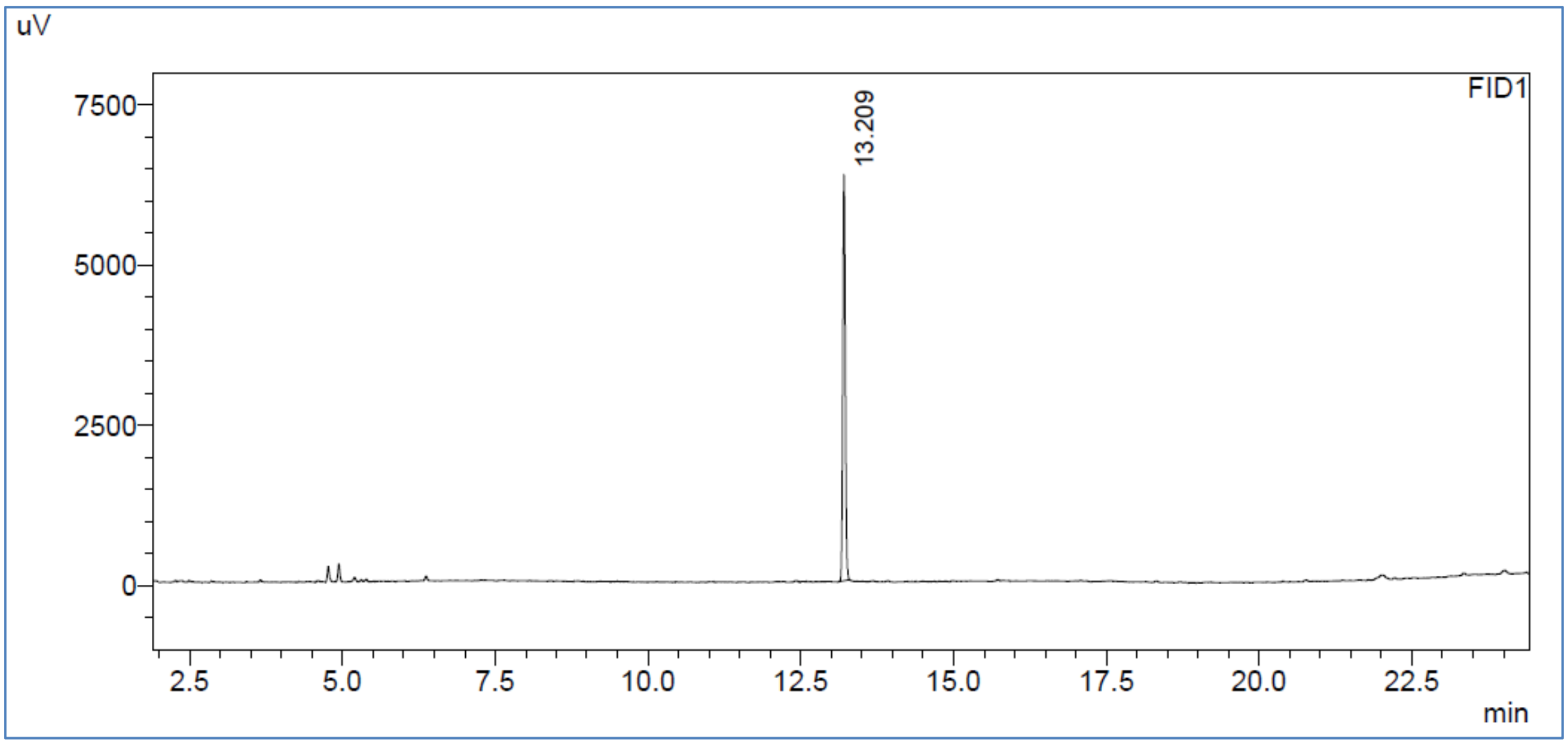

Figure S43: GC-FID Chromatographic profile of the eugenol with 98% purity. Very minor peaks at five minutes are solvent impurities (hexane).

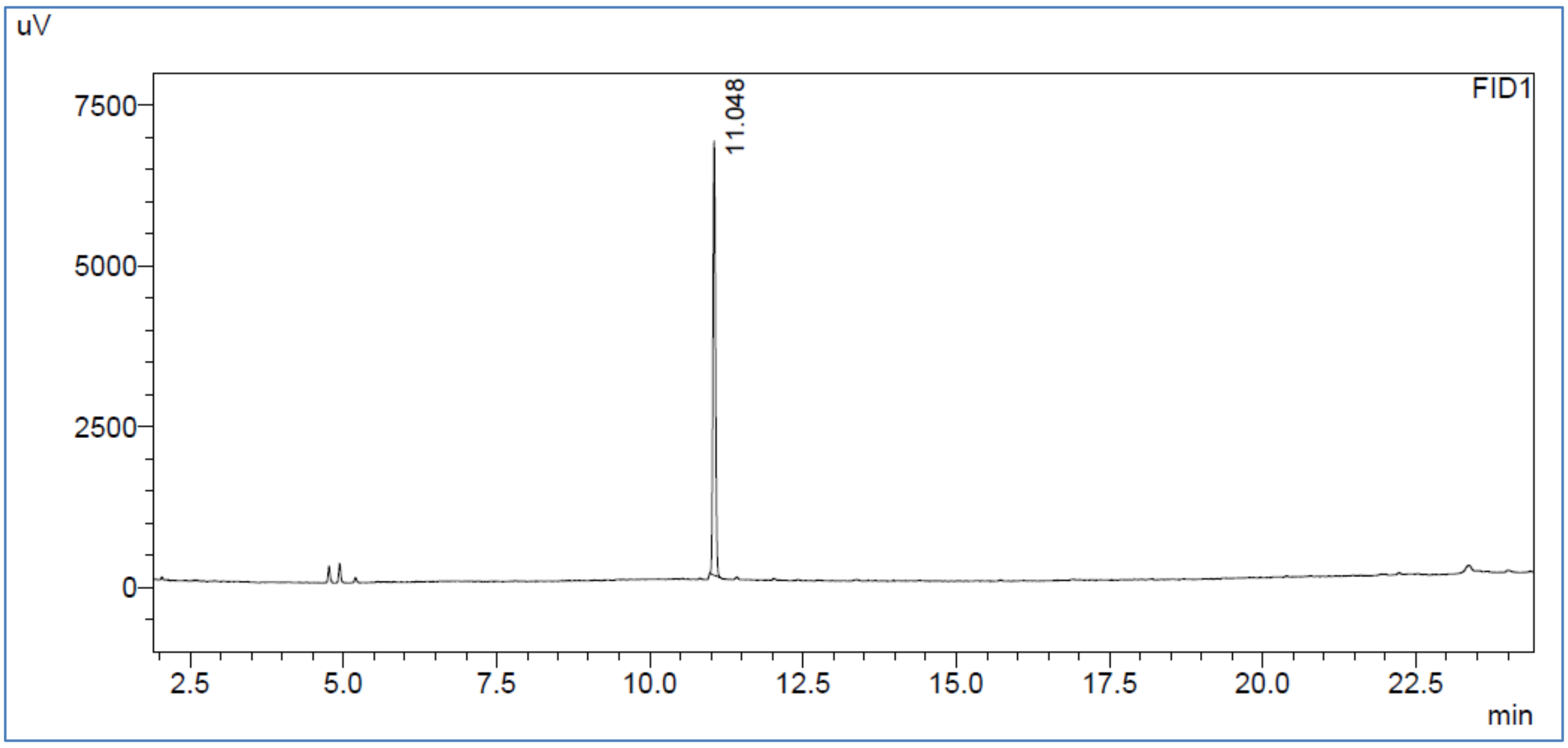

Figure S44: GC-FID Chromatographic profile of the geraniol with 98% purity. Very minor peaks at five minutes are solvent impurities (hexane).

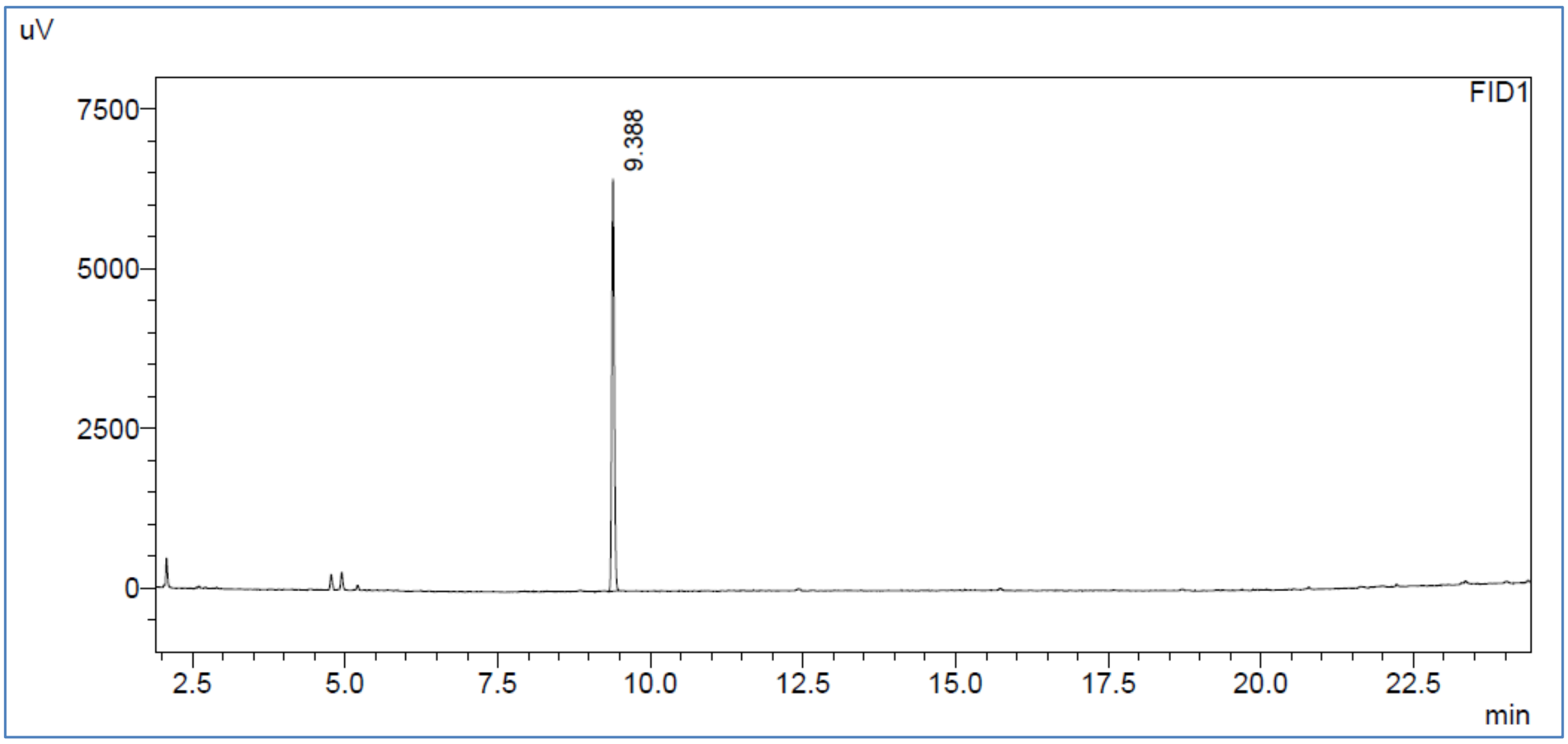

Figure S45: GC-FID Chromatographic profile of the menthol with 97% purity. Very minor peaks at five minutes are solvent impurities (hexane).

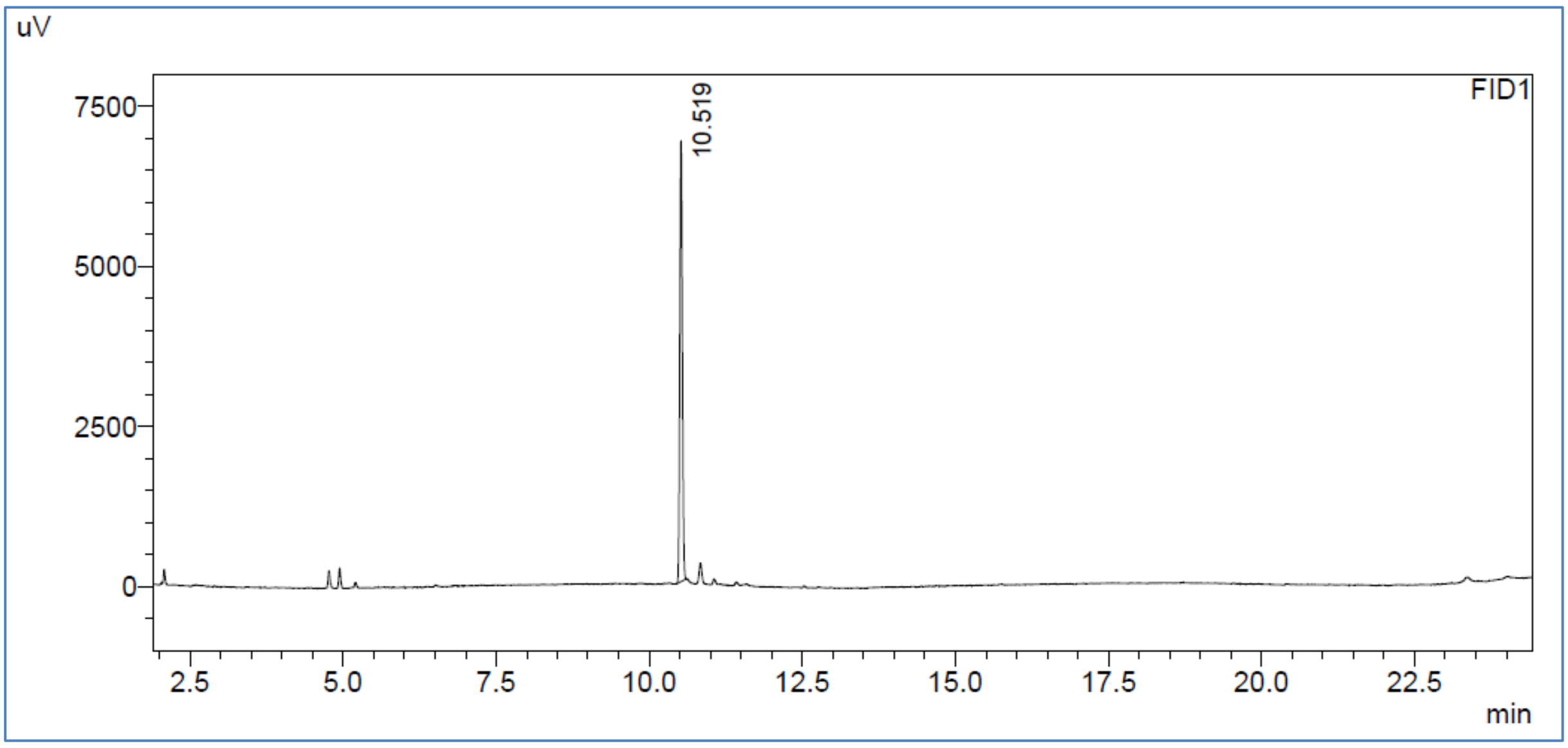

Figure S46: GC-FID Chromatographic profile of the nerol with 95% purity. Very minor peaks at five minutes are solvent impurities (hexane).

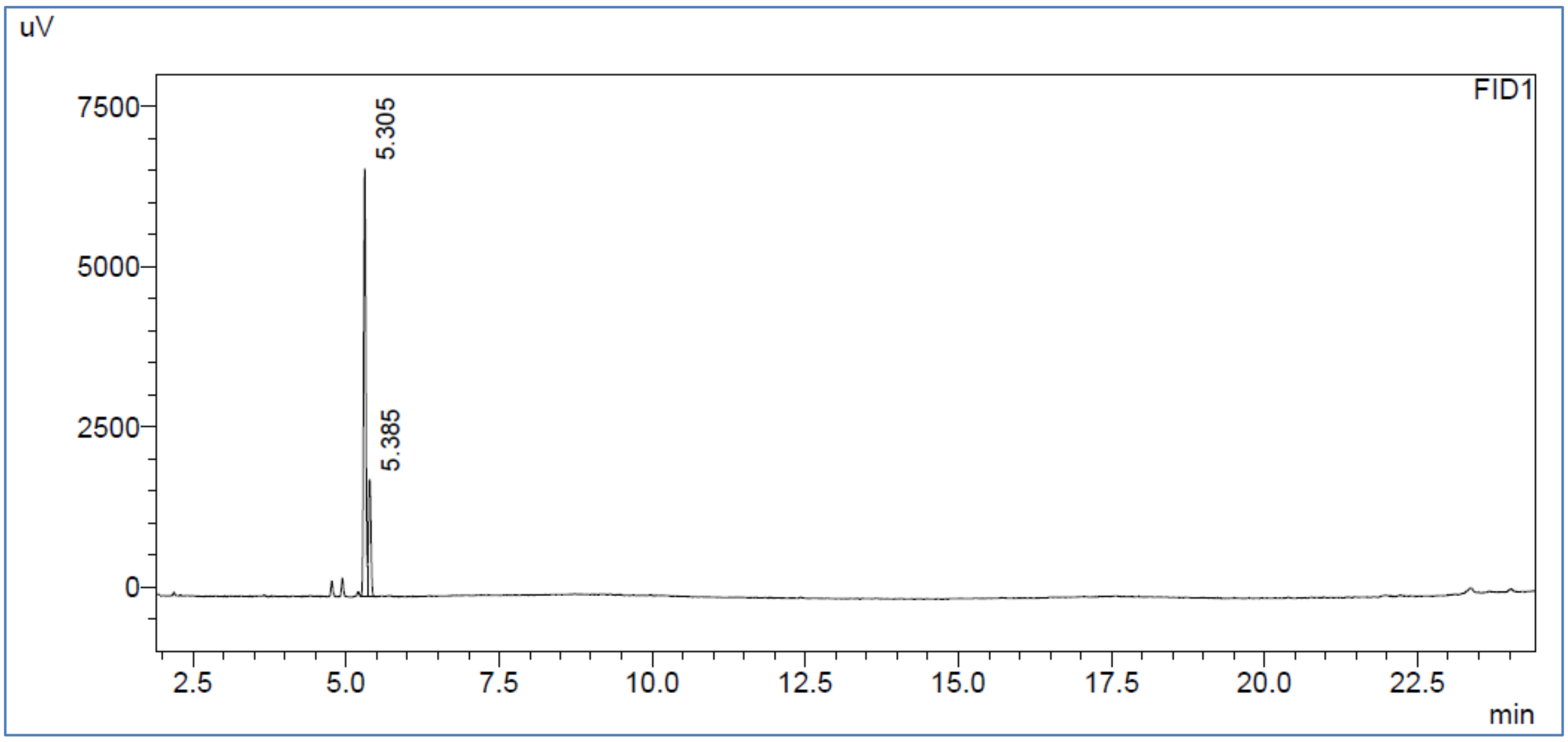

Figure S47: GC-FID Chromatographic profile with 85% of sabinene (5.3 min) and 13% of *β*-pinene (5.4 min). Very minor peaks at five minutes are solvent impurities (hexane).
